# Gene and MicroRNA Perturbations of Cellular Response to Pemetrexed Implicate Biological Networks and Enable Imputation of Response in Lung Adenocarcinoma

**DOI:** 10.1038/s41598-017-19004-3

**Published:** 2018-01-15

**Authors:** Eric R. Gamazon, Matthew R. Trendowski, Yujia Wen, Claudia Wing, Shannon M. Delaney, Won Huh, Shan Wong, Nancy J. Cox, M. Eileen Dolan

**Affiliations:** 1 Section of Hematology/Oncology, Department of Medicine, the University of Chicago, Chicago, IL 60637 USA; 2 Section of Genetic Medicine, Department of Medicine, the University of Chicago, Chicago, IL 60637 USA; 3 Present Address: Division of Genetic Medicine, Vanderbilt University School of Medicine, Nashville, TN 37232 USA

## Abstract

Pemetrexed is indicated for non-small cell lung carcinoma and mesothelioma, but often has limited efficacy due to drug resistance. To probe the molecular mechanisms underlying chemotherapeutic response, we performed mRNA and microRNA (miRNA) expression profiling of pemetrexed treated and untreated lymphoblastoid cell lines (LCLs) and applied a hierarchical Bayesian method. We identified genetic variation associated with gene expression in human lung tissue for the most significant differentially expressed genes (Benjamini-Hochberg [BH] adjusted p < 0.05) using the Genotype-Tissue Expression data and found evidence for their clinical relevance using integrated molecular profiling and lung adenocarcinoma survival data from The Cancer Genome Atlas project. We identified 39 miRNAs with significant differential expression (BH adjusted p < 0.05) in LCLs. We developed a gene expression based imputation model of drug sensitivity, quantified its prediction performance, and found a significant correlation of the imputed phenotype generated from expression data with survival time in lung adenocarcinoma patients. Differentially expressed genes (*MTHFD2* and *SUFU*) that are putative targets of differentially expressed miRNAs also showed differential perturbation in A549 fusion lung tumor cells with further replication in A549 cells. Our study suggests pemetrexed may be used in combination with agents that target miRNAs to increase its cytotoxicity.

## Introduction

Thoracic malignancies of the lung and mesothelium are among the most difficult to treat cancers in the clinical setting. Despite recent advances in molecular targeted agents and immunotherapies^1–3^, most patients are either refractory or develop resistance to treatment, and prognoses for the various subtypes of lung carcinoma and mesothelioma are typically poor unless found in an early stage. One of the most commonly used cytotoxic chemotherapeutic agents for the treatment of non-small cell lung carcinoma (NSCLC) and mesothelioma is pemetrexed, an antifolate that inhibits three enzymes used in purine and pyrimidine synthesis, thymidylate synthase (TS), dihydrofolate reductase (DHFR), and glycinamide ribonucleotide formyltransferase (GARFT), consequently suppressing DNA and RNA synthesis^4,5^. Pemetrexed has been FDA-approved as a single agent for second-line treatment of NSCLC, but is often used in combination with a platinating agent (cisplatin or carboplatin) in continuation maintenance, as well as switch maintenance therapeutic strategies, with erlotinib that have potentiated improved overall survival in NSCLC^6^. Similarly, pemetrexed in combination with cisplatin is approved as first line therapy for malignant pleural mesothelioma for patients whose disease is either unresectable or who are not otherwise candidates for curative surgery^5^.

Nevertheless, resistance to pemetrexed is a major clinical dilemma, and only limited data are available to ascertain how tumors develop inherent or acquired resistance. Over the past several years, miRNAs have become extensively examined for their role in carcinogenesis, as these key post-transcriptional gene regulators are known to affect many cellular processes, including drug resistance^7,8^. Specifically, recent studies have demonstrated that dysregulation of specific miRNAs can lead to drug resistance in different cancers, and modulation of these miRNAs using miRNA mimics or antagomiRs can have therapeutic impact on regulatory networks and signaling pathways, thereby sensitizing neoplastic cells to chemotherapy^7^.

Despite these recent findings, there is a paucity of data on the effects of miRNA regulation on global gene expression following drug treatment, as identifying the regulatory targets of miRNAs before and after administration of the agent remains a notable challenge. For our “discovery” approach, we evaluated global mRNA and miRNA changes following treatment with pemetrexed in lymphoblastoid cell lines (LCLs). The simultaneous measurements of gene transcription and miRNA expression provide a framework for a systems analysis of the effect of drug perturbation. We identify 39 differentially expressed miRNAs and show that affected genes cluster into biological networks of already known pathways relevant for pemetrexed. We then identified potential genetic mechanisms using expression quantitative trait loci (eQTL) mapping in human lung samples, and evaluated the clinical relevance of our findings in lung adenocarcinoma samples using The Cancer Genome Atlas (TCGA) and performed replication of the implicated mRNAs using A549 human lung adenocarcinoma cells.

## Results

### Global assessment of differential mRNA and miRNA expression

**Figure 1:**
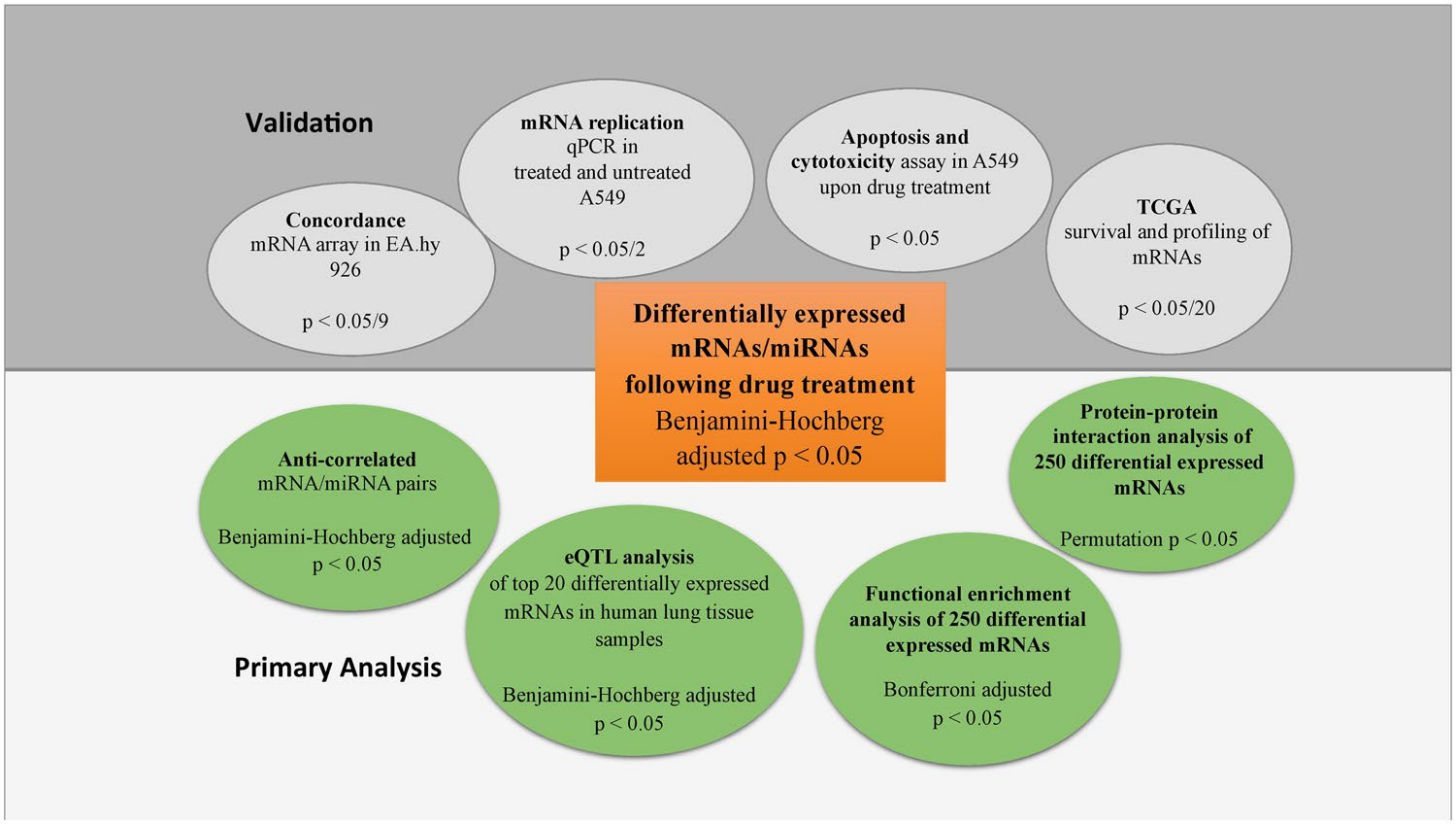
Schematic diagram of analyses and experiments performed. The primary analysis workflow starts with the differential expression analysis in LCLs following drug treatment (orange box), progressing to identification of inversely correlated miRNA-mRNA pairs, eQTL analysis in human lungs for the differentially expressed mRNAs, functional annotation enrichment (DAVID) analysis, and protein-protein interaction analysis (green circles). The subsequent validation performed includes mRNA replication using an independent array, plus the qPCR in treated and untreated A549 cells, apoptosis and cytotoxicity assay in A549 cells upon drug treatment, and TCGA profile analysis of mRNAs (gray circles).

We assessed drug-induced changes in mRNA and miRNA expression relative to untreated LCLs for each of these 11 cell lines (Supplemental Fig. 1). Figure 1 is a diagram illustrating the analytic and functional validation workflow for our study.

We found that pemetrexed treatment caused a significant alteration of mRNA expression in the cell lines (Supplemental Table 1 for list of mRNAs with Benjamini-Hochberg [BH] adjusted p-value < 0.05). A heatmap of the differentially expressed mRNAs across the paired samples (untreated and treated) illustrates the pattern of gene expression alteration following pemetrexed exposure (Supplemental Fig. 2). Overall, the probe intensities across the 3 replicates for each of the 22 samples (pemetrexed treated or untreated) were similar, showing a high level of reproducibility (Supplemental Fig.3).

**Figure 2:**
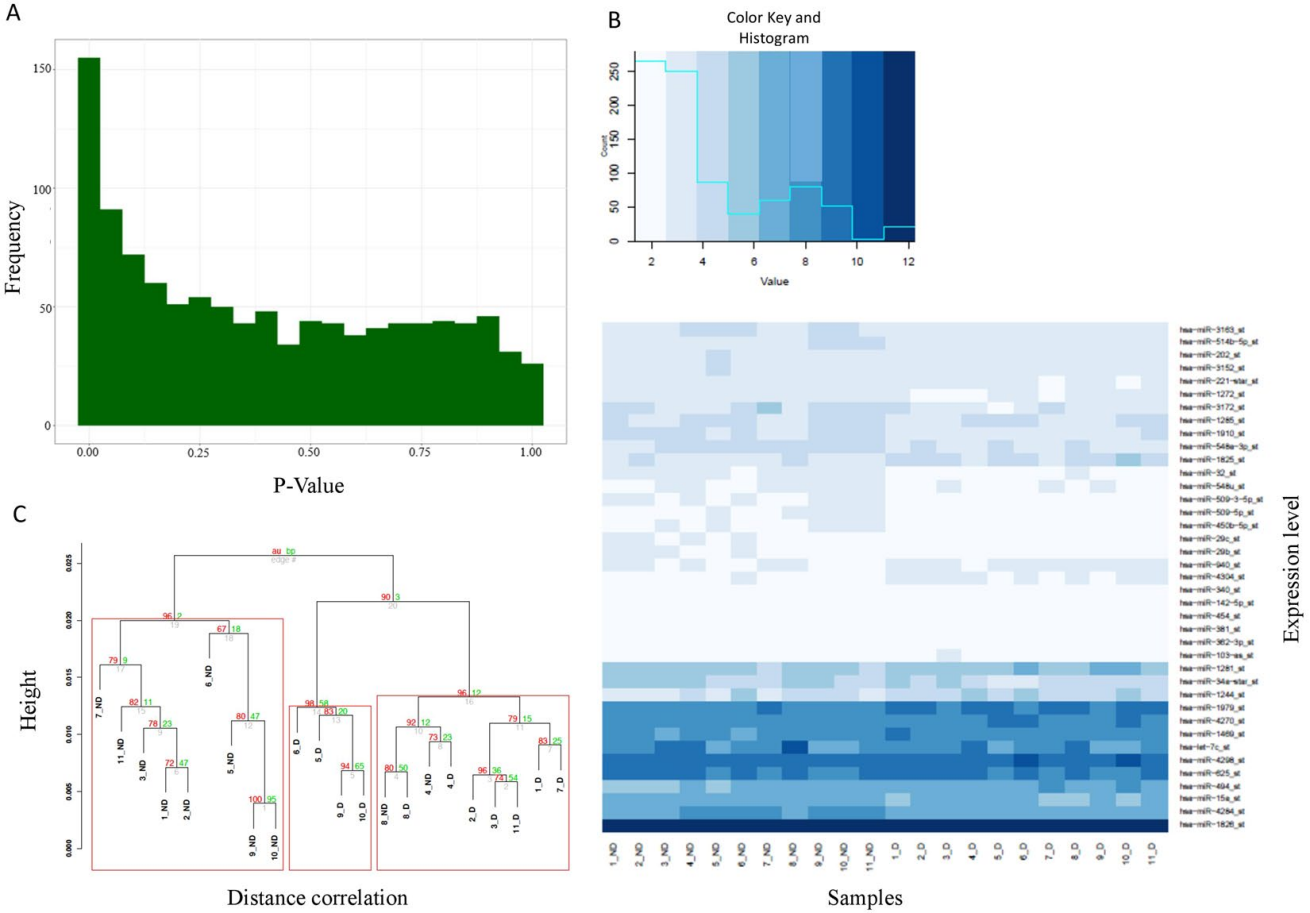
Differentially expressed miRNAs in LCLs after pemetrexed treatment. (**A**) The distribution of p-values from the differential expression analysis conducted using *limma*. Note the enrichment for low p-values among the miRNAs. (**B**) Expression pattern of miRNA expression after drug treatment. The heatmap shows the 39 significant miRNAs (BH adjusted p < 0.05) from the analyses of differential expression between pemetrexed treated and untreated LCL lines. The rows are miRNAs and the columns are the cell lines with the untreated samples listed first (1_ND-11_ND), and then the corresponding pemetrexed treated samples (1_D-11_D). (**C**) Stability of the clusters. Multiscale bootstrap resampling (N = 1000 bootstrap replicates) quantifies the uncertainty in the clusters. AU (in red) is the “Approximately Unbiased” probability while BP (green) is the “Bootstrap Probability”. The red rectangle shows the clusters for which the null hypothesis is rejected at the significance level of 0.05.

Pemetrexed treatment also induced a significant change of miRNA expression in the cell lines (Supplemental Table 2 for list of top 39 miRNAs with BH adjusted p < 0.05 and Fig. 2A for the overall p-value distribution, indicating enrichment for low p-values). Strikingly, a heatmap of the alterations in miRNA expression following administration of pemetrexed showed a more pronounced separation between pemetrexed treated and untreated samples than the mRNA expression signature (Fig. 2B and Supplemental Fig.2A).

To assess the uncertainty in the clusters, we obtained an “Approximately Unbiased” p-value and a “Bootstrap Probability” value for each cluster (Fig. 2C and Supplemental Fig. 2B) from multiscale bootstrap resampling (see Methods), demonstrating the existence of stable clusters, primarily defined by drug treatment, from the miRNA, but not from the mRNA, expression data.

Similar to mRNA, the quantified expression intensities for the miRNAs showed a high level of reproducibility across the 3 replicates of each pemetrexed treated and untreated sample (Supplemental Fig. 4). The 20 most significant mRNAs and miRNAs (BH adjusted p < 0.05 for each RNA type) after pemetrexed exposure, along with log Fold Change (logFC), the *B*-statistic, and p-value, are listed in Tables 1 and 2, respectively. The top 8 mRNAs and top 8 miRNAs were also significant after Bonferroni adjustment.

**Table 1:** The twenty most significantly altered gene expression traits (mRNA) in LCLs after exposure to pemetrexed.

logFC	p-value	B-statistic	Gene
0.21	2.62E-07	7.06	*ZFAND1*
−0.11	1.31E-06	5.63	*LBR*
0.13	1.57E-06	5.47	*UCHL3*
0.15	1.67E-06	5.41	*FAM171B*
0.16	1.73E-06	5.38	*SFT2D1*
0.15	1.8E-06	5.35	*TMEM60*
0.15	2.13E-06	5.20	*C4orf33*
0.14	2.2E-06	5.17	*NDUFB5*
0.15	2.44E-06	5.07	*LHFP*
−0.19	2.46E-06	5.06	*NARS*
0.12	2.52E-06	5.04	*ZNF426*
0.18	2.67E-06	4.99	*WBP4*
0.11	2.93E-06	4.90	*UCHL1*
0.12	3.44E-06	4.76	*CA5B*
−0.16	3.46E-06	4.75	*AHCTF1*
−0.13	3.75E-06	4.68	*BOP1*
0.12	3.81E-06	4.66	*MTFMT*
0.17	4.17E-06	4.58	*PSMC6*
0.12	4.17E-06	4.58	*C16orf80*
−0.13	4.32E-06	4.55	*TCEA1*

**Table 2:** The top twenty microRNAs with significantly altered expression in LCLs after exposure to pemetrexed.

logFC	p-value	B-statistic	microRNA
0.98	3.53E-08	9.07	hsa-miR-1244
0.74	2.34E-07	7.34	hsa-miR-494
0.45	6.83E-07	6.34	hsa-miR-1979
0.31	3.97E-06	4.66	hsa-miR-1826
0.77	9.14E-06	3.85	hsa-miR-1281
−0.31	1.13E-05	3.65	hsa-miR-202
−0.37	1.14E-05	3.64	hsa-miR-4284
−0.49	1.70E-05	3.25	hsa-miR-221-star
−0.89	6.51E-05	1.94	hsa-miR-3172
−0.63	9.48E-05	1.57	hsa-miR-548a-3p
−0.43	1.02E-04	1.49	hsa-miR-1272
−0.34	1.29E-04	1.27	hsa-miR-15a
−0.40	1.59E-04	1.06	hsa-miR-3152
−0.22	2.56E-04	0.59	hsa-miR-142–5p
0.24	3.03E-04	0.43	hsa-miR-4270
−0.29	3.27E-04	0.35	hsa-miR-1910
−0.42	3.29E-04	0.35	hsa-miR-34a-star
−0.18	3.78E-04	0.21	hsa-miR-381
−0.30	4.10E-04	0.13	hsa-miR-450b-5p
0.30	4.34E-04	0.08	hsa-miR-1469

### Associations between differentially expressed miRNAs and differentially expressed mRNAs

**Figure 3:**
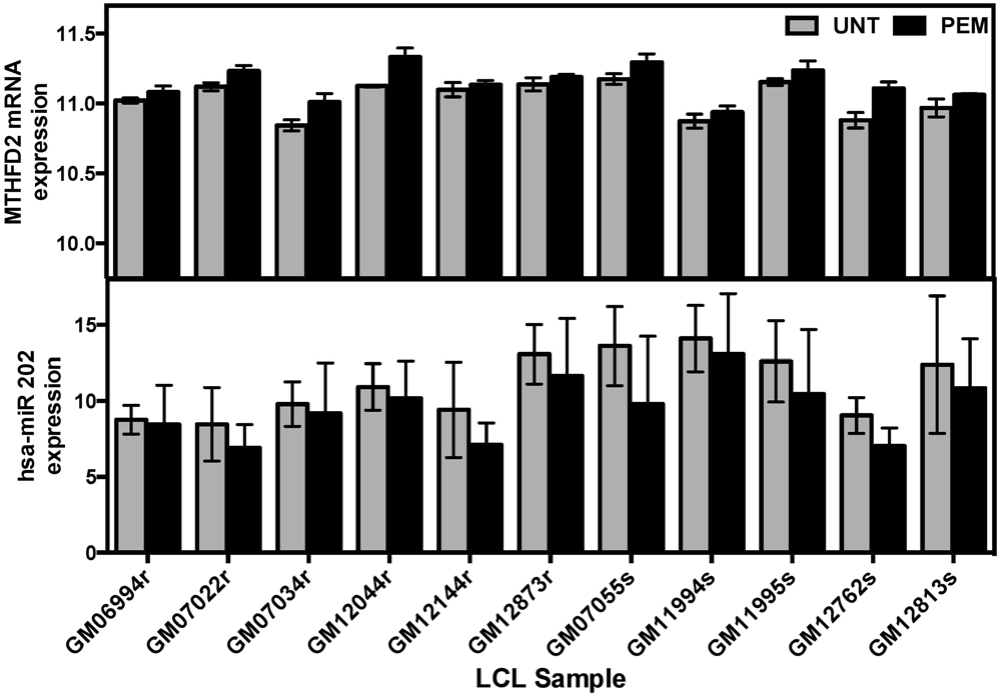
*MTHFD2* and hsa-miR-202 expression in pemetrexed treated and untreated LCL samples. The miRNA hsa-miR-202 is a putative regulator of *MTHFD2*. Consistently across all 11 LCL cell lines, *MTHFD2* (expressed as an average of probeset ID 8042830 & 8084064) showed increased expression whereas hsa-miR-202 showed decreased expression after pemetrexed exposure.

We also looked for differentially expressed miRNAs (BH adjusted p < 0.05) that may target differentially expressed mRNAs (BH adjusted p < 0.05). Towards this end, we utilized the miRNA target prediction algorithm ExprTarget^9^, which combines evidence from various computational approaches. The ExprTarget score is a function of the weighted sum of the scores from select computational algorithms for miRNA target prediction. Furthermore, we required that the miRNA and mRNA pairs of gene expression be negatively correlated (p < 0.05) in an analysis of baseline expression^9^. Supplemental Table 3 shows the correlated miRNAs and mRNAs with their p-values from the analyses of differential expression after pemetrexed exposure, including the pair *MTHFD2 (*methylenetetrahydrofolate dehydrogenase, p = 1.46 × 10^−5^) and hsa-miR-202 (p = 1.13 × 10^−5^), as well as *SUFU* (pair suppressor of fused homolog, p = 1.1 × 10^−4^) and hsa-miR-494 (p = 2.34 × 10^−7^). Consistently across all 11 cell lines, pemetrexed treatment resulted in an increase in expression levels of *MTHFD2* with a corresponding decrease in hsa-miR-202 levels (Fig. 3).

### Genetic regulation of differentially expressed mRNAs

To identify potential genetic mechanisms underlying the expression perturbations due to pemetrexed exposure, we annotated the differentially expressed mRNAs with (cis-acting) eQTL information from the Genotype-Tissue Expression (GTEx) project^10,11^. Of the 20 most significantly altered genes after drug treatment (Table 1), nine genes – *AHCTF1*, *C4orf33*, *SFT2D1*, *TMEM60*, *ZFAND1*, *LHFP*, *WBP4*, *UCHL3*, *NARS* – were found to have significant cis-acting eQTLs in human lung tissue^12^. These eQTLs (Supplemental Table 4) are prime candidates for future clinical studies of pemetrexed response.

### Functional analysis of the differentially expressed mRNAs

In evaluating the top differentially expressed mRNAs (n = 250), we found a highly significant enrichment for several functional annotations (Supplemental Fig. 5), including *acetylation* (genes post-translationally modified by the attachment of at least one acetyl group; n = 85 genes; Fold enrichment = 2.2; Bonferroni-adjusted p = 4.2 × 10^−11^), *mitochondrion* (the site of tissue respiration; n = 37 genes; Fold enrichment = 2.33; Bonferroni-adjusted p = 6.8 × 10^−4^), and *phosphoprotein* (genes post-translationally modified by the attachment of either a single phosphate group, or of a complex molecule, such as 5′-phospho-DNA, through a phosphate group; n = 119 genes; Fold enrichment = 1.29; Bonferroni-adjusted p = 0.017). The following functional annotations were found to be nominally enriched (p < 0.05) for differentially expressed genes: *Pyruvate metabolism* (p = 0.012), *One carbon pool by folate* (p = 0.023) and *proteasome complex* (p = 0.034).

### Protein-protein interaction analysis

**Figure 4:**
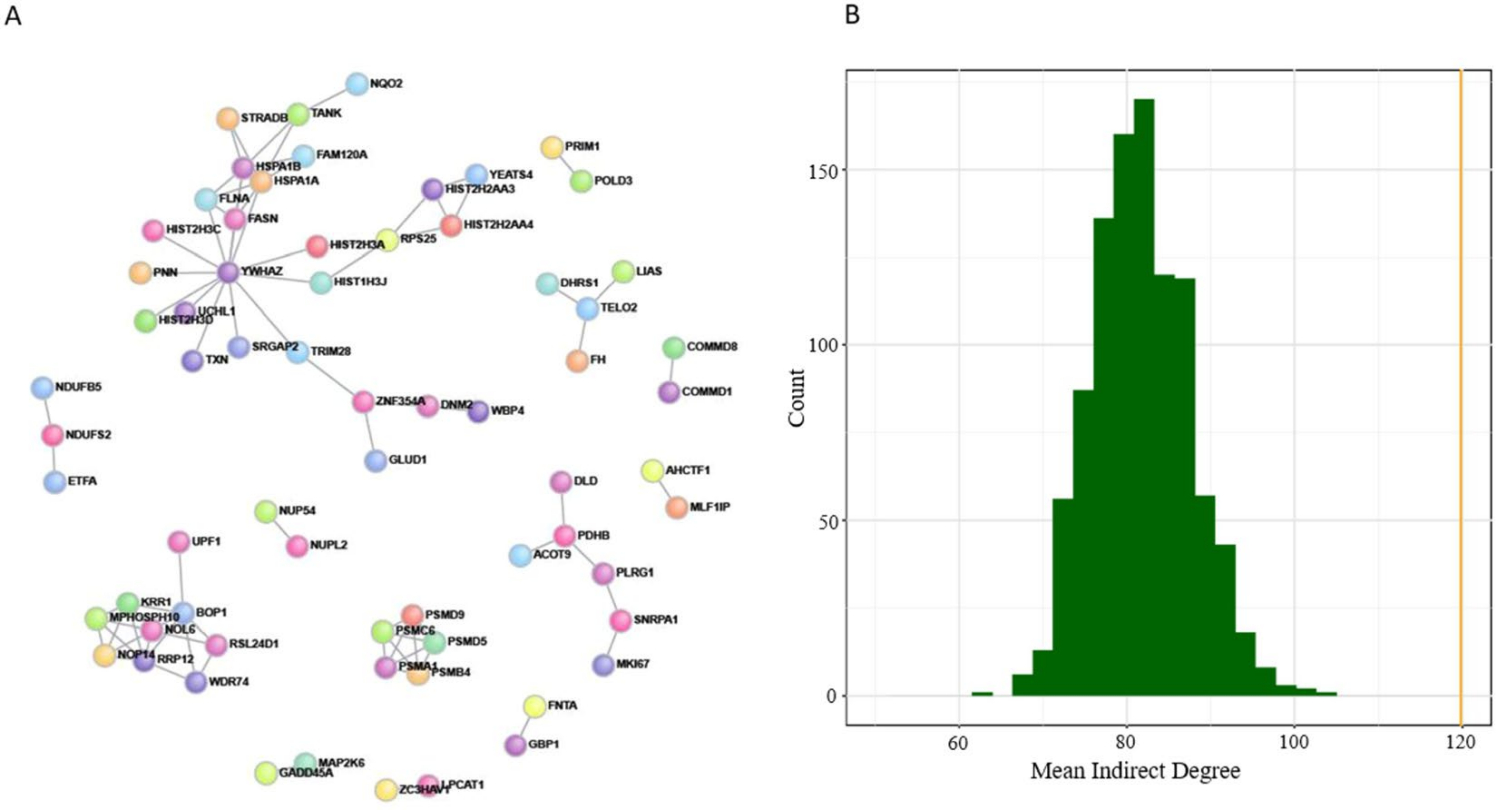
Differentially expressed genes and protein-protein interaction analysis. (**A**) The differentially expressed genes showed a high degree of network connectivity. The approach used here (DAPPLE) to quantify connectivity required not only evidence of direct interaction *in vitro*, but also co-expression of the genes in a tissue. We found that 83 edges or direct connections were found when 67 were expected, which was significant enrichment (p = 0.04) based on 1,000 within-degree node-label permutations. (**B**) The mean indirect degree was 119.8, which was highly significant (p < 0.001). The histogram shows the permutation null distribution of the mean indirect degree. The vertical line (orange) is the observed mean indirect degree, indicating a greater number of indirect interactions than expected.

These same 250 differentially expressed mRNAs (Supplemental Table 1) showed a high degree of network connectivity (Fig. 4A). The approach used here to quantify connectivity^13^ required not only evidence of direct interaction *in vitro*, but also co-expression of the genes in a tissue. Notably, 83 edges or direct connections were found among the top differentially expressed mRNAs when 67 were expected, indicative of a highly significant enrichment (p = 0.04) based on a within-degree node-label permutation^13^. The mean direct degree for the differentially expressed genes was 2.48, significantly more than expected by chance (p = 0.03), suggesting that the proteins encoded by these genes were more densely connected. The mean indirect degree was 119.8 (expected value = 81.8), which was highly significant (p < 0.001; Fig. 4B). Supplemental Table 5 shows the probability that the gene would be as connected to other differentially expressed genes as observed by chance. Notably, the differentially expressed genes that were found to be the most highly connected (p < 0.05) to other differentially expressed genes were enriched for the proteasome (*PSMC6*, *PSMD5*, *PSMD9*, *PSMA1*, *PSMB4*, and *UCHL1*; Bonferroni-adjusted p = 6.6 × 10^−5^), a protein complex important for degradation of proteins destined for destruction by ubiquitination or by other targeting mechanisms.

### mRNA replication using an independent microarray experiment and qPCR in A549 cells

*MTHFD2* and *SUFU* were among the differentially expressed mRNAs putatively targeted by miRNAs, with significantly altered expression after pemetrexed treatment (Supplemental Table 3). We sought to replicate these findings using an independent microarray experiment by evaluating the results of a study of the effect of pemetrexed treatment on EA.hy 926 cells (a fusion of human umbilical vascular endothelial cells and A549)^14^. The two probes (8042830 and 8084064) for *MTHFD2* showed highly significant differential expression with concordant direction of effect (p = 7.62 × 10^−4^ and p = 1.56 × 10^−3^, respectively), as was observed in the LCLs following treatment with pemetrexed. Similarly, a probe (7930120) for *SUFU* was differentially expressed with consistent direction of effect (p = 1.64 × 10^−3^), as was observed in the LCLs.

**Figure 5:**
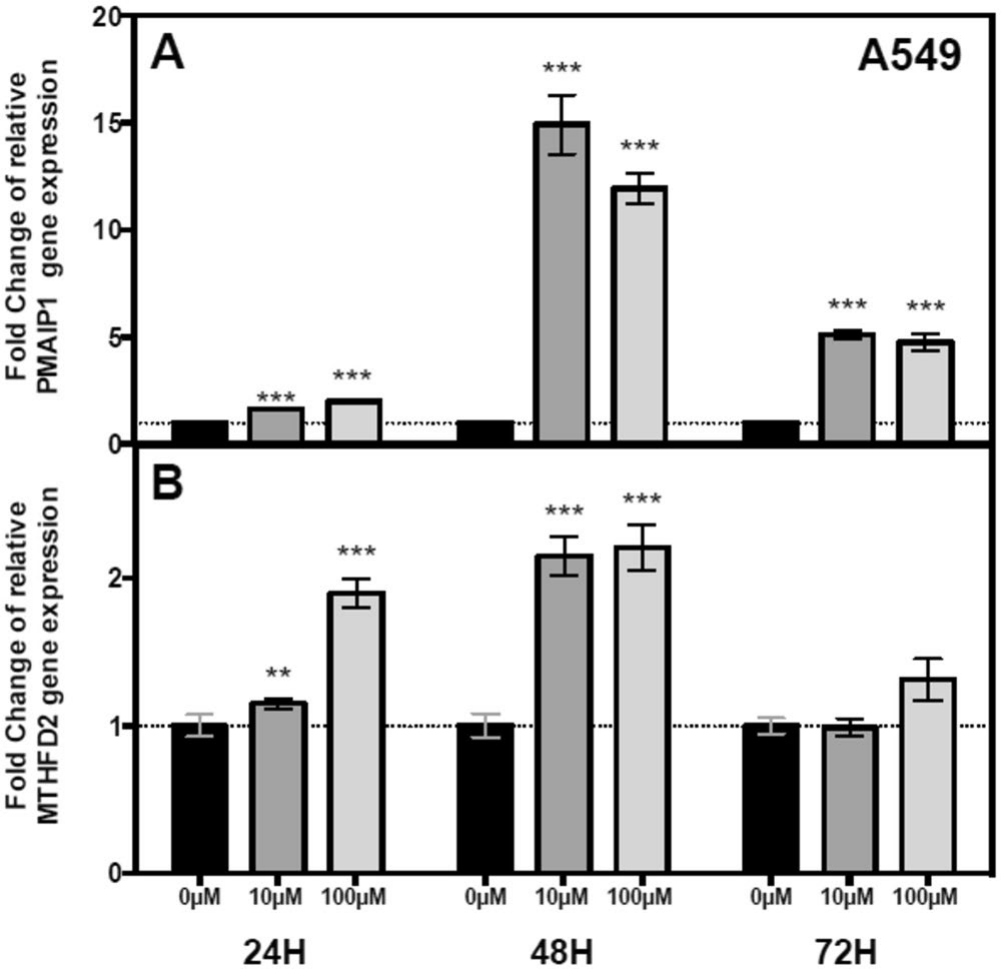
Gene expression of *PMAIP1* and *MTHFD2* in A549 cells after pemetrexed treatment. A549 cells were treated with 0, 10 and 100 µM pemetrexed and collected at 24, 48 and 72 hours post treatment. Following treatment with 10 µM and 100 µM pemetrexed, *PMAIP1* (**A**) gene expression was significantly upregulated at 24, 48 and 72 hours whereas *MTHFD2* (**B**) gene expression was significantly upregulated at 24 and 48 hours (**p < 0.01; ***p < 0.001).

We also performed qPCR in pemetrexed treated and untreated A549 cells for the two replicated differentially expressed genes that are putative targets of differentially expressed miRNAs (*MTHFD2*) and the pro-apoptotic gene *PMAIP1* (phorbol-12-myristate-13-acetate-induced protein 1, also known as Noxa, p = 5.77 × 10^−6^, BH adjusted p = 0.005) in A549 cells (Fig. 5). We found significant increases in gene expression for both genes 48 hours following treatment with pemetrexed. Taken together, these differential expression changes suggest substantial concordance between the results obtained in LCLs and A549 lung carcinoma cells in response to pemetrexed.

### Apoptosis and survival in pemetrexed treated A549 cells

Since *PMAIP1* is a pro-apoptotic member of the Bcl-2 protein family^15^ and activates caspases by inducing mitochondrial membrane changes and efflux of apoptotic proteins from the mitochondria^16,17^, we evaluated apoptosis (as measured by caspase 3/7 activation) and survival (as measured by CellTiter-Glo) in A549 cells treated with pemetrexed for 24, 48 and 72 hours. Pemetrexed significantly affected cell survival through increased caspase 3/7 activation at 10 µM and 100 µM doses, which corresponded to observed decreased CellTiter Glo values (Supplemental Fig. 6). This change in cellular sensitivity with 10 or 100 µM pemetrexed could be due in part to higher PMAIP1 gene expression.

### Survival and molecular profiling analysis using The Cancer Genome Atlas

Since pemetrexed is used to treat NSCLC, we analyzed TCGA data to determine whether the differentially expressed genes and the enriched pathways are associated with survival parameters. *MTHFD2* inhibition has been shown to enhance the apoptotic effects of methotrexate (MTX; another antifolate that has a similar mechanism to pemetrexed) in several cancer cell lines^18^, and appeared to be a rational selection for further study. Molecular profiling analysis of lung adenocarcinoma samples (N = 230 patients) from TCGA indicates that alterations in *MTHFD2* and in two thiol proteases, *UCHL1* and *UCHL3*, in the form of amplification or mRNA upregulation, are associated with shorter median survival (although drug regimen is not reported in this dataset). Among the lung adenocarcinoma samples, 7% (17 out of 230 patients) showed *MTHFD2* alterations (amplification, mutation, or mRNA upregulation). For example, Supplemental Figure 7 illustrates the mRNA upregulation due to copy number alteration for the gene in the TCGA subjects. We then considered a subgroup of patients with survival data. Of the 17 cases with *MTHFD2* alterations, 8 were deceased with median month survival of 23.36 months. By contrast, of the 188 cases without such alterations, 55 were deceased with median month survival being 45.31 months. Amplification or upregulation of the gene was associated with shorter median survival (one-sided logrank test p-value = 0.06).

TCGA data for lung adenocarcinoma showed amplifications in 13% of the cases for thiol proteases *UCHL1* and *UCHL3*. Of the 30 cases with alterations in these genes, 12 were deceased with median month survival of 32.07 months. By contrast, 51/176 cases without such alterations resulted in a median month survival of 45.31 months. Amplification in these genes was associated with shortened median survival (p = 0.041).

We tested the top 20 differentially expressed genes (Table 1) for association with shorter median survival. Of these, 18 could be tested in the TCGA expression data for lung adenocarcinoma (Supplemental Table 6), and 3 met only nominal significance (logrank test p-value < 0.05): *NARS* (p = 0.03), an eQTL target gene in lung, as well as *ZNF426* (p = 0.02), and *TCEA1* (p = 0.023).

### Model based imputation of drug response

**Figure 6:**
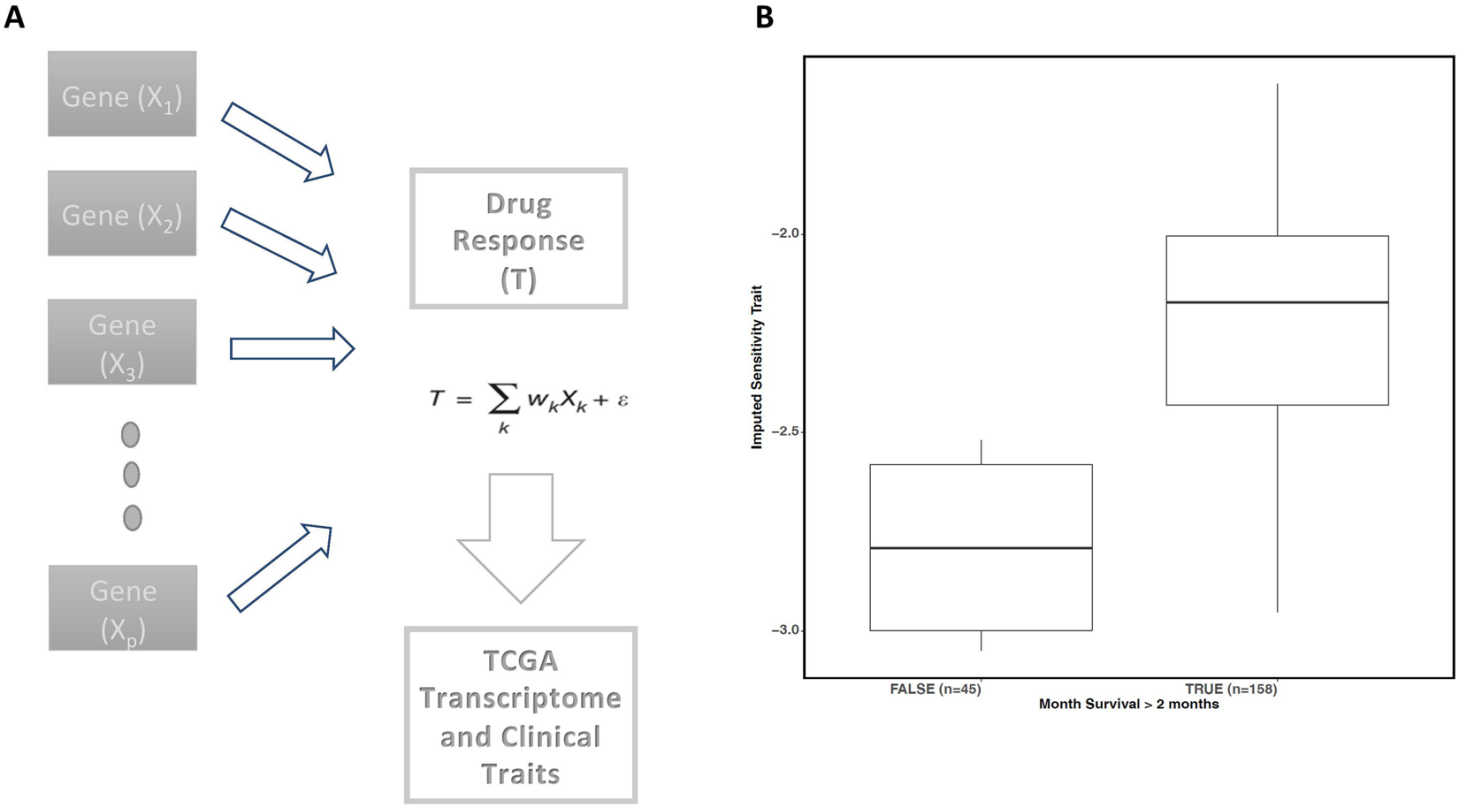
Drug response imputation model using gene expression data evaluated in TCGA dataset for response. (**A**) Drug response imputation model developed using gene expression data. The model consists of the additive non-zero effect of a set of genes. (**B**) The model was applied to tumor expression data in TCGA to impute drug response in the TCGA samples in order to correlate the novel phenotype with survival time. The imputed sensitivity phenotype was significantly associated (p = 0.006) with survival time with resistant samples showing significantly lower survival time.

We developed a gene expression based imputation model^19,20^ of drug response using machine learning applied to the expression data in the cell lines (Fig. 6A, and Supplemental Table 7). The R^2^ between observed and predicted trait from the imputation model was 0.89 (p = 1.8 × 10^−11^). Although directly measured drug response data are generally not available for the TCGA samples, the gene expression-derived phenotype developed in a training dataset (such as from cell lines^21^) is a novel molecular trait that can be tested for its ability to predict clinical parameters in an independent target dataset. We applied the model to the TCGA lung adenocarcinoma tumor expression data. We found that the imputed sensitivity trait was associated with survival time (Fig. 6B), with patients having survival time of less than 2 months showing significantly lower imputed sensitivity or higher imputed resistance to pemetrexed (Mann Whitney U test, p = 0.006). Additional replication is thus warranted.

## Discussion

Our genome-wide assessment revealed 8 mRNAs and 8 miRNAs with significant differential expression after stringent Bonferroni correction following exposure to pemetrexed. Our “discovery” study was conducted in LCLs because of the wealth of genetic^22–24^, expression^25,26^, and pharmacologic information on this cell type^27,28^. We assessed several of our top miRNA hits in A549 cells and validated several of the top mRNAs using re-analyzed whole-transcriptome data following pemetrexed treatment on EA.hy 926 cells. Furthermore, we performed eQTL analysis in human lung samples (n = 278) from the GTEx Consortium^11^, and found that nine of the top 20 most highly perturbed genes after pemetrexed exposure are under significant local genetic control by cis-acting eQTLs. One of the miRNA/mRNA pairs we identified as relevant following treatment with pemetrexed was hsa-miR-202 and one of its predicted targets *MTHFD2*. Our *in vitro* findings support a role for *MTHFD2* in determining the cellular response of LCLs and A549 cells to pemetrexed, while molecular profiling analysis of 230 lung adenocarcinoma patients from TCGA indicates that amplification or upregulation of *MTHFD2* is associated with shorter survival.

*MTHFD2* encodes a mitochondrial bifunctional enzyme with methylenetetrahydrofolate dehydrogenase and methenyltetrahydrofolate cyclohydrolase activities. The enzyme is expressed predominately in the developing embryo as an essential component of the mitochondrial folate metabolic pathway, and becomes inactivated in healthy adult tissues, even in labile cell populations^29^. However, it has been recently elucidated that MTHFD2 is highly expressed in a variety of malignancies (including lung carcinoma^30^; has not been thoroughly evaluated in mesothelioma), as transformed cells can become dependent on folate-mediated one-carbon metabolism to support purine and thymidylate synthesis^29,31^. Further, a recent study has confirmed the crystal structure of MTHFD2 in complex with a substrate-based inhibitor^29^, indicating that the protein is targetable via small molecule administration. Therefore, MTHFD2 is a particularly intriguing drug target that may elicit a potent antineoplastic response, while conferring low toxicity to normal tissue.

In relation to the present study, MTHFD2 expression in neoplastic cells may be responsive to antifolate agents, and there is prior evidence that *MTHFD1* and *MTHFD2* affect pemetrexed response. One recent randomized clinical trial examining the efficacy of pemetrexed against malignant pleural mesothelioma indicated that patients with at least one risk allele at the *MTHFD1* nonsynonymous polymorphism rs2236225 exhibited a significantly lower response rate and shorter progression-free survival than non-carriers^32^. Further, the present study (in LCLs and A549 cells) and a previously published study (re-analyzed here as a replication set) done on the EA.hy 926 fusion cell line demonstrated a significant increase in expression in *MTHFD2* after pemetrexed exposure^14^. In addition, overexpression of genes coding for folate metabolism enzymes, including *MTHFD2*, have been shown to be indicative of rapidly proliferating tumors that are markedly sensitive to pemetrexed^18^. Using TCGA data, we determined that upregulation of MTHFD2 is associated with shorter survival, and is consistent with a previous report that MTHFD2 suppression (such as through an inhibitor) exerts antiproliferative and proapoptotic effects in cancer cell lines^18^. Importantly, the association between MTHFD2 and NSCLC response to pemetrexed has been validated *in vivo*. KRAS patient-derived xenografts that have elevated MTHFD2 expression have a greater dependency on folate metabolism due to increased purine synthesis and appear to be more sensitive to pemetrexed treatment than KRAS negative tumors^33^. These findings also suggest that MTHFD2 and potentially other folate synthesis pathway enzymes could potentially be implemented as biomarkers for the use of pemetrexed-based therapy for certain forms of lung carcinoma.

Among the mRNAs with significantly altered levels after exposure, *PMAIP1* was upregulated in all 11 cell lines (p = 5.77 × 10^−6^). *PMAIP1* (Noxa) is a pro-apoptotic member of the Bcl-2 protein family. *PMAIP1* activates caspases by inducing mitochondrial membrane changes and efflux of apoptotic proteins from the mitochondria^15–17^. Evaluation of *PMAIP1* expression in A549 cells demonstrates a 12-fold increase in expression at 48 hours following treatment with 10 µM pemetrexed. To our knowledge, the connection of *PMAIP1* to pemetrexed response is a novel finding. However, a replication study with a larger sample size will be needed to further validate this hypothesis.

Both *MTHFD2* (a member of the mitochondrial glycine biosynthetic pathway) and *PMAIP1* (known to mediate p53-dependent apoptosis via mitochondrial dysfunction) highlight the significance of the mitochondria to pemetrexed response and are consistent with recent studies that aim to therapeutically target the mitochondria in order to increase sensitivity of cancer cells to apoptosis^34^. Pemetrexed, in combination with sorafenib, has been shown to promote tumor killing through an autophagy-dependent mechanism that activates the intrinsic (mitochondria-mediated) apoptotic pathway^35^. Targeting the mitochondria may well provide an important approach for overcoming apoptosis resistance^36^, and the genes we implicate here could provide important targets for modulating pemetrexed efficacy.

In addition to several intriguing genes that demonstrated differential expression in LCLs after pemetrexed treatment, our analysis revealed miRNAs that have been previously linked to treatment response in NSCLC, and two of our top miRNA findings have been the subject of recent “candidate-miRNA” investigations. Specifically, the expression levels of our top hit, hsa-miR-1244, have been shown to decrease in A549 and NCI-H522 cells, as well as patient-derived tumor samples after cisplatin treatment, with the overall survival times of cisplatin-treated patients being longer for those who had high miR-1244 expression^37^. Further, overexpression of miR-1244 suppressed cell viability and increased apoptosis in the standard NSCLC cell lines by promoting caspase-3 activity, p53 and Bax protein expression, and suppressing myocyte enhancer factor 2D (MEF2D) and cyclin D1 protein expression. In a separate study^2^, miR-1244 was again associated with cisplatin efficacy, as both miR-1244 and miR-589 were significantly downregulated in cisplatin-resistant A549 cells (A549/DDP) when compared to the parental cell line, and transfection of A549/DDP with either miRNA markedly increased sensitivity to cisplatin, indicating that miR-1244 has important tumor suppressive functions that may be vital in the treatment of NSCLC.

Interestingly, the second highest differentially expressed miRNA in our study, hsa-miR-494, has also been associated with NSCLC cell survival, but contrary to miR-1244, the miRNA was demonstrated to have carcinogenic potential^38^. Not only was miR-494 the most downregulated miRNA when oncogenic ERK1/2 signaling was blocked, but its upregulation potentiated TNF-related apoptosis-inducing ligand (TRAIL) resistance via inhibition of pro-apoptotic Bim (Bcl-2-like protein 11), a protein known to be suppressed in NSCLC resistant to several antineoplastic agents^39–42^. Notably, a common *BIM* deletion has been shown to confer intrinsic resistance to tyrosine kinase inhibitors in NSCLC cell lines and to be associated with shorter progression-free survival in EGFR-driven NSCLC^42^. Taken together, these data suggest that miRNAs influence cancer cell survival via both oncogenic and tumor suppressive mechanisms, and that the levels of these non-coding RNAs are substantially altered after cells are exposed to stressful conditions, such as cytotoxic insult.

There have been a limited number of studies evaluating global miRNA expression changes in response to cytotoxic insult, but several intriguing findings have been previously noted. For example, in the human breast carcinoma cell line MCF7 (luminal A molecular subtype), 5-FU was found to significantly alter the global expression profile of miRNA with 42 out of 871 human miRNAs differentially expressed^43^. In addition, doxorubicin has shown a similar effect on altering miRNA expression in breast cancer cell lines, which has also been demonstrated in MCF7 cells^44^, as well as in a separate study that examined malignancies of both luminal A and triple negative molecular subtype^45^. Further, a global assessment of CaSki and HeLa cervical carcinoma cell lines revealed 9 miRNAs showing altered expression in response to cisplatin treatment^46^. Using two human esophageal carcinoma cell lines (KYSE410 squamous cell carcinoma and OE19 adenocarcinoma), a genome wide study also revealed that levels for a total of 13 miRNAs were altered after cisplatin or 5-FU treatment (24 or 72 hours), with further pathway analyses suggesting that miRNAs might play important roles in cellular response to chemotherapeutic agents via interactions with cell survival pathways^47^. Interestingly, a recent clinical study demonstrated that decreases in circulating levels of miRNA-126 following administration of chemotherapy (capecitabine; XELOX) and bevacizumab in patients with metastatic colorectal carcinoma was associated with a better response^48^, indicating that it may be possible to use miRNA expression as a predictive biomarker for treatment outcome.

The importance of miRNAs in determining response to pemetrexed treatment is an important finding since miRNAs are known to regulate a large number of genes in the human genome. Our study suggests the value of a systems biology approach on identifying chemotherapeutic-activated gene networks. Consistent with this insight, we found that the mRNAs with significant expression changes after pemetrexed treatment clustered into biological networks. Indeed, we observed a significant number of already known *in vitro* associations between these genes, and also found the genes to be more densely connected than expected by chance. The high degree of network connectivity among the most differentially perturbed genes after drug exposure suggests that such interaction analyses may be used to implicate other genes affected by pemetrexed treatment. Notably, the differentially expressed genes most highly associated with each other were enriched for the proteasome, a protein complex important for protein degradation. This is particularly noteworthy given recent findings on the use of the proteasome inhibitor bortezomib in combination with pemetrexed in malignant pleural mesothelioma, in which bortezomib was found to increase the cytotoxicity of pemetrexed in a concentration-dependent manner^49^. However, it should be noted that the two agents demonstrated potentially antagonistic activity when used in combination against H460 and H1299 human NSCLC cells *in vitro*^50^. Further, the concomitant administration of bortezomib and pemetrexed elicited no additional response or survival benefit to NSCLC patients when compared to pemetrexed alone in a phase II study, with bortezomib alone showing no clinically significant activity^51^. Although these negative findings do not preclude further investigation of this drug combination against NSCLC, it may be worthwhile to focus investigational efforts on other pemetrexed based treatment strategies that have not been comprehensively evaluated at the clinical level.

The differentially expressed genes following pemetrexed treatment implicated certain molecular processes, including acetylation (the most significantly altered). Histone acetylation/deacetylation is an essential aspect of gene regulation, and the inhibition of histone deacetylases (HDACs) has been recently shown to augment the antineoplastic effects of pemetrexed against multiple NSCLC cell lines *in vitro* and in patient-derived xenograft mouse models *in vivo* through induction of apoptosis and autophagy^52^. The ability of HDAC inhibitors to sensitize neoplastic cells to pemetrexed has also been demonstrated *in vivo* against mesothelioma^53^, providing a strong rationale to further investigate this drug combination in tumors that respond to pemetrexed treatment in the clinic. Furthermore, there was a significant enrichment of *phosphoproteins*. Phosphodiesterase inhibitors have been shown to enhance the effects of pemetrexed in several NSCLC cell lines including A549 cells^54^.

The present study indicates that although pemetrexed is expected to exert its antineoplastic activity by inhibiting folate-dependent enzymes, other pathways and their corresponding genes are crucially affected via protein-protein interaction and biological networks. Specifically, our data on the importance of *MTHFD2*, histone acetylation, and the proteasome in modulating pemetrexed response indicate that combination therapies involving miRNAs/small molecule inhibitors, HDAC inhibitors, or proteasome inhibitors, respectively should be further evaluated as approaches to enhancing the cytotoxicity of pemetrexed. The role of miRNAs in potentiating the resistance of A549 to pemetrexed highlights the importance of these noncoding RNAs in influencing response to pemetrexed. These data also highlight the utility of an integrative genomic approach to identify drug-induced changes in the transcriptome that implicate functionally related biological networks, and investigating this pharmacogenomics strategy for other cytotoxic agents and types of malignancy is warranted.

## Methods

### Cell lines and drugs

Previously, we evaluated the CEU phase I/II LCLs (84 total) for sensitivity to pemetrexed after 72 hours using alamarBlue (a short-term, colorimetric growth inhibition assay)^8^. These LCLs were derived from (unrelated) Utah residents of Northern and Western European ancestry (Coriell Institute for Medical Research; Camden, NJ). The percent survival was evaluated at increasing concentrations of pemetrexed for each cell line and area under the curve was determined. A total of 11 LCLs were next chosen as the most sensitive or resistant for further evaluation (Supplemental Fig. 1). Five of the cell lines (GM07055, GM11994, GM11995, GM12813, and GM12762) were more sensitive to pemetrexed (0.5 µM), defined by having 41–57% cell survival following treatment. LCLs that were more resistant to pemetrexed were defined by 87–105% cell survival following the same treatment, and included GM06994, GM07022, GM07034, GM12044, GM12144, and GM12873.

LCLs were grown in RPMI 1640 media (Mediatech; Herndon, VA) supplemented with 15% fetal bovine serum (HyClone; Logan, UT) and 2% L-glutamine (Mediatech) and were maintained per manufacturer’s protocol. 10,000 LCLs/well were plated in 100 µL media overnight in Costar 96-well dishes (Corning; Lowell, MA) then treated with 0.5 µM pemetrexed or vehicle (PBS). Change in cell viability was determined by adding alamarBlue (ThermoFisher Scientific; Valencia, CA) at 48 hours and reading the output the next day on the HT Synergy plate reader (BioTek Instruments; Winooski, VT) at 72 hours drug treatment. The identities of the LCLs from Coriell were confirmed by random checks several times per year by genotyping the 47 informative SNPs included in the Sequenom iPLEX Sample ID Plus Panel (San Diego, CA) as previously described^55^.

A549, a NSCLC cell line (CCL-185), was purchased from the American Type Culture Collection (Manassas, VA) and maintained in F-12K growth media (ThermoFisher) containing 10% FBS (HyClone) and 1% Pen/Strep (Corning). 5000 A549 cells/well were plated in 100 µL media overnight in Costar 96-well dishes (Corning). The following day, the media was exchanged with 10 or 100 µM pemetrexed or vehicle (PBS). After 6 days, cell viability was measured with the addition of equal amount CellTiter-Glo (Promega,Corp.; Madison, WI) and luminescence recorded as per manufactures directions. Authentication of the A549 cell line was performed twice during the study by IDEXX BioResearch (Columbia, MO) to check for interspecies contamination and proper identification. This authentication was conducted by measuring short tandem repeat (STR) using the Promega cell check 9-human CELL ID System (Madison, WI)

Pemetrexed disodium (CAS: 150399–23–8) was a gift from the Eli Lilly Corporation (Indianapolis, IN) and dissolved fresh in PBS immediately before each drug treatment.

### mRNA and miRNA expression profiling in LCLs

For each LCL, cells were plated at 1 × 10^5^ per mL in T75 flasks and 20 hours later treated with 0.5 µM pemetrexed or vehicle (PBS) for 24 hours before being pelleted (400xg at 4 °C and rinsed twice with ice cold PBS). To evaluate three biological replicates for each LCL, the cells were independently thawed, treated with pemetrexed or vehicle and pelleted, resulting in a total of 66 pellets (11 LCLs, 3 biological replicates, 2 treatments). Whole RNA was extracted using Qiagen miRNeasy kits (Germantown, MD) and treated with DNase1. RNA quality was assessed using the RNA 6000 Nano assay (Agilent Technologies; Santa Clara, CA). All samples had an RNA Integrity Number of 10. We used the Affymetrix GeneChip Human Gene 1.0 ST Array and Affymetrix GeneChip miRNA 2.0 Array to assess drug-induced changes in mRNA and miRNA, respectively.

### Differential expression following drug exposure

For the mRNAs and miRNAs interrogated on their respective arrays, the raw signal intensity estimates encoded in the CEL files (each of which contains probe-level intensities from each of 3 replicates for a given sample – pemetrexed treated or untreated) were processed using the *apt* suite of tools. All raw mRNA signal intensity data were processed in one batch, as were all miRNA signal intensity data. Gene-level and miRNA-level summaries were generated for each sample using the mean of the 3 corresponding replicates. To identify mRNAs and miRNAs differentially expressed between the pemetrexed treated and untreated samples, we performed Linear Models for Microarray Data (*limma*)^56^ analysis for the paired samples (i.e., pemetrexed treated and untreated). The log-fold change (logFC) of the quantified expression values for treated relative to untreated samples and the log-odds (*B*-statistic) for differential expression were calculated for each probe using the *limma* package as implemented in the Bioconductor project. The test statistic used for inference about a gene *j* was the “moderated” *t*-statistic^56^, where the posterior variance takes the place of the usual variance in the definition of the classical *t*-statistic:

1 $${\tilde{s}}_{j}^{2}=\frac{{d}_{0}{s}_{0}^{2}+{d}_{j}{s}_{j}^{2}}{{d}_{0}+{d}_{j}}$$

The approach moderates the variance estimates using a common value from a Bayesian model, thereby reducing false positives due to underestimation of the variance. The Bayesian model uses an inverse chi-squared prior for the unknown variance ${\sigma }_{j}^{2}\,$with mean ${s}_{0}^{2}$ and degrees of freedom ${d}_{0}$:

2 $$\frac{1}{{\sigma }_{j}^{2}} \sim (\frac{1}{{d}_{0}{s}_{0}^{2}}){\chi }_{{d}_{0}}^{2}$$

The moderated *t*-statistic has increased degrees of freedom, ${d}_{0}+{d}_{j}$ (with ${d}_{j}$ equal to the residual degrees of freedom for the *j*th gene and ${d}_{0}$ representing the additional degrees of freedom from the parallel structure of the entire gene set), relative to the ordinary *t*-statistic. The comparisons between the p-values obtained from the ordinary *t*-test and from the *limma* analysis for the mRNAs and miRNAs (each RNA type evaluated separately) are shown in Supplemental Figures 8 and 9, respectively. All differential expression results reported in our study were from the *limma* analysis unless otherwise stated. We refer to an mRNA or miRNA with BH adjusted p-value < 0.05 as “differentially expressed” between the two conditions; all differential expression analyses were conducted using this multiple testing adjustment. Nonetheless, we also report those mRNAs and miRNAs that pass stringent Bonferroni correction (p < 0.05/number of statistical tests, with the number of tests equal to 22,245 for mRNAs and 1,110 for miRNAs.).

All statistical analyses were performed using R (http://www.r-project.org). The heatmaps for the differential expression analyses were generated using the heatmap.2 R function. We generated a dendrogram and obtained an “Approximately Unbiased” p-value and a “Bootstrap Probability” value for each cluster from multiscale bootstrap resampling, as implemented in the pvclust library, to assess the uncertainty in the clustering results. We used “average” as the agglomerative method for hierarchical clustering and “correlation” as the distance method. We assumed 1000 bootstrap replicates and identified the clusters for which the null hypothesis that the cluster does not exist is rejected at the significance level of 0.05.

### Genetic regulation of differentially expressed mRNAs

To identify potential genetic mechanisms underlying the differential expression (mRNA) findings reported here, we evaluated publicly available expression quantitative trait loci (eQTLs) data in human lung tissue samples derived from 278 individuals as part of the Genotype-Tissue Expression (GTEx) project^10,11^ v6p release^12^. RNA sequencing had been performed on these samples by the GTEx consortium. We report all *cis*-acting eQTLs (defined as within 1 Mb of the target gene) identified for the differentially expressed mRNAs.

### Functional enrichment analysis of the differentially expressed mRNAs

Using DAVID^57^, we performed Gene Ontology (GO) analysis on the top 250 differentially expressed genes to identify shared pathways and annotations. Enriched annotations (Bonferroni-adjusted p < 0.05) were identified.

### Protein-protein interaction analysis

Assuming 1,000 within-degree node-label permutations, we reconstructed a protein-protein interaction (PPI) network from the differentially expressed genes using the Disease Association Protein-Protein Link Evaluator (DAPPLE) approach^13^. Proteins were represented as nodes and direct interactions as edges in the network. In this analysis, an edge between two proteins indicates that a direct interaction from a resource of high-confidence *in vitro* direct interactions, as cataloged in InWeb^58^, was identified. We also considered the mean indirect degree (i.e., the average number of proteins with which a seed protein indirectly interacts, where an “indirect” interaction is induced by a protein not on the input list of differentially expressed genes but which interacts with two or more seed proteins) to test for enrichment and evaluated the significance as the proportion of permutations with mean indirect degree that matched or exceeded the observed value. We plotted the permutation null distribution using the R package ggplot2.

### mRNA replication using an independent microarray experiment

We conducted additional replication studies on the results of the differentially expressed mRNAs with inverse correlation with differentially expressed miRNAs. Using *limma*, we re-analyzed microarray-based (Affymetrix Human Gene 1.0 ST) whole-genome gene expression profiling^14^ of pemetrexed treated EA.hy 926 cells^59^, a fusion of human umbilical vascular endothelial cells and A549, to evaluate changes in gene expression in cells grown under low or high folate conditions.

### mRNA expression in A549 cells

A549 were grown for 24 hours before being treated with 10 or 100 µM pemetrexed or vehicle for 24, 48, or 72 hours. Cell pellets were collected, and qPCR performed for PMAIP1 and MTHFD2 as previously described^22^. Results were analyzed using the ΔΔCT method and expressed as fold change in expression (10^(ΔΔCT/m)^; where m = average slope of the two genes determined by a standard curve).

### Apoptosis and survival in pemetrexed treated A549 cells

A549 cells were plated as described above and treated at 24 hours with 10 or 100 µM pemetrexed or vehicle. After 72 hours, the cells were assayed for apoptosis and viability using the Promega Caspase 3/7-Glo and CellTiter-Glo kits per the manufacturer’s protocols.

### Survival and molecular profiling analysis using The Cancer Genome Atlas

TCGA^60^ was used to evaluate somatic mutation spectra, copy number alterations and molecular profiles (including mRNA) of 230 lung adenocarcinoma patients. For those genes that were differentially expressed in both LCLs and EA.hy 926 cells after pemetrexed exposure, we considered the proportion of TCGA cases in which the gene was found to be altered through amplification, mutation, or mRNA upregulation using cBioPortal for Cancer Genomics^61^. Furthermore, we compared the median survival months for cases with and cases without such alterations. We also tested the top 20 differentially expressed mRNAs (Table 1) for association with survival time.

### Gene expression based imputation using LCL sensitivity to pemetrexed data

We have recently published a gene expression imputation approach^20^ that can be used for identifying trait-associated genes. Others^19^ have explored the utility of imputing drug response, with notable results, in cancer patients using cell-based models to enable discovery of pharmacogenomic biomarkers. Here we developed a gene expression based imputation model of drug response (Supplemental Figure 10) from drug (pemetrexed) sensitivity and mRNA data on the cell lines (LCL). We applied the model to TCGA lung adenocarcinoma expression data to impute drug response in these samples and to correlate this imputed trait with survival time. To develop the model, we used penalized regression (glmnet, α = 1)^62^ to extract the *p*-vector β of effect sizes that solves the following minimization problem:

3 $$\mathop{\min }\limits_{{\beta }_{0},\,\beta }\frac{{\sum }_{k=1}^{n}{({y}_{k}-{\beta }_{0}-{g}_{k}^{T}\beta )}^{2}}{n}$$

subject to the constraint on the ${L}_{1}$ norm of $\beta$, $\sum _{j=1}^{p}|{\beta }_{j}|\le t$. Here ${y}_{k}$ is the outcome (sensitive or resistant) for the sample *k* (among the *n* samples) and *p* is the number of genes. This can be expressed using the Lagrangian formulation, which introduces a new parameter λ. We assumed 3-fold cross validation in the model building. One can develop an imputation model using the same algorithm applied to other molecular traits (e.g., the miRNAs), but we focused on the mRNA dataset here because of the greater availability in cancer datasets.

## Electronic supplementary material

Supplementary Material

## References

[1] Chan BA, Hughes BG (2015). Targeted therapy for non-small cell lung cancer: current standards and the promise of the future. Transl Lung Cancer Res.

[2] Dammeijer F (2016). Efficacy of Tumor Vaccines and Cellular Immunotherapies in Non-Small-Cell Lung Cancer: A Systematic Review and Meta-Analysis. J Clin Oncol.

[3] Bibby AC (2016). Malignant pleural mesothelioma: an update on investigation, diagnosis and treatment. Eur Respir Rev.

[4] Genova C (2013). Pemetrexed for the treatment of non-small cell lung cancer. Expert Opin Pharmacother.

[5] Grosso F, Scagliotti GV (2012). Systemic treatment of malignant pleural mesothelioma. Future Oncol.

[6] Gerber DE, Schiller JH (2013). Maintenance chemotherapy for advanced non-small-cell lung cancer: new life for an old idea. J Clin Oncol.

[7] Garofalo M, Croce CM (2013). MicroRNAs as therapeutic targets in chemoresistance. Drug Resist Updat.

[8] Hong L, Yang Z, Ma J, Fan D (2013). Function of miRNA in controlling drug resistance of human cancers. Curr Drug Targets.

[9] Gamazon ER (2010). Exprtarget: an integrative approach to predicting human microRNA targets. PloS one.

[10] Consortium GT (2013). The Genotype-Tissue Expression (GTEx) project. Nature genetics.

[11] Consortium GT (2015). Human genomics. The Genotype-Tissue Expression (GTEx) pilot analysis: multitissue gene regulation in humans. Science.

[12] Consortium GT (2017). Genetic effects on gene expression across human tissues. Nature.

[13] Rossin EJ (2011). Proteins encoded in genomic regions associated with immune-mediated disease physically interact and suggest underlying biology. PLoS Genet.

[14] Hammons AL (2012). Pemetrexed alters folate phenotype and inflammatory profile in EA.hy 926 cells grown under low-folate conditions. Eur J Pharmacol.

[15] Oda E (2000). Noxa, a BH3-only member of the Bcl-2 family and candidate mediator of p53-induced apoptosis. Science.

[16] Lowman XH (2010). The proapoptotic function of Noxa in human leukemia cells is regulated by the kinase Cdk5 and by glucose. Mol Cell.

[17] Brodska B, Otevrelova P, Holoubek A (2011). Decitabine-induced apoptosis is derived by Puma and Noxa induction in chronic myeloid leukemia cell line as well as in PBL and is potentiated by SAHA. Mol Cell Biochem.

[18] Vazquez A, Tedeschi PM, Bertino JR (2013). Overexpression of the mitochondrial folate and glycine-serine pathway: a new determinant of methotrexate selectivity in tumors. Cancer Res.

[19] Geeleher P (2017). Discovering novel pharmacogenomic biomarkers by imputing drug response in cancer patients from large genomics studies. Genome research.

[20] Gamazon ER (2015). A gene-based association method for mapping traits using reference transcriptome data. Nature genetics.

[21] Geeleher P, Gamazon ER, Seoighe C, Cox NJ, Huang RS (2016). Consistency in large pharmacogenomic studies. Nature.

[22] Wen Y (2012). An eQTL-based method identifies CTTN and ZMAT3 as pemetrexed susceptibility markers. Human molecular genetics.

[23] HapMap C (2003). The International HapMap Project. Nature.

[24] Pastinen T (2005). Mapping common regulatory variants to human haplotypes. Hum Mol Genet.

[25] Duan S (2008). Genetic architecture of transcript-level variation in humans. Am J Hum Genet.

[26] Spielman RS (2007). Common genetic variants account for differences in gene expression among ethnic groups. Nat Genet.

[27] Wheeler HE, Dolan ME (2012). Lymphoblastoid cell lines in pharmacogenomic discovery and clinical translation. Pharmacogenomics.

[28] Welsh M (2009). Pharmacogenomic discovery using cell-based models. Pharmacol Rev.

[29] Gustafsson R (2017). Crystal Structure of the Emerging Cancer Target MTHFD2 in Complex with a Substrate-Based Inhibitor. Cancer Res.

[30] Nilsson R (2014). Metabolic enzyme expression highlights a key role for MTHFD2 and the mitochondrial folate pathway in cancer. Nat Commun.

[31] Yang M, Vousden KH (2016). Serine and one-carbon metabolism in cancer. Nat Rev Cancer.

[32] Goricar K, Kovac V, Dolzan V (2014). Polymorphisms in folate pathway and pemetrexed treatment outcome in patients with malignant pleural mesothelioma. Radiol Oncol.

[33] Moran DM (2014). KRAS mutation status is associated with enhanced dependency on folate metabolism pathways in non-small cell lung cancer cells. Mol Cancer Ther.

[34] Indran IR, Tufo G, Pervaiz S, Brenner C (2011). Recent advances in apoptosis, mitochondria and drug resistance in cancer cells. Biochim Biophys Acta.

[35] Bareford MD (2011). Sorafenib enhances pemetrexed cytotoxicity through an autophagy-dependent mechanism in cancer cells. Cancer Res.

[36] Costantini P, Jacotot E, Decaudin D, Kroemer G (2000). Mitochondrion as a novel target of anticancer chemotherapy. J Natl Cancer Inst.

[37] Li GJ (2017). Effect of miR-1244 on cisplatin-treated non-small cell lung cancer via MEF2D expression. Oncology reports.

[38] Romano G (2012). MiR-494 is regulated by ERK1/2 and modulates TRAIL-induced apoptosis in non-small-cell lung cancer through BIM down-regulation. Proceedings of the National Academy of Sciences of the United States of America.

[39] Li R, Moudgil T, Ross HJ, Hu HM (2005). Apoptosis of non-small-cell lung cancer cell lines after paclitaxel treatment involves the BH3-only proapoptotic protein Bim. Cell death and differentiation.

[40] Lee JY (2015). The BIM Deletion Polymorphism and its Clinical Implication in Patients with EGFR-Mutant Non-Small-Cell Lung Cancer Treated with EGFR Tyrosine Kinase Inhibitors. Journal of thoracic oncology: official publication of the International Association for the Study of Lung Cancer.

[41] Nie W, Tao X, Wei H, Chen WS, Li B (2015). The BIM deletion polymorphism is a prognostic biomarker of EGFR-TKIs response in NSCLC: A systematic review and meta-analysis. Oncotarget.

[42] Ng KP (2012). A common BIM deletion polymorphism mediates intrinsic resistance and inferior responses to tyrosine kinase inhibitors in cancer. Nature medicine.

[43] Shah MY, Pan X, Fix LN, Farwell MA, Zhang B (2011). 5-Fluorouracil drug alters the microRNA expression profiles in MCF-7 breast cancer cells. J Cell Physiol.

[44] Lv J (2014). miRNA expression patterns in chemoresistant breast cancer tissues. Biomed Pharmacother.

[45] Tormo E (2015). MicroRNA Profile in Response to Doxorubicin Treatment in Breast Cancer. J Cell Biochem.

[46] Phuah NH (2013). Alterations of microRNA expression patterns in human cervical carcinoma cells (Ca Ski) toward 1′S-1′-acetoxychavicol acetate and cisplatin. Reprod Sci.

[47] Hummel R (2011). Chemotherapy-induced modification of microRNA expression in esophageal cancer. Oncol Rep.

[48] Hansen TF, Carlsen AL, Heegaard NH, Sorensen FB, Jakobsen A (2015). Changes in circulating microRNA-126 during treatment with chemotherapy and bevacizumab predicts treatment response in patients with metastatic colorectal cancer. Br J Cancer.

[49] Gordon GJ (2008). Preclinical studies of the proteasome inhibitor bortezomib in malignant pleural mesothelioma. Cancer Chemother Pharmacol.

[50] Neukirchen J (2007). The proteasome inhibitor bortezomib acts differently in combination with p53 gene transfer or cytotoxic chemotherapy on NSCLC cells. Cancer Gene Ther.

[51] Scagliotti GV (2010). A randomized phase II study of bortezomib and pemetrexed, in combination or alone, in patients with previously treated advanced non-small-cell lung cancer. Lung Cancer.

[52] Del Bufalo D (2014). Histone deacetylase inhibition synergistically enhances pemetrexed cytotoxicity through induction of apoptosis and autophagy in non-small cell lung cancer. Mol Cancer.

[53] Vandermeers F (2009). Valproate, in combination with pemetrexed and cisplatin, provides additional efficacy to the treatment of malignant mesothelioma. Clin Cancer Res.

[54] Booth L, Roberts JL, Poklepovic A, Gordon S, Dent P (2017). PDE5 inhibitors enhance the lethality of pemetrexed through inhibition of multiple chaperone proteins and via the actions of cyclic GMP and nitric oxide. Oncotarget.

[55] Komatsu M (2015). Pharmacoethnicity in Paclitaxel-Induced Sensory Peripheral Neuropathy. Clin Cancer Res.

[56] Ritchie ME (2015). limma powers differential expression analyses for RNA-sequencing and microarray studies. Nucleic Acids Res.

[57] Dennis G (2003). DAVID: Database for Annotation, Visualization, and Integrated Discovery. Genome Biol.

[58] Lage K (2007). A human phenome-interactome network of protein complexes implicated in genetic disorders. Nat Biotechnol.

[59] Edgell CJ, McDonald CC, Graham JB (1983). Permanent cell line expressing human factor VIII-related antigen established by hybridization. Proc Natl Acad Sci USA.

[60] Wheeler HE (2014). Poly-omic prediction of complex traits: OmicKriging. Genetic epidemiology.

[61] Cerami E (2012). The cBio cancer genomics portal: an open platform for exploring multidimensional cancer genomics data. Cancer Discov.

[62] Hastie, T., Tibshirani, R. & Wainwright, M. Statistical learning with sparsity: the lasso and generalizations. CRC press, 2015.

