# Supplementary material for: Gene and MicroRNA Perturbations of Cellular Response to Pemetrexed Implicate Biological Networks and Enable Imputation of Response in Lung Adenocarcinoma
Source: Sci Rep. 2018 Jan 15;8:733. doi: 10.1038/s41598-017-19004-3 (PMC5768793; doi:10.1038/s41598-017-19004-3)
Supplement: Supplementary file 1 — Supplementary Material [file 41598_2017_19004_MOESM1_ESM.pdf]

Supplemental Materials to:

Gene and MicroRNA Perturbations of Cellular Response to Pemetrexed Implicate Biological Networks and Enable Imputation of Response in Lung Adenocarcinoma

Eric R. Gamazon[2,#,*], Matthew R. Trendowski[1], Yujia Wen[1], Claudia Wing[1], Shannon M Delaney[1],Won Huh[1], Shan Wong[1], Nancy J Cox[2,#], M. Eileen Dolan[1*]

[1]Section of Hematology/Oncology, [2]Section of Genetic Medicine, Department of Medicine, The University of Chicago, Chicago, IL 60637 USA

#Current address: Division of Genetic Medicine, Vanderbilt University School of Medicine, Nashville, TN 37232

Supplemental Figure 1

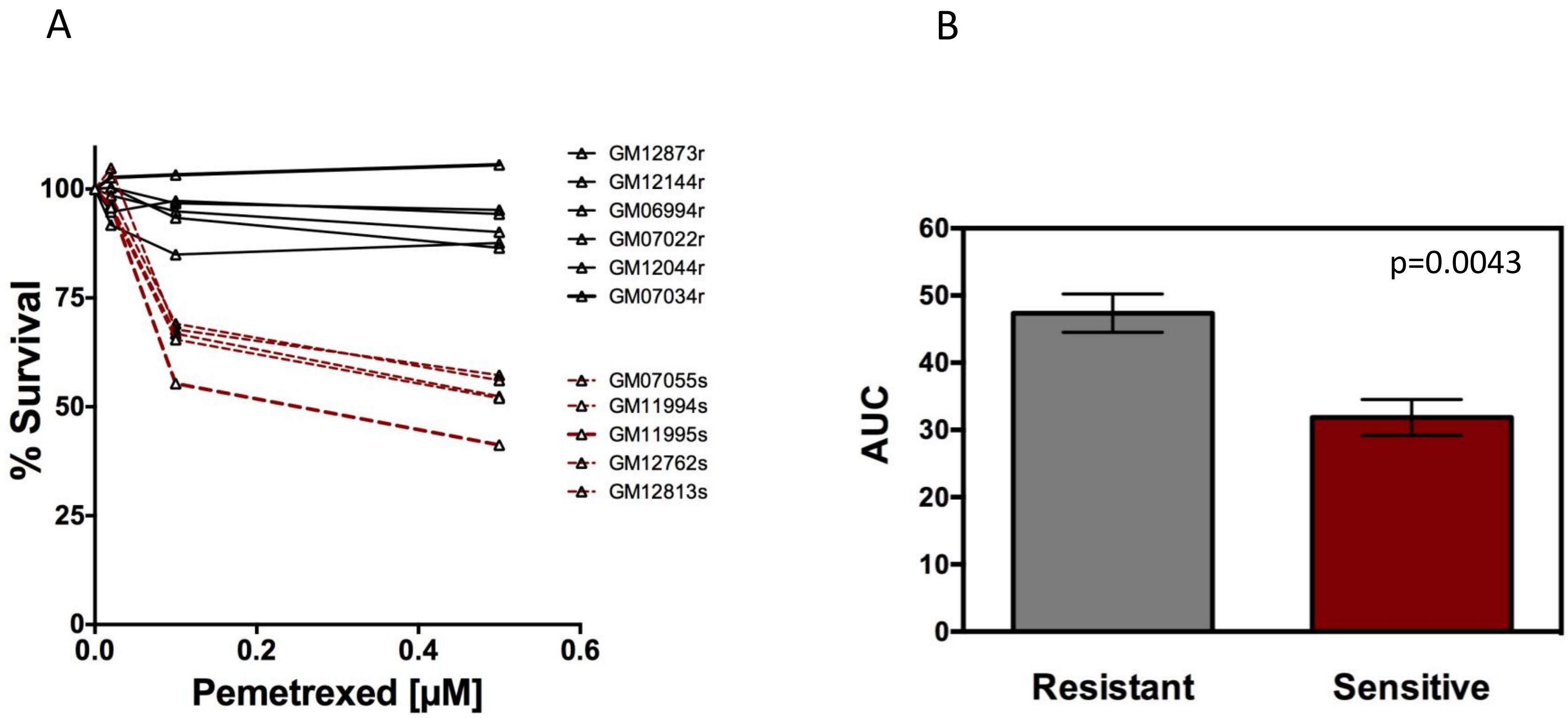

Supplemental Figure 1: Survival of the LCLs used in the study as measured by alamarBlue® after 72 hours pemetrexed treatment. (A) Mean survival from eleven LCLs derived from Utah residents of Northern and Western European ancestry (HapMap CEU phase I) representing the 5 most sensitive to pemetrexed (GM07055, GM11994, GM11995, GM12813 and GM12762) and 6 most resistant (GM07022, GM06994, GM07034, GM12044, GM12144 and GM12873) to pemetrexed. (B) The AUC was significantly different between the resistant compared to sensitive LCLs as determined by unpaired t-test and Mann Whitney assumptions ($p=0.0043$). Each LCL was tested in 3 independent experiments.

Supplemental Figure 2

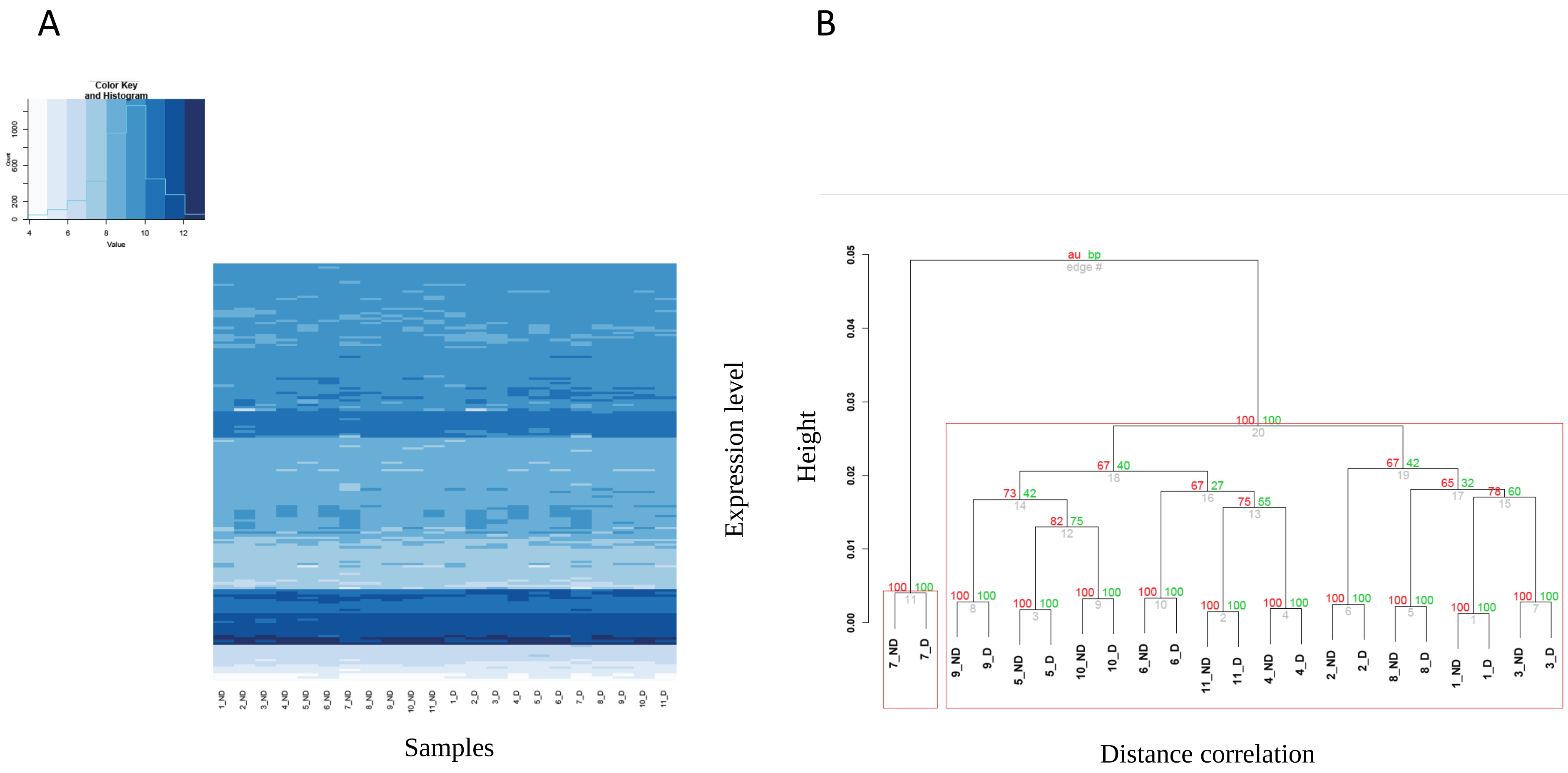

Supplemental Figure 2: (A) A heatmap of the differentially expressed mRNAs (BH adjusted p < 0.05) between treated and untreated LCLs. The order of cell lines for 1_ND-11_ND and 1_D-11_D: GM06994, GM07022, GM07034, GM07055, GM11994, GM11995, GM12044, GM12144, GM12762, GM12813, GM12873. The rows are mRNAs and the columns are the cell lines with the untreated samples (1_ND-11_ND) listed first and the paired treated ones (1_D-11_D) next. (B) Hierarchical clustering. Stability of the clusters is quantified using multiscale bootstrap resampling.

Supplemental Figure 3

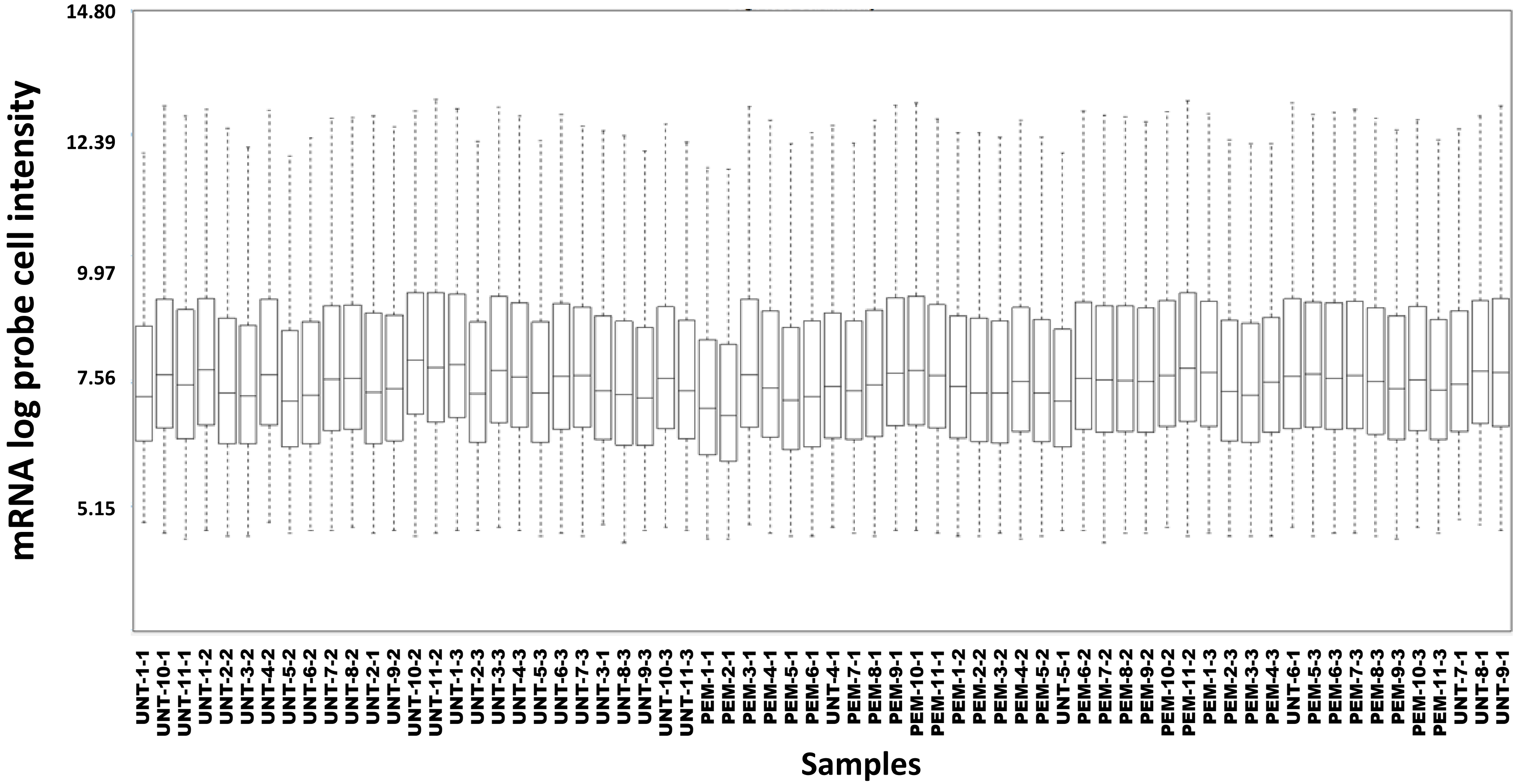

Supplemental Figure 3: Gene expression (mRNA) levels for all samples analyzed. Shown is a boxplot of the gene expression levels (log probe intensity) for the 3 replicates of each of the 22 samples (11 LCL samples treated either with or without pemetrexed). The cell lines are listed on the x-axis. UNT corresponds to no drug treatment, PEM corresponds to drug treatment, 1-11 corresponds to the LCLs (GM06994, GM07022, GM07034, GM07055, GM11994, GM11995, GM12044, GM12144, GM12762, GM12813, GM12873) and the final number refers to the replicate sample number 1, 2 or 3.

Supplemental Figure 4

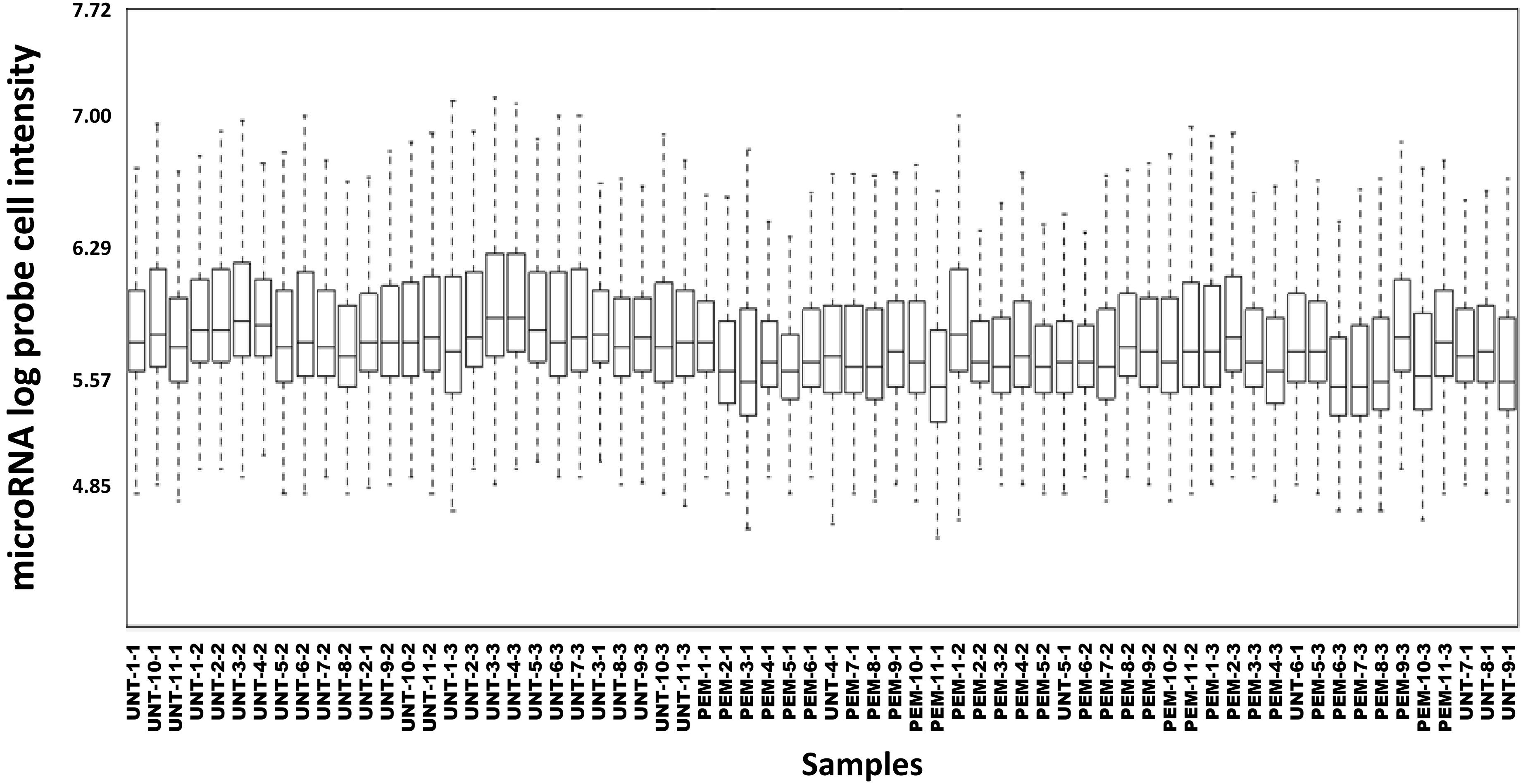

Supplemental Figure 4: Expression levels of miRNA for all samples analyzed. Shown is a boxplot of the miRNA levels (log probe intensity) for the 3 replicates of each of the 22 samples (11 LCL samples treated either with or without pemetrexed). The cell lines are listed on the x-axis. UNT corresponds to no drug treatment, PEM corresponds to drug treatment, 1-11 corresponds to the LCLs (GM06994, GM07022, GM07034, GM07055, GM11994, GM11995, GM12044, GM12144, GM12762, GM12813, GM12873) and the final number refers to the replicate sample number 1, 2 or 3.

Supplemental Figure 5

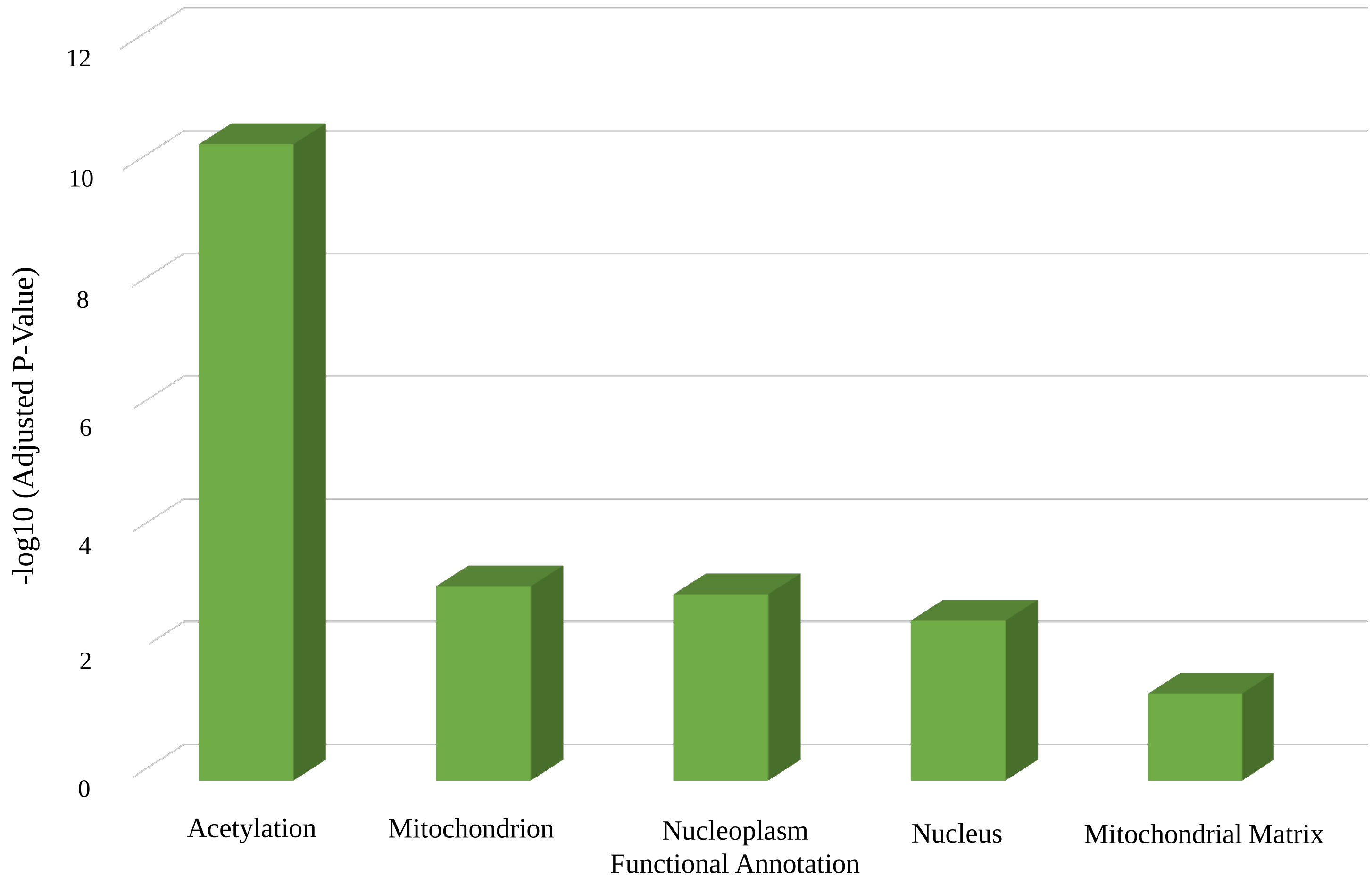

Supplemental Figure 5: Functional enrichment analysis of differentially expressed genes. Using DAVID, we looked for enriched functional annotations, such as Gene Ontology terms, among the most differentially expressed genes due to pemetrexed exposure. Enriched annotations (Bonferroni-adjusted $p < 0.05$) were identified.

Supplemental Figure 6

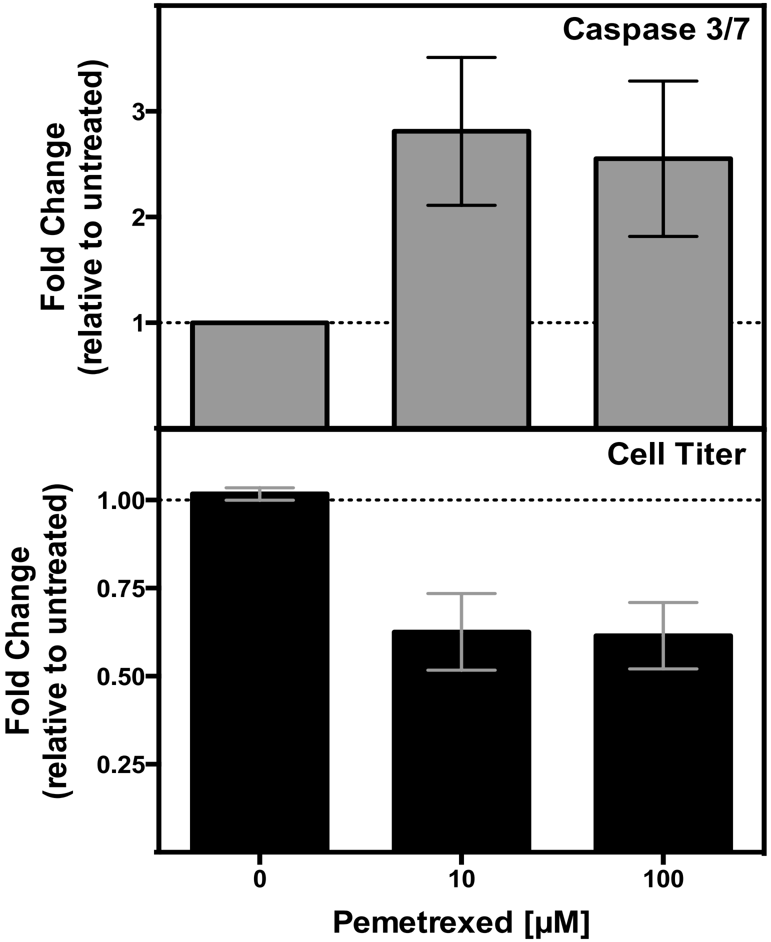

Supplemental Figure 6: Changes in survival of A549 cells treated with 0 , 10 μM or 100 μM pemetrexed for 72 hours. Cell survival declined significantly as measured by increased Caspase3/7 activation (t-test, **10 μM**: all p<0.001; **100 μM**: p<0.001, 0.03 and 0.003) in three independent experiments. CellTiter-Glo similarly reduced cell viability for both 10 and 100 μM pemetrexed (t-test, all p<0.001).

Supplemental Figure 7

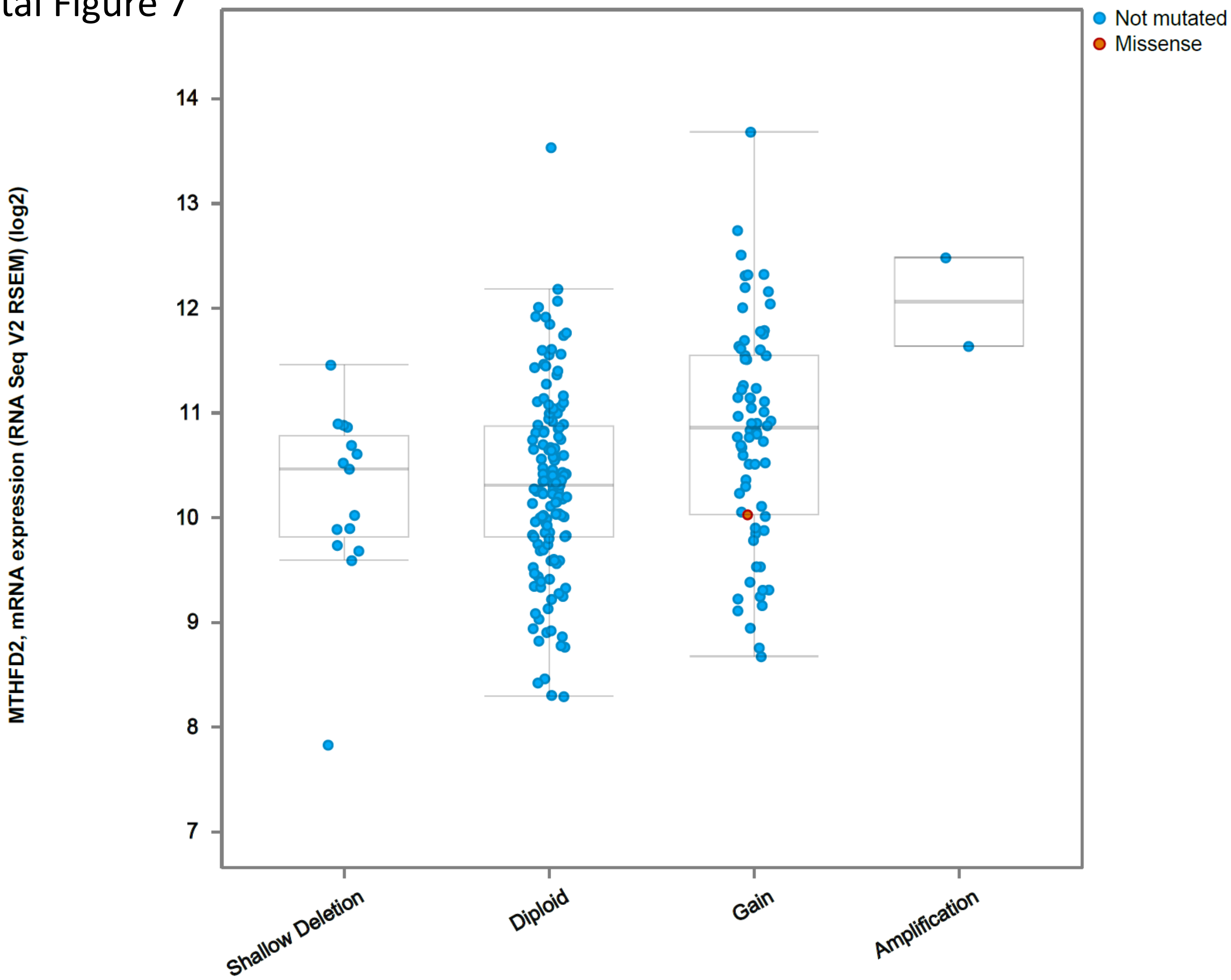

Supplemental Figure 7: mRNA upregulation of MTHFD2 as a result of copy number alteration. Each point is a patient from the TCGA lung adenocarcinoma dataset (N = 230 tumors). The plot was generated using cBioPortal.

Supplemental Figure 8

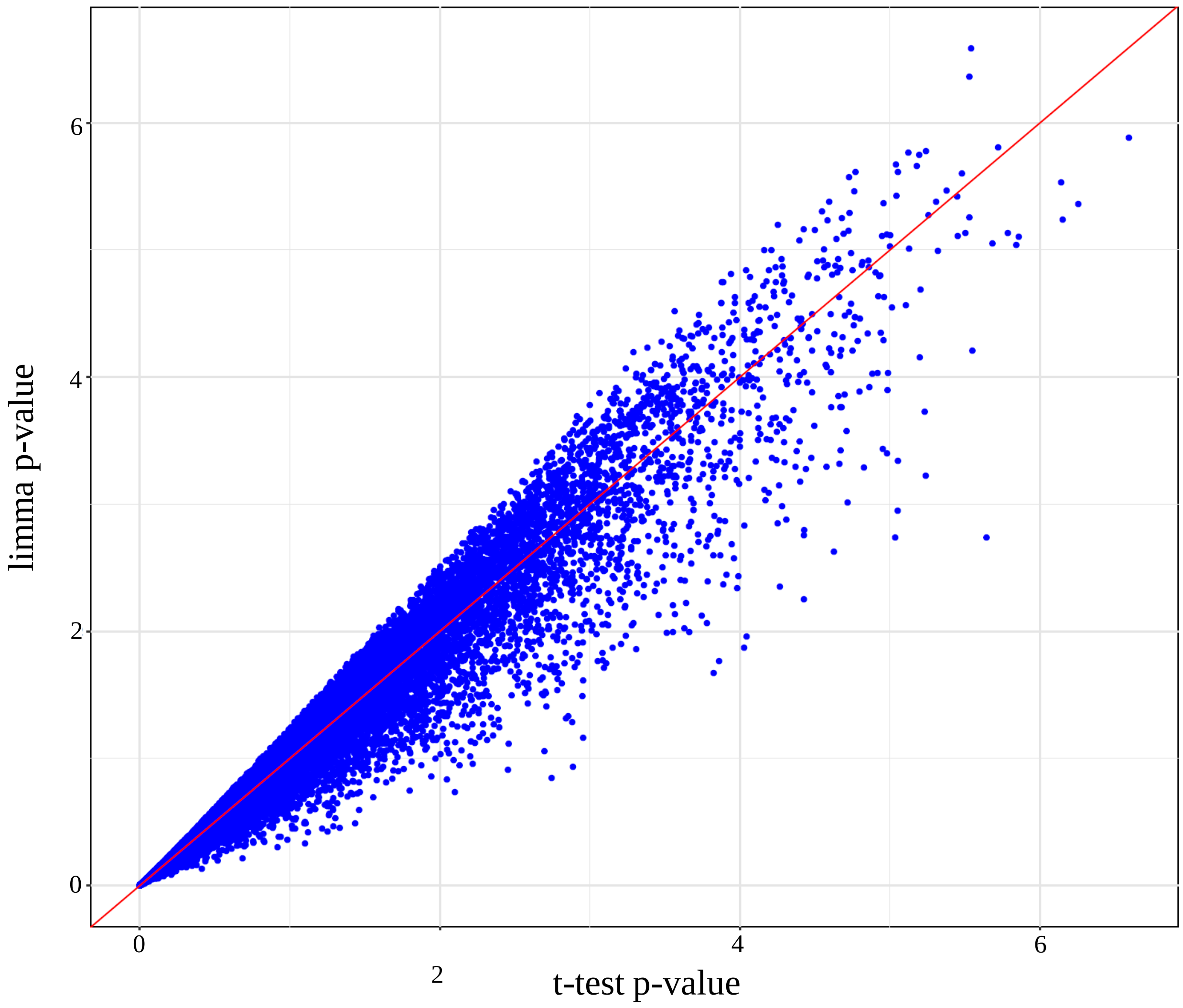

Supplemental Figure 8: A comparison of the (paired) t-test and limma p-values (in –log10 scale) for the entire set of mRNA probes.

Supplemental Figure 9

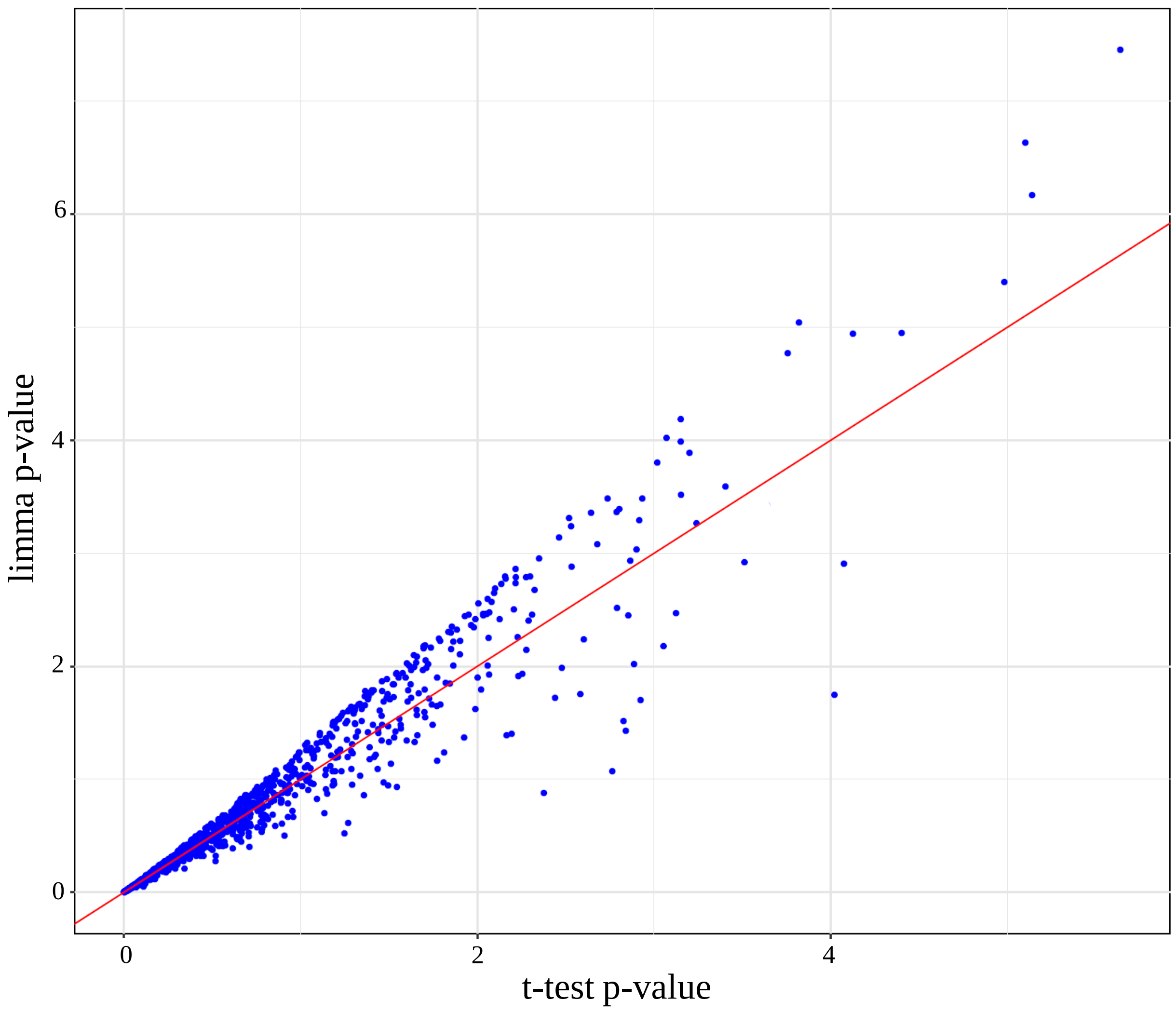

Supplemental Figure 9: A comparison of the (paired) t-test and limma p-values (in –log10 scale) for the entire set of miRNAs.

Supplemental Figure 10

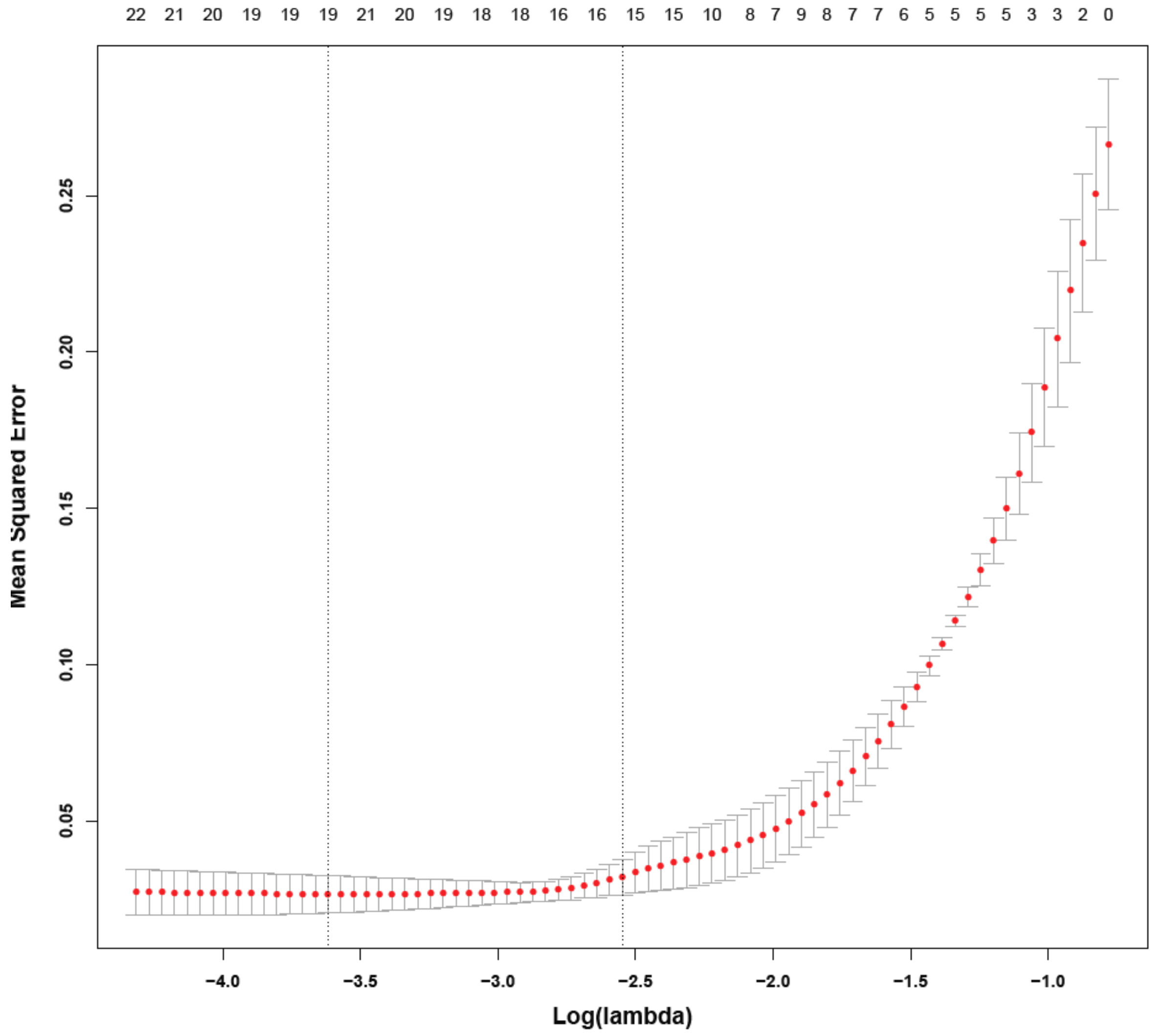

Supplemental Figure 10: Drug response imputation model based on mRNA data. We applied LASSO (glmnet) to build the model and extract a set of gene predictors of drug sensitivity. The red line indicates the cross-validation curve. The upper and lower standard deviation are also shown as error bars. The vertical lines are the values of lambda that give the minimum mean squared (cross-validation) error and the “lambda 1 SE” value.

Supplemental Table 1

Genes that were differentially expressed between PEM treated and untreated LCLs (BH adjusted p < 0.05). The top eight genes were statistically significant after Bonferroni adjustment, and are highlighted in bold.

logFC	p-value	B-statistic	gene
**0.211515**	**2.62E-07**	**7.061716**	**ZFAND1**
**-0.11212**	**1.31E-06**	**5.633543**	**LBR**
**0.127576**	**1.57E-06**	**5.470342**	**UCHL3**
**0.148485**	**1.67E-06**	**5.414052**	**FAM171B**
**0.156061**	**1.73E-06**	**5.384266**	**SFT2D1**
**0.148788**	**1.80E-06**	**5.348101**	**TMEM60**
**0.154242**	**2.13E-06**	**5.197697**	**C4orf33**
**0.142727**	**2.20E-06**	**5.164215**	**NDUFB5**
0.147576	2.44E-06	5.072225	LHFP
-0.17697	2.46E-06	5.06478	NARS
0.124848	2.52E-06	5.040896	ZNF426
0.178182	2.67E-06	4.990653	WBP4
0.106667	2.93E-06	4.904101	UCHL1
0.121212	3.44E-06	4.758466	CA5B
-0.15818	3.46E-06	4.752797	AHCTF1
-0.13333	3.75E-06	4.678756	BOP1
0.116667	3.81E-06	4.662621	MTFMT
0.168182	4.17E-06	4.580406	PSMC6
0.119091	4.17E-06	4.580266	C16orf80
-0.13364	4.32E-06	4.547733	TCEA1
-0.09939	4.39E-06	4.533022	NOP14
-0.16394	4.96E-06	4.419794	NBEAL1
0.14303	5.13E-06	4.390088	CCDC84
-0.11515	5.37E-06	4.346731	SRGAP2
0.107273	5.58E-06	4.311369	IAH1
0.143333	5.64E-06	4.302292	AKAP5
0.09697	5.77E-06	4.281624	PMAIP1
0.150303	5.89E-06	4.261097	AGA
-0.14152	6.82E-06	4.126171	SLC35E2
0.160303	6.93E-06	4.110772	ZMYM5
0.150606	6.99E-06	4.103739	ZNF880
0.131515	7.04E-06	4.096133	DZANK1///POLR3F
-0.10303	7.38E-06	4.05302	TRIM28
0.098182	7.42E-06	4.047389	TMEM14B
-0.13152	7.50E-06	4.038094	ARMC1
0.11697	7.54E-06	4.032857	TANK
0.115758	7.65E-06	4.019576	TMEM126B
-0.11727	7.82E-06	3.998774	EHD4
0.10303	7.83E-06	3.997502	STX2
0.096364	7.87E-06	3.992543	AAMP
-0.13152	8.21E-06	3.953474	SPOP

0.152424	8.45E-06	3.927019	LIAS
0.09697	8.95E-06	3.873583	RPAIN
0.094545	9.13E-06	3.854828	PSMB4
-0.11121	9.35E-06	3.832512	YWHAZ
-0.10636	9.86E-06	3.783316	SLC46A3
0.130303	9.91E-06	3.778227	C6orf162
0.179091	1.00E-05	3.767741	LACTB2
0.101212	1.02E-05	3.749272	42262
0.118182	1.06E-05	3.716062	BNIP2
-0.11909	1.18E-05	3.611754	LIMD1-AS1///LIMD1
0.147879	1.19E-05	3.609352	ZNF431
0.123636	1.22E-05	3.583841	CIR1
-0.11	1.22E-05	3.581855	LETM1
0.125758	1.23E-05	3.577038	STRADB
0.110909	1.25E-05	3.562101	ACOT9
-0.1197	1.31E-05	3.516789	SNORD15B
-0.1097	1.33E-05	3.50691	UPF1
0.116667	1.33E-05	3.501993	ETFA
0.140606	1.35E-05	3.492505	TXN
0.119697	1.36E-05	3.480323	ZBTB1
0.144848	1.37E-05	3.475893	TIMMDC1
0.107273	1.38E-05	3.471394	LOC100129518///ACAT2
-0.11394	1.40E-05	3.457944	C9orf129///FAM120A
0.109697	1.46E-05	3.418113	MTHFD2
-0.17152	1.46E-05	3.417774	PSMD5
0.103939	1.51E-05	3.382959	PRIM1
0.112727	1.51E-05	3.382823	SCPEP1
0.192121	1.55E-05	3.359749	GYPE///GYPA
-0.11333	1.56E-05	3.353161	FASN
0.120909	1.58E-05	3.341381	ZNF277
-0.10212	1.59E-05	3.336603	ELMO1
-0.13303	1.60E-05	3.328725	ZNF697
-0.10212	1.60E-05	3.32757	ARID2
0.120303	1.63E-05	3.312936	HSD17B11
-0.15727	1.64E-05	3.307461	HIC1
-0.11606	1.68E-05	3.284264	MED16
0.127879	1.77E-05	3.236437	POLD3
0.138182	1.78E-05	3.231551	ADPRM
0.131818	1.79E-05	3.225227	YEATS4
0.189697	1.79E-05	3.222547	RNASE6
-0.19303	1.80E-05	3.219685	HIST1H3J
0.126667	1.84E-05	3.196812	EAPP
-0.13636	1.93E-05	3.153824	TCEA1
0.092121	2.06E-05	3.092928	PARP15
0.121212	2.11E-05	3.072219	PRKRA
0.125758	2.14E-05	3.055228	LY86
0.115455	2.30E-05	2.988512	RFXAP

-0.13333	2.31E-05	2.984988	CCNF
-0.09545	2.32E-05	2.978724	KDM1B
0.122424	2.33E-05	2.97831	ZDHHC6
0.102121	2.34E-05	2.972999	RPS25
-0.09455	2.34E-05	2.972365	HSPA1B///HSPA1A
0.148182	2.36E-05	2.965101	ATP6V1D
0.11303	2.58E-05	2.879267	MLF1IP
-0.14212	2.61E-05	2.871005	CEBPA
0.131515	2.62E-05	2.864745	NUP54
-0.15515	2.63E-05	2.860826	HIST2H3D///HIST2H3A///HIST2H3C
-0.15515	2.63E-05	2.860826	HIST2H3D///HIST2H3A///HIST2H3C
0.097273	2.66E-05	2.850318	PSMA1
0.089394	2.75E-05	2.821273	MPHOSPH10
0.122424	2.81E-05	2.801231	FH
-0.11939	2.82E-05	2.79495	FPGT-TNNI3K///FPGT
0.090303	2.86E-05	2.782886	ZNF791
0.125455	2.93E-05	2.761976	RCN2
-0.09485	3.09E-05	2.710475	VWA8
0.133333	3.13E-05	2.699345	PMPCB
0.097273	3.21E-05	2.673924	SCCPDH
0.110303	3.26E-05	2.660084	PDHB
0.094242	3.31E-05	2.643956	PIGL
-0.09242	3.37E-05	2.628339	HSPA1B///HSPA1A
-0.09242	3.37E-05	2.628339	HSPA1B///HSPA1A
-0.11091	3.44E-05	2.609202	TIGD5
-0.10273	3.46E-05	2.60337	TRIB1
-0.10182	3.47E-05	2.600152	ZDHHC20
0.091212	3.48E-05	2.598737	LCMT1
0.124848	3.59E-05	2.567162	AGPHD1
0.113636	3.65E-05	2.553621	FKBP3
0.100909	3.65E-05	2.553519	C7orf31
-0.12758	3.74E-05	2.52903	WDR74///RNU2-2///RNU2-1
-0.15545	3.78E-05	2.519773	SNORD50A
-0.09939	3.80E-05	2.513373	FAM203A
-0.15636	3.85E-05	2.501001	HIST2H3D///HIST2H3A///HIST2H3C
0.09	3.93E-05	2.481757	SLC30A7
0.105455	3.98E-05	2.469672	RSL24D1
-0.09848	4.04E-05	2.456017	S1PR4
0.138485	4.07E-05	2.449589	C10orf57
0.127879	4.08E-05	2.446352	CTSS
0.142727	4.19E-05	2.421147	NAE1
-0.09758	4.20E-05	2.418745	TRMT1
0.114242	4.28E-05	2.402632	MCCC1
0.108485	4.35E-05	2.385935	STARD3NL
-0.09364	4.38E-05	2.380609	LPCAT1
0.136364	4.42E-05	2.371052	ZNF440
0.107273	4.42E-05	2.370593	MFSD8

0.107879	4.49E-05	2.356516	C12orf29///CEP290
0.084848	4.49E-05	2.356407	COX17
-0.08606	4.53E-05	2.346819	MTHFD1L
-0.14182	4.54E-05	2.345334	SNORD30
0.09	4.61E-05	2.330393	TMEM170A
0.121515	4.69E-05	2.314077	PNN
0.111212	4.69E-05	2.313833	DHRS1
0.164545	4.77E-05	2.299575	UBLCP1
-0.14667	4.77E-05	2.297758	HIST2H3D///HIST2H3A///HIST2H3C
0.144545	4.79E-05	2.294542	KLHDC2
-0.09303	4.88E-05	2.277137	KIF11
0.087879	4.89E-05	2.27437	TMEM199
-0.09636	4.92E-05	2.268425	BOP1
0.157879	4.94E-05	2.264926	SNRPA1
0.154545	4.95E-05	2.264059	TNFSF10
0.096364	4.95E-05	2.263227	TRIP4
-0.11424	4.97E-05	2.259694	SLC25A47
-0.09273	4.97E-05	2.25944	PPP1R15B
0.123939	4.97E-05	2.259176	PDCD1LG2
0.151212	4.99E-05	2.255032	DPY19L4
-0.10182	5.00E-05	2.253312	PCDHA4
0.106364	5.06E-05	2.242038	MIR155///MIR155HG
0.107576	5.10E-05	2.23608	STRADB
0.105152	5.11E-05	2.233166	MPC2
0.09697	5.12E-05	2.232137	MEAF6
-0.08273	5.12E-05	2.230787	BRAT1
0.085152	5.19E-05	2.218627	FYTTD1
0.113333	5.20E-05	2.216308	CCDC109B///CASP6
0.112424	5.45E-05	2.172281	NSRP1
0.137576	5.57E-05	2.151486	DSCC1
-0.09485	5.60E-05	2.14607	RRP12
0.119091	5.78E-05	2.11563	FMO4
-0.21939	5.88E-05	2.100115	RNU4-1
0.092424	5.96E-05	2.087811	HTATSF1P2///NQO2
-0.08636	5.99E-05	2.082966	ARHGAP4
-0.13	6.01E-05	2.079465	SNORA23
-0.09455	6.12E-05	2.062249	QTRT1///DNM2
-0.08455	6.12E-05	2.061302	GRK6
0.088182	6.19E-05	2.0516	SLC35B4
-0.07455	6.19E-05	2.050288	ZC3HAV1
0.08303	6.23E-05	2.045272	MR1
-0.09909	6.31E-05	2.032572	C9orf163
0.290303	6.41E-05	2.018074	SNORA14A
0.11	6.41E-05	2.01767	ZNF273
-0.08485	6.47E-05	2.008752	GLUD2///GLUD1
0.090909	6.50E-05	2.004537	PSMD9
0.094242	6.69E-05	1.976994	FNTA

-0.10394	6.77E-05	1.965462	COG5
0.08303	6.81E-05	1.960292	ANAPC16
0.140303	6.89E-05	1.949662	FAM103A1
0.127273	6.96E-05	1.938717	42066
0.076061	7.00E-05	1.933743	RAB33B
-0.09485	7.07E-05	1.923813	SNORA71C
0.128182	7.26E-05	1.899776	CAMLG
0.136061	7.34E-05	1.889292	C11orf75
-0.0903	7.35E-05	1.887548	ARMC6
0.087273	7.42E-05	1.878877	DUSP11
-0.10394	7.49E-05	1.869633	TSN
0.128485	7.50E-05	1.868546	ZNF267
0.094242	7.86E-05	1.823937	COMMD8
-0.15212	7.90E-05	1.818921	HIST2H2AA4///HIST2H2AC///HIST2H2AA3
-0.15212	7.90E-05	1.818921	HIST2H2AA4///HIST2H2AC///HIST2H2AA3
0.092727	7.97E-05	1.810479	C18orf32
0.082121	8.07E-05	1.798531	DIABLO
0.142727	8.10E-05	1.794387	BRD7
0.128485	8.12E-05	1.792935	TMEM14A
0.094848	8.15E-05	1.788931	EIF3M
0.122424	8.18E-05	1.784987	SGTB
0.114545	8.22E-05	1.781051	KRR1///GLIPR1
-0.10576	8.24E-05	1.778543	PLAC8
0.112424	8.29E-05	1.772839	AP3S1
0.081515	8.33E-05	1.76832	GADD45A
0.110606	8.57E-05	1.740864	IL3RA
0.110606	8.57E-05	1.740864	IL3RA
0.113939	8.66E-05	1.73153	SERINC1
0.097273	8.74E-05	1.722188	DLD
0.118182	8.77E-05	1.719012	DEGS1
-0.14818	8.79E-05	1.717047	NF1
-0.14818	8.79E-05	1.717047	NF1
0.108485	8.94E-05	1.700234	PLRG1
0.103939	9.05E-05	1.688916	ZNF700
-0.08636	9.09E-05	1.685148	NOL6
0.079697	9.16E-05	1.677403	ABHD3
0.082727	9.18E-05	1.675806	NDUFS2
-0.07485	9.25E-05	1.667847	MKI67
-0.11515	9.26E-05	1.667522	LRIT1
0.075455	9.35E-05	1.658007	COMMD1
0.168182	9.38E-05	1.654866	SNX24
0.097879	9.39E-05	1.653991	ZDHHC13
-0.07576	9.41E-05	1.652134	AHCTF1
0.100909	9.53E-05	1.640052	CD86
-0.15212	9.65E-05	1.627449	SNORA75
0.094848	9.66E-05	1.626972	ZNF354A
-0.08242	9.66E-05	1.626502	PHF19

0.097576	9.67E-05	1.626129	GBP3///GBP1
0.123939	9.72E-05	1.621107	SDCBP
-0.08364	9.85E-05	1.608052	DDX51
-0.1603	0.0001	1.58904	SNORD6
-0.09212	0.0001	1.588985	ORAI2
0.089091	0.000102	1.577272	MRPS21
-0.0897	0.000103	1.565133	PDE7A
0.12303	0.000103	1.561306	ACTR10
0.09697	0.000105	1.549596	MAP2K6
0.087576	0.000105	1.54727	CRLS1
0.109394	0.000105	1.545806	ZNF766
0.093939	0.000105	1.542986	NUPL2
0.145455	0.000106	1.542316	KRCC1
-0.08879	0.000106	1.538337	FLNA
0.09	0.000106	1.537553	PLA1A
0.082727	0.000106	1.536787	CHP1
0.100909	0.000108	1.519663	ZNF326
-0.08061	0.00011	1.503271	SUFU
-0.10273	0.00011	1.499182	TELO2
-0.12545	0.000111	1.495624	ZBTB41
0.079394	0.000111	1.492178	ACP1
-0.0897	0.000111	1.491261	TACC3
0.102424	0.000112	1.486896	TRAPPC6B
0.134242	0.000113	1.476528	DNAJC24
0.081515	0.000113	1.474714	CCT2
0.129697	0.000113	1.473453	GSKIP
0.10697	0.000115	1.463207	CD84
0.09697	0.000118	1.436927	APOO
-0.08727	0.000118	1.434564	TLN1
-0.12364	0.000119	1.430189	ANKRD33B
-0.08576	0.000119	1.428186	UBE3B
0.11303	0.000119	1.42696	PRCP
0.07303	0.00012	1.419208	COPS3
0.107879	0.000121	1.414157	DECR1
-0.09212	0.000121	1.412922	FAM203A
-0.1203	0.000121	1.411753	FAM207A
-0.09758	0.000122	1.406337	LLGL1
0.113636	0.000122	1.403326	BCAS2
0.181818	0.000123	1.398872	SNORA40///TAF1D
0.108788	0.000124	1.390173	CRYZL1
-0.09697	0.000125	1.383921	LOC441179///C6orf123
-0.09697	0.000125	1.383921	LOC441179///C6orf123
-0.08364	0.000126	1.375403	MRM1
0.114242	0.000126	1.373848	AUNIP
-0.09909	0.000126	1.369385	CADM3
0.124545	0.000127	1.365983	HMGN5///SH3BGRL
-0.07121	0.000127	1.362036	TFRC

0.107576	0.000128	1.361291	MELK
0.167273	0.000128	1.355807	ZNF730
-0.10788	0.00013	1.342334	SHISA9
0.111212	0.00013	1.342189	XRCC2
0.072727	0.00013	1.341218	PMS2P6///LOC441259///PMS2P3///PMS2P5
0.132121	0.00013	1.340683	FAM103A1
0.102121	0.000131	1.337077	C14orf105
-0.09909	0.000132	1.327179	TMEM43
0.076364	0.000133	1.323519	TRIM68
0.093636	0.000133	1.319625	TIMM17A
0.245758	0.000133	1.317826	CTH
0.111818	0.000135	1.306423	LSM1
0.106667	0.000137	1.290401	NOXRED1
0.07303	0.000139	1.281398	PMS2P6///LOC441259///PMS2P5///PMS2L2
-0.10424	0.00014	1.274635	MIR200B
0.073333	0.000141	1.268229	RBX1
0.116364	0.000141	1.265262	C9orf64
-0.11848	0.000142	1.255815	WDR74///RNU2-2///RNU2-1
0.105758	0.000144	1.248221	RIT1
0.080606	0.000146	1.232247	ASAH1
0.12	0.000147	1.225484	PIGB///CCPG1
0.101212	0.000147	1.225106	ZNF280B
0.10303	0.000147	1.222709	COCH
-0.15333	0.000148	1.217114	SNORD50B
0.165455	0.000149	1.213184	ALOX5AP
0.11	0.00015	1.207157	GTF2B
-0.09424	0.00015	1.207042	CPSF1
0.100606	0.00015	1.204598	SERF1B///SERF1A
0.100606	0.00015	1.204598	SERF1B///SERF1A
0.100606	0.00015	1.204598	SERF1B///SERF1A
0.091212	0.000152	1.193743	EIF2S1
-0.13394	0.000152	1.192647	HIST2H2AB
0.102121	0.000152	1.192455	TNFRSF17
-0.11152	0.000152	1.191216	LARGE
0.098182	0.000154	1.182347	RAB11A
-0.11515	0.000154	1.180424	FKTN
0.087576	0.000157	1.161274	L2HGDH
0.091818	0.000158	1.155936	ZNF642
0.097879	0.000158	1.155126	RNASET2
0.152121	0.000159	1.14729	FAM206A///IKBKAP
0.10697	0.00016	1.141624	MRPL33
0.102727	0.000161	1.139977	C10orf25///ZNF22
-0.11091	0.000162	1.134672	TMPRSS9///TIMM13
0.104242	0.000162	1.133247	PIGX
0.085152	0.000163	1.126671	ENOPH1
-0.11576	0.000163	1.124377	HIST1H3D///HIST1H2AD
0.116061	0.000164	1.122856	PPAP2A

-0.11	0.000164	1.120036	HMHA1
0.10303	0.000165	1.114828	RFC1///RFC1
0.13	0.000165	1.114394	ZNF136
0.095152	0.000166	1.11046	DNAJC21
0.087273	0.000166	1.108075	TMEM14C
-0.11273	0.000167	1.10375	ARVCF
0.099394	0.000167	1.102639	GBAS
0.079091	0.000168	1.099828	CLECL1
-0.08667	0.000168	1.099559	TUBGCP3
-0.0903	0.000168	1.096096	VPS51
-0.09636	0.00017	1.088075	GABPB1
-0.09061	0.00017	1.086366	WDR81
-0.11212	0.000171	1.077736	WDR4
0.116667	0.000172	1.074041	PSMC6
0.070606	0.000173	1.071284	POLR2H
-0.10364	0.000173	1.068221	SACM1L
0.070606	0.000174	1.064313	KLHDC3
-0.07121	0.000174	1.062038	PRKCB
0.112727	0.000177	1.044837	ZNF175
0.099697	0.000181	1.028432	PGRMC2
0.096364	0.000181	1.028359	KYNU
-0.08152	0.000182	1.02061	KIAA1432
0.082727	0.000183	1.014405	TMEM18
-0.07364	0.000184	1.010867	USP22
-0.10394	0.000184	1.008529	BCAN
0.09303	0.000184	1.007854	ATG14
0.102424	0.000184	1.007629	GINM1
0.114545	0.000185	1.003996	CEP19
0.144545	0.000185	1.003795	CENPQ
0.101515	0.000186	1.001199	ZNF850
-0.12758	0.000186	1.000917	WDR74///RNU2-2///RNU2-1
-0.12758	0.000186	1.000917	WDR74///RNU2-2///RNU2-1
-0.12758	0.000186	1.000917	WDR74///RNU2-2///RNU2-1
-0.12758	0.000186	1.000917	WDR74///RNU2-2///RNU2-1
-0.12758	0.000186	1.000917	WDR74///RNU2-2///RNU2-1
-0.12758	0.000186	1.000917	WDR74///RNU2-2///RNU2-1
-0.12758	0.000186	1.000917	WDR74///RNU2-2///RNU2-1
-0.12758	0.000186	1.000917	WDR74///RNU2-2///RNU2-1
-0.12758	0.000186	1.000917	WDR74///RNU2-2///RNU2-1
-0.06545	0.000188	0.991558	NCAPD2
0.089394	0.000188	0.991418	SEC11A
0.079394	0.000188	0.991236	ZNF85
-0.09	0.000188	0.991088	PLA2G2E
0.109697	0.000191	0.972081	DHRS7
-0.10091	0.000192	0.969086	MBD3
-0.09333	0.000192	0.968793	SBF1
-0.07788	0.000194	0.960894	ABCC1

-0.09333	0.000194	0.957187	MDS2
0.088485	0.000195	0.956382	ATP1B1
-0.09545	0.000196	0.951211	DHX37
-0.08455	0.000196	0.949284	LIMS3L///LIMS3-LOC440895///LIMS3///LIMS1
-0.08455	0.000196	0.949284	LIMS3L///LIMS3-LOC440895///LIMS3///LIMS1
0.088485	0.000197	0.943518	MITD1///MITD1
-0.09606	0.000198	0.939673	ARHGAP27
0.099394	0.000199	0.932792	LRRC49
0.112121	0.0002	0.932535	SGCB
0.121212	0.000201	0.925924	SRD5A3
0.109394	0.000202	0.922255	MB21D1
0.080909	0.000203	0.915102	DHPS
-0.12	0.000206	0.900295	LOC100128501
0.140303	0.000206	0.899889	SPC25
0.108182	0.000207	0.897299	TWSG1
-0.10455	0.000207	0.896324	BLOC1S4
0.139394	0.000208	0.893688	FAM49B
-0.07364	0.000208	0.893168	WNK1
0.085455	0.000208	0.892814	YWHAH
-0.09212	0.00021	0.885271	FAM120A
0.110303	0.00021	0.883706	TDP2
0.106364	0.000211	0.880064	ANXA4
0.072424	0.000212	0.876503	LOC100132832///DTX2P1-UPK3BP1-PMS2P11
0.136061	0.000213	0.871444	LEO1
0.075152	0.000213	0.871386	FAR1
0.073636	0.000213	0.868905	MRPL18
0.130606	0.000214	0.865319	SRR///TSR1
0.100303	0.000215	0.861819	EID1
0.114242	0.000215	0.859917	ZNF738
0.204545	0.000215	0.859626	IFT74
-0.08182	0.000216	0.857358	VARS
-0.08182	0.000216	0.857358	VARS
-0.08636	0.000216	0.855409	ANAPC2
0.107879	0.000216	0.854754	GALM///GALM
0.078485	0.000216	0.854622	MDH1
0.111212	0.000217	0.852429	GGNBP2
0.071515	0.000222	0.832158	PMS2P6///LOC441259///PMS2P5///PMS2P1
-0.12061	0.000224	0.823365	GLTP
0.093333	0.000224	0.820949	SLC25A40
-0.0903	0.000229	0.799985	CTF1
0.083636	0.000229	0.799207	NDUFC2
-0.20909	0.000229	0.79877	LOC100133299
0.158182	0.000231	0.79051	CCDC53
0.078788	0.000232	0.78895	LOC653653///AP1S2///AP1S2
0.098485	0.000232	0.788652	ZNF833P
0.115758	0.000234	0.777498	LIPH
0.072424	0.000235	0.774747	NDUFAB1

0.108182	0.000235	0.773936	PTGR1
-0.08909	0.000236	0.771382	GPCPD1
0.071515	0.000236	0.769379	CRCP
0.109697	0.000237	0.766488	FAM96A
0.095152	0.000238	0.763464	TFB1M///TIAM2
-0.15939	0.000239	0.760071	HIST1H2AI
-0.11061	0.000239	0.758705	GFOD1
0.096364	0.000241	0.753168	SCP2
0.068485	0.000242	0.748817	LETMD1
-0.09636	0.000242	0.748059	SLC35E2B///SLC35E2
-0.12091	0.000244	0.740741	HS3ST3A1
-0.11121	0.000244	0.739669	PNMT
-0.09727	0.000246	0.732441	CTU1
0.126667	0.000246	0.731266	IFNGR1
0.127879	0.000246	0.730466	C17orf75
-0.08606	0.000249	0.718866	HIST2H2BC///HIST2H2BE
-0.10364	0.00025	0.715164	XAGE3
0.094242	0.000251	0.71292	PSMA6
-0.08758	0.000251	0.710578	SNORA71A
0.072727	0.000252	0.709711	PNISR
0.124545	0.000252	0.70793	SNORD4B
-0.0703	0.000252	0.706525	SCAMP1
0.080606	0.000253	0.705596	TADA2A
-0.15576	0.000258	0.685861	PTPLAD1
0.128788	0.000259	0.683306	RPL22L1
-0.0797	0.000262	0.669552	CAD
0.099394	0.000263	0.668035	CSTF2
0.18303	0.000265	0.661058	SLC9B1
0.08	0.000265	0.659661	DCUN1D3///LYRM1
-0.06545	0.000266	0.655941	MLL5
0.127273	0.000268	0.650081	SNX2
-0.1003	0.000268	0.647843	PCDHA7
0.119091	0.00027	0.643097	MAB21L3
0.07	0.00027	0.642679	PEX19
0.083939	0.00027	0.642034	MRPL9
0.131818	0.000271	0.640316	FAM210A
-0.08697	0.000272	0.634122	HIST1H2BA
-0.08515	0.000273	0.633031	FDXR
0.155455	0.000276	0.622789	SC5DL
0.097273	0.000276	0.619725	N4BP2L1
0.09303	0.000277	0.618994	PDCD10
0.128485	0.000277	0.618574	FBXO16///ZNF395
-0.07333	0.000277	0.618546	FOCAD
0.13	0.000278	0.615344	EXOSC8
0.096364	0.000279	0.611418	SRP54
0.103636	0.00028	0.607712	UBA3
0.098485	0.00028	0.607383	C4orf29

0.070909	0.000282	0.599449	C15orf40
0.099697	0.000285	0.590042	HAUS1
-0.08364	0.000286	0.58604	NAA25
0.104242	0.000287	0.582532	C4orf52
0.099091	0.000289	0.578236	GK5
0.079697	0.000291	0.569107	RBMXL1///CCBL2
0.106667	0.000292	0.568004	TIMM8A
-0.08273	0.000292	0.565928	LOC727799///HEATR2
0.105758	0.000297	0.550674	GCLC
0.072727	0.000299	0.542802	ERV3-1
0.098182	0.000302	0.534742	MCTS1
0.104848	0.000302	0.534572	RRP36
0.086364	0.000303	0.532594	TIFA
-0.08121	0.000304	0.527386	CTDP1
0.082727	0.000305	0.524709	METTL17
0.105758	0.000307	0.517603	NAA20
0.118788	0.000308	0.516216	VAMP4
0.069091	0.000309	0.512569	TMEM50A
-0.11303	0.000312	0.50204	EFR3B
-0.0697	0.000313	0.500089	TBCK
0.071515	0.000314	0.498001	SLC30A6
0.068485	0.000315	0.492915	PRPF3
0.177576	0.000315	0.492846	ZNF90
-0.0797	0.000316	0.492321	TUBGCP6
0.117879	0.000318	0.483638	LTV1
0.077879	0.000319	0.480914	SLC25A17
0.127576	0.00032	0.478751	OPN3///KMO
-0.07212	0.000322	0.473497	PI4KA
-0.10333	0.000322	0.472768	CDK5R1
0.066061	0.000323	0.470067	PCMTD2
0.107576	0.000324	0.46788	MRPL42
0.086061	0.000325	0.464743	HSD17B7P2///HSD17B7
-0.07394	0.000326	0.462006	NCLN
-0.07636	0.000326	0.460991	USP25
0.11	0.000328	0.4567	ZNF350
0.06697	0.000328	0.455721	MPV17///MPV17
0.108182	0.000328	0.454036	HAUS2
0.115758	0.00033	0.449517	CXorf21
0.078788	0.00033	0.449043	PSMA2
0.102727	0.000333	0.439746	MPP1
0.113636	0.000335	0.434329	GLMN
0.097273	0.000336	0.43107	CEP135
0.098485	0.000338	0.425375	MT1F
0.126364	0.000339	0.424802	ERP27
0.105152	0.000343	0.412775	TEFM
0.110909	0.000349	0.395689	DEM1
-0.12727	0.000352	0.388343	ACR

-0.17606	0.000353	0.383632	HIST1H3F
0.13697	0.000355	0.37975	LARP7///C4orf21///LARP7
-0.13758	0.000355	0.37888	FAM43A
0.069697	0.000355	0.378867	UBXN4
0.097273	0.000356	0.37703	MRPL47
0.097576	0.00036	0.36565	C8orf40
0.134545	0.000361	0.363827	IL2RA
0.085758	0.000362	0.359337	PCMT1
0.113333	0.000363	0.358512	PBK
0.088788	0.000364	0.355492	CHAF1B
0.116364	0.000365	0.352862	GGH
0.150606	0.000365	0.352204	NDUFB6
0.149091	0.000367	0.348333	CDK1
0.113636	0.000367	0.347794	MOSPD2
0.113636	0.000369	0.342397	MED10///DNAL4
-0.06061	0.000369	0.342231	PRKDC
0.133333	0.000371	0.337411	ACER3
0.108788	0.000371	0.337337	CRIPT
0.094545	0.000371	0.336204	BRD7
0.072727	0.000372	0.333829	MTFR1
0.094545	0.000374	0.32999	LRRC42
0.117576	0.000374	0.329907	NT5C3
-0.08515	0.000374	0.328171	ZNF710
-0.07939	0.000375	0.326846	LCORL///NCAPG
0.089697	0.000376	0.324936	TCTN1
-0.06212	0.000377	0.321818	KDM5C
-0.09848	0.000378	0.318119	ZRANB1
-0.08879	0.000384	0.302712	GUCY1A2
0.064242	0.000385	0.301706	DTX2P1-UPK3BP1-PMS2P11///LOC441259
0.133333	0.000385	0.300088	CFLAR
0.080909	0.000386	0.299139	ARFGAP3
-0.08303	0.000387	0.297218	SAMD4B
-0.07455	0.000387	0.296633	TCOF1
-0.08364	0.000389	0.290609	AP1B1
0.093636	0.000392	0.284982	ZNF260
0.179697	0.000392	0.284524	C8orf37
0.100303	0.000392	0.283905	ZC2HC1A
0.092121	0.000392	0.282705	IDI1
0.11697	0.000395	0.275497	BIN2
-0.0697	0.000396	0.273334	TSC2
0.080606	0.000397	0.272693	TMEM59///TMEM59
-0.06909	0.000398	0.269658	TRPC4AP
0.118788	0.000398	0.268588	PPIL4
-0.0597	0.0004	0.263339	PLCG2
0.128788	0.000403	0.257869	AS3MT
0.101212	0.000405	0.251923	RAD54B
-0.07879	0.000407	0.246795	DPH2

0.097273	0.000412	0.23589	PGM3
-0.0797	0.000415	0.229913	SCAP
-0.08545	0.000415	0.228489	CHST2
0.091818	0.000419	0.220045	MLLT11
-0.09818	0.00042	0.217979	INTS1
0.070606	0.000421	0.215224	ZC3H15
0.089091	0.000423	0.211377	APH1B
0.086061	0.000424	0.207712	RPP30
-0.07424	0.000425	0.207065	CYFIP2
0.075758	0.000425	0.20574	CFLAR
0.082121	0.000426	0.20428	STRAP
0.095455	0.000429	0.197295	CCDC28A
-0.09788	0.00043	0.195333	UCN3
0.16	0.00043	0.194697	FGL2
0.064545	0.000431	0.193522	HVCN1
-0.09818	0.000432	0.190339	CCDC48
-0.06212	0.000433	0.188464	ERBB2IP
0.122424	0.000434	0.186188	FBXO4
0.091818	0.000438	0.177454	KLHL2
0.072121	0.000446	0.160268	RDH11
-0.07939	0.000446	0.159913	ST14
0.105152	0.000449	0.153321	DIRC2
0.130909	0.000452	0.146632	SERPINB10
-0.0903	0.000453	0.144882	SVIP
0.096364	0.000453	0.144761	ARV1
0.115152	0.000453	0.144102	MIRLET7G
0.063636	0.000454	0.143406	C18orf8///NPC1
0.105152	0.000454	0.142673	IQCG
0.132121	0.000454	0.1422	ZNF726
0.109697	0.000455	0.139755	LAMTOR5
-0.05818	0.000456	0.139452	DOCK8
-0.08424	0.000458	0.134885	PDE6G
-0.06727	0.000459	0.133182	CNDP2
0.064848	0.000462	0.12515	LAP3
0.067879	0.000463	0.122994	GOLGA5
-0.07667	0.000466	0.11865	RRP9
-0.07152	0.000466	0.118505	TICRR
-0.06697	0.000466	0.117344	DDI2///RSC1A1
-0.07152	0.000468	0.113443	SMTN
-0.08485	0.000468	0.113334	NAT8L
0.078788	0.000469	0.11059	TMEM57
-0.06273	0.00047	0.108695	TNPO3
0.077879	0.000471	0.108071	GRPEL2
-0.09485	0.000473	0.102846	LOC100129110///MORN1
-0.07455	0.000474	0.100453	TAF2
0.103939	0.000476	0.098001	KIAA0101
0.104242	0.000482	0.08595	SLC7A7

0.059697	0.000484	0.082064	PSMC1
-0.06909	0.000485	0.078992	RASAL3
-0.13697	0.000486	0.077846	HIST1H1B
0.078485	0.000487	0.074702	SBDS
-0.11636	0.000487	0.074672	HAUS6
0.072121	0.000489	0.0707	C12orf23
-0.10364	0.00049	0.068609	PLXNB2
0.102121	0.000491	0.067706	C6orf201///ECI2
0.083333	0.000492	0.066356	SAR1A
-0.15091	0.000494	0.061964	CHST15
0.096667	0.000495	0.06044	LOC96610///BMS1
-0.07152	0.000496	0.058313	ANKRD13B
0.093636	0.000496	0.05804	ERGIC2
0.099394	0.000497	0.056464	VPS29
0.067879	0.000498	0.053402	CCDC77
0.083939	0.000498	0.053177	REEP5
-0.06727	0.0005	0.050891	CKAP5
-0.06636	0.000501	0.048751	AHR
0.072727	0.000501	0.048066	POP4
0.097879	0.000502	0.04649	HTATIP2
0.072424	0.000503	0.044589	HEATR1///LGALS8
0.143636	0.000503	0.043489	PTPN12
0.141515	0.000505	0.040352	CRYM
0.093636	0.000509	0.032081	GTF3C6
0.061212	0.00051	0.030261	EZH2
0.059697	0.000511	0.028662	H3F3AP4///H3F3A
0.115455	0.000513	0.025959	NUCB2
0.09697	0.000514	0.023352	RPL39L
0.058182	0.000515	0.022287	PSMC1
0.147879	0.000518	0.016459	COX11
0.073939	0.000518	0.01644	EMC4
0.1	0.00052	0.011947	TTC8
-0.08152	0.000522	0.008498	MGAT1
0.115758	0.000524	0.005284	UPRT
0.073333	0.000525	0.002132	TADA1
0.064848	0.000528	-0.00182	HDDC2
-0.06576	0.000528	-0.00184	WWP2
0.060303	0.000529	-0.00388	JAGN1
0.10697	0.000529	-0.00401	PGRMC1
0.08	0.000531	-0.00754	KTN1
0.07697	0.000532	-0.00919	LANCL1
0.092727	0.000534	-0.01319	ZNF438
0.069394	0.000534	-0.01335	C1QTNF3-AMACR///AMACR
0.072121	0.000534	-0.01391	DYNLL2
-0.10879	0.000535	-0.01536	FER1L4
0.071818	0.000536	-0.01717	YARS2
0.077273	0.000536	-0.0177	ADK

0.079091	0.000539	-0.02205	GINS4
0.084848	0.00054	-0.02364	ELP4
-0.0797	0.00054	-0.02365	OGFR
0.078485	0.000544	-0.03121	PSMD10
-0.11909	0.000545	-0.03245	SNORA65
0.089091	0.000549	-0.04039	LUC7L3
0.109394	0.00055	-0.04245	EFCAB7
0.116667	0.000554	-0.04934	SGCE
0.106667	0.000555	-0.0503	COPS4
-0.06606	0.000556	-0.05191	ZBTB38
-0.11364	0.000556	-0.05287	BMPR1A
-0.09273	0.000557	-0.05473	MOCS3///MOCS3
0.072727	0.000562	-0.06235	SLC50A1
0.092121	0.000563	-0.06404	FUCA1
-0.12212	0.000564	-0.06614	HIST3H2BB
0.08303	0.000567	-0.07018	SUCLA2
0.089091	0.000567	-0.07119	PXMP2
0.129394	0.000567	-0.07158	CDK7
0.129394	0.000567	-0.07158	CDK7
0.085758	0.00057	-0.07673	CDC5L
0.09303	0.000572	-0.08011	DPM1
-0.08879	0.000574	-0.08361	PODNL1
-0.08545	0.000578	-0.08903	ADAM23
-0.07818	0.00058	-0.09228	CSNK2A1P///CSNK2A1
-0.07758	0.000581	-0.0947	SLC39A8
-0.09364	0.000583	-0.09719	HSF2BP
0.126061	0.000585	-0.10091	C12orf5
-0.06636	0.000586	-0.10259	HUWE1
-0.08152	0.000591	-0.11126	NUP210
-0.0803	0.000592	-0.11277	EHD1
0.07	0.000593	-0.11425	SSFA2///SSFA2
0.118182	0.000594	-0.11523	NDUFS4
-0.06939	0.000595	-0.1171	PFKL
-0.0997	0.000596	-0.11867	CHORDC1
0.070303	0.000596	-0.11958	PCGF1
0.069394	0.000596	-0.11958	ARHGEF9
-0.05515	0.000597	-0.12004	PABPC4
0.089394	0.000599	-0.12366	SYNJ2BP
0.112727	0.000602	-0.12923	ZNF568
-0.14121	0.000608	-0.13757	FSCN2
0.070606	0.000608	-0.13878	RFC5
0.121818	0.000608	-0.13879	ZNF224
0.071212	0.000609	-0.13985	MRPL50
0.071212	0.000611	-0.14219	MFSD5
-0.07727	0.000611	-0.14357	WDR36
-0.06879	0.000611	-0.14369	NLE1
-0.08121	0.000613	-0.1464	YWHAG

0.11303	0.000614	-0.14748	LOC400590
-0.06909	0.000614	-0.14793	HTT
0.080606	0.000616	-0.15131	NFYB
-0.06364	0.000618	-0.15425	CLASP1
0.09303	0.000619	-0.15482	LAPTM4A
0.071515	0.000619	-0.15491	MRPL44
0.072727	0.00062	-0.15766	PCNA-AS1///PCNA
0.061818	0.000621	-0.15783	RTF1
0.109697	0.000621	-0.1583	C11orf54
-0.06455	0.000623	-0.16092	JAK1
-0.06697	0.000623	-0.16099	ZC3HAV1L
0.086364	0.000624	-0.16287	MT1L
0.06697	0.000635	-0.17934	LUZP6///MTPN
0.118182	0.000636	-0.1814	C6orf211
0.109091	0.00064	-0.18751	CCDC113
0.081515	0.00064	-0.1879	RAB2B
-0.06545	0.00064	-0.18808	BCKDHB
0.108182	0.000644	-0.19323	NDUFAF6
-0.10303	0.000645	-0.19454	ABCD3
-0.10576	0.000648	-0.19958	BAK1
-0.06212	0.000649	-0.2009	REPIN1
0.103939	0.00065	-0.20208	INPP1
-0.13333	0.000655	-0.2091	LOC100128830
0.184545	0.000659	-0.21617	LY96
0.090303	0.000661	-0.21843	SLC22A25
0.103333	0.000663	-0.22188	RIOK2
0.069394	0.000663	-0.22212	COX17
-0.07	0.000667	-0.22702	CAMK1D
-0.05818	0.000669	-0.2299	DUS3L
-0.07818	0.00067	-0.23119	INPP5D
-0.12788	0.000671	-0.23276	SNORA52
0.084242	0.000673	-0.23582	SMU1
0.166667	0.000676	-0.24047	GNPDA2
0.102727	0.000678	-0.24243	LOC100134868
-0.09212	0.000679	-0.24376	LHX5
0.101818	0.00068	-0.24567	CHIC2
0.136061	0.000681	-0.24743	SNORD36B
-0.08636	0.000687	-0.2562	SYMPK
0.077273	0.00069	-0.26045	LOC100128988
-0.06121	0.000691	-0.26059	DNMBP
-0.08364	0.000691	-0.26124	WDR18
-0.06697	0.000691	-0.26181	TNPO2
0.093333	0.000691	-0.26196	TSPAN15
0.090303	0.000693	-0.26347	IFT46
-0.09727	0.000696	-0.26864	ARHGAP42
-0.09152	0.000697	-0.26917	ANKMY1
0.105152	0.000697	-0.26934	IFT52

0.100606	0.0007	-0.27325	BCL2A1
0.106061	0.0007	-0.2739	POLE2
0.124848	0.0007	-0.27426	ZNF616
0.084848	0.000702	-0.27681	GDPD3
-0.10545	0.000703	-0.27716	ZIC2
0.09303	0.000704	-0.27916	SPTLC1
0.086061	0.000705	-0.28003	COX16
0.1	0.000708	-0.28413	DCUN1D5
0.129394	0.000713	-0.29189	PEX3
-0.07303	0.000714	-0.29322	FCGR3B///FCGR3A
0.080909	0.000715	-0.2937	PHAX
-0.07394	0.000715	-0.29427	PPP2R5D
-0.05848	0.000716	-0.29583	POLR2A
0.121515	0.000719	-0.29998	EFCAB2
0.087879	0.00072	-0.30035	FUNDC1
0.079394	0.000724	-0.30605	PDK3
0.073939	0.000724	-0.3066	GCSAM
0.108788	0.000726	-0.30818	NEK3
-0.06515	0.000726	-0.30838	CERKL///ITGA4
0.111212	0.000729	-0.31293	RWDD2A
0.079394	0.000729	-0.31316	C20orf72///C20orf72
0.072121	0.000731	-0.31502	RBM18
0.078788	0.000731	-0.3154	UBXN1
0.091212	0.000731	-0.31553	YIPF1
-0.06273	0.000738	-0.32428	YWHAB
0.078485	0.000738	-0.32429	PRPF38B
-0.07636	0.000738	-0.32466	LRRC24///C8orf82
-0.12061	0.000738	-0.32521	SNORA7B
0.070909	0.000744	-0.33266	DRAM2
0.081212	0.000748	-0.33771	ACOT13
0.065758	0.000749	-0.33881	MLH1
-0.07121	0.000749	-0.33907	TTLL12
0.099394	0.000752	-0.34315	ZNF284
0.082727	0.000754	-0.3449	NHEJ1
-0.08515	0.000755	-0.34633	SCFD2
0.089697	0.000757	-0.3485	ZNF442
-0.09121	0.000763	-0.3569	PCDHA8
-0.07121	0.000766	-0.36086	CCNI
-0.05848	0.000769	-0.36415	GATAD2A
0.120606	0.000771	-0.3663	RBM48
-0.07758	0.000772	-0.36818	SERAC1
-0.10333	0.000774	-0.37054	GAS1
0.105455	0.000778	-0.37535	LOC100506127
-0.07333	0.000778	-0.37584	DIS3L2
0.073636	0.000779	-0.37685	UBXN7
0.091818	0.000779	-0.37717	HSPB11
0.069697	0.000782	-0.37973	ABHD10

0.080606	0.000782	-0.38065	CXorf26
-0.07515	0.000784	-0.38295	SNORD55
0.094848	0.000785	-0.38366	ZRSR2
0.087576	0.000788	-0.38769	ACAD8
0.101515	0.000794	-0.39447	RAP1B
-0.08788	0.000794	-0.39486	HCFC1
-0.13424	0.000797	-0.39823	HIST1H4J///HIST1H4K
-0.07515	0.0008	-0.40223	TCF3
0.083333	0.0008	-0.40238	HNRNPA3
-0.07394	0.000801	-0.40345	CABIN1
0.081212	0.000803	-0.40587	EFCAB11
0.112727	0.000811	-0.41499	MTHFD2
-0.09242	0.000817	-0.42257	INSM2
0.067273	0.000818	-0.42385	C20orf24
0.096061	0.000818	-0.4241	TSFM
0.11303	0.00082	-0.42587	TXNDC9
0.075152	0.000821	-0.42709	LCTL///ZWILCH
-0.05758	0.000822	-0.42778	PKN2
0.070909	0.000824	-0.43046	ARNTL2
-0.16152	0.000824	-0.43048	LOC440944
-0.07545	0.000825	-0.43152	MAN2A1///MAN2A1
0.122727	0.000832	-0.43945	ASNS
0.07697	0.000833	-0.44108	ARHGDIB
0.069091	0.000834	-0.44269	DEDD
-0.07879	0.000839	-0.44743	RAPGEF2
0.102121	0.00084	-0.44871	RPP40
-0.07879	0.000842	-0.45087	APBA3
0.069091	0.000842	-0.45163	PDE6D
0.103333	0.000843	-0.45256	UBD
-0.06485	0.000844	-0.45357	GCN1L1
0.096667	0.000845	-0.45484	IER3IP1
0.088485	0.00085	-0.46026	OSTF1
0.105455	0.000853	-0.46357	DNAAF2
-0.07091	0.000853	-0.46443	SLC7A9
0.060606	0.000854	-0.46486	DNAJC25-GNG10///GNG10
0.077273	0.000854	-0.46489	MIS12
0.075152	0.000856	-0.46679	CNTD1///BECN1
-0.14727	0.000859	-0.47065	HMX3
-0.07909	0.000863	-0.47522	DPPA5
0.092121	0.000869	-0.48216	MTIF2
0.110606	0.000877	-0.49022	DLEU1
0.084848	0.000878	-0.49206	RPA2
-0.11939	0.000881	-0.49506	MAP3K2
-0.07667	0.000884	-0.49784	PFAS
-0.09061	0.000884	-0.49809	COL6A1
0.080606	0.000886	-0.50087	ZNF391
0.077576	0.000887	-0.50181	ZNF525///ZNF813///ZNF765///ZNF701///ZNF28

0.077879	0.000888	-0.50311	APPL1
0.078788	0.000889	-0.50361	RDX
0.097273	0.000891	-0.50598	GSTM4///GSTM2
0.102727	0.000894	-0.50898	TATDN1
0.074848	0.000896	-0.51122	CTNNAL1
0.113636	0.000899	-0.51456	ASCC1
-0.07697	0.0009	-0.51593	MORC2
-0.08182	0.000901	-0.51606	EBF3
-0.09091	0.000901	-0.51699	CASD1
0.074242	0.000902	-0.51733	ZNF57
0.087273	0.000906	-0.52156	HNRNPA3///HNRNPA3P1
0.061515	0.000907	-0.52276	MYO5A
-0.11424	0.000908	-0.52364	HIST1H2AE
0.071515	0.000909	-0.52528	XPNPEP1///XPNPEP1
-0.06727	0.00091	-0.52661	C15orf39
0.070303	0.000919	-0.5359	TSPYL2
0.06697	0.000921	-0.53765	DPY30///MEMO1
0.103636	0.000925	-0.54195	ACAT1
-0.07758	0.000926	-0.54325	GALR2
-0.07455	0.000927	-0.54425	TRIM65
0.071212	0.000933	-0.54975	H3F3AP4///H3F3A
-0.07394	0.000933	-0.55028	ALKBH5
0.068182	0.000933	-0.5504	OIP5
0.108485	0.000935	-0.55201	SERPINA1
-0.09273	0.000936	-0.55277	DAO
0.083939	0.000937	-0.55411	MINA
-0.05636	0.000938	-0.55509	XPR1
0.085455	0.000939	-0.55582	GPR89C///GPR89A///GPR89B
-0.07091	0.00094	-0.55782	NDUFB7
-0.07818	0.000941	-0.55836	PPP6R1
0.109091	0.000942	-0.55919	ZNF542
0.065758	0.000943	-0.56074	LYPLA1
-0.11909	0.000943	-0.56085	PCDHA10
0.082424	0.000944	-0.56133	PARP16
0.080606	0.000947	-0.56461	RALA
0.109697	0.000952	-0.56904	PRTFDC1///PRTFDC1
0.090606	0.000955	-0.57244	RAN
0.080303	0.000955	-0.57248	GEMIN6
-0.07182	0.000955	-0.57285	GGN
0.079697	0.000961	-0.57816	CMC4///MTCP1
-0.07394	0.000968	-0.58581	ITGB7
-0.07606	0.00097	-0.58706	EHMT2
-0.07606	0.00097	-0.58706	EHMT2
0.12303	0.00097	-0.58788	TCEAL8
-0.05303	0.000973	-0.5905	DNMT1
-0.06242	0.000979	-0.59598	ESYT1
-0.10939	0.00098	-0.59737	SOX7

-0.05909	0.00098	-0.59754	RNF10
-0.0603	0.000982	-0.59888	M6PR
0.068788	0.000982	-0.59956	MSRB1
0.083333	0.000983	-0.59988	BBS7
0.085455	0.000986	-0.60322	GMNN
-0.0697	0.000992	-0.60873	ULK4P2///GOLGA8T///GOLGA8I///ULK4P1
0.09303	0.000995	-0.61215	COQ10B
0.090909	0.000997	-0.61356	RMND1
0.067576	0.001002	-0.61838	C14orf166
0.134242	0.001002	-0.61841	RNPC3
-0.07545	0.001006	-0.62281	XXYLT1
0.099697	0.001007	-0.62317	MSANTD3
0.093333	0.001007	-0.62373	ACTL6A
-0.06606	0.001011	-0.6274	GMEB2
0.092121	0.001014	-0.62975	DUSP12
-0.06758	0.001015	-0.63153	ATP8A1
0.065455	0.001016	-0.63187	TRIP10
-0.07818	0.001017	-0.63287	THOP1
-0.11091	0.001018	-0.63388	TNFAIP8L3
0.074545	0.00102	-0.6355	LPCAT3
0.065152	0.001024	-0.63947	ST13P4///ST13P5///ST13
0.087879	0.001027	-0.64269	GHITM
0.073636	0.001037	-0.65159	SAMM50
0.124242	0.001037	-0.65173	ATXN3///ATXN8///ATXN3
0.076061	0.001038	-0.65294	TTC13
-0.11727	0.00104	-0.65473	CTAG2
0.088182	0.00104	-0.65497	GPR89C///GPR89A///GPR89B
0.169394	0.001042	-0.656	ZNF724P
-0.05455	0.001048	-0.66156	AFTPH
0.063939	0.001049	-0.66238	AMZ2
0.090303	0.001049	-0.66267	RARS
0.084242	0.00105	-0.66361	ZNF789
-0.1103	0.001055	-0.66787	VAC14
0.085758	0.001057	-0.66981	C2orf42
0.075455	0.001057	-0.66992	ATP6AP2
0.086061	0.001058	-0.67061	HIBADH
0.098182	0.001058	-0.67094	MAT2B
0.085758	0.001058	-0.67115	C5orf34
0.062727	0.001063	-0.67545	APMAP
-0.10545	0.001063	-0.67573	GATA4
-0.10697	0.001066	-0.67796	PCDHA12
-0.07273	0.001066	-0.67871	RNF213
-0.08788	0.001072	-0.6836	ESPL1
-0.06939	0.001077	-0.68788	WEE1
-0.15424	0.001077	-0.68802	HIST1H4B
0.099394	0.001078	-0.6887	C6orf225///TUBE1
-0.07545	0.001084	-0.69403	PRKAR1A

-0.07121	0.001086	-0.69595	TBATA
0.071212	0.001088	-0.69787	NSL1
-0.0797	0.001091	-0.70046	FLJ33534
0.078788	0.001093	-0.70195	NEIL3
0.072727	0.001093	-0.70273	MSRB2
-0.08697	0.001093	-0.70275	NOTCH1
0.066364	0.001095	-0.70421	FAM122C
0.092424	0.001099	-0.70753	RPP38
0.119394	0.001099	-0.70755	OAT
-0.07697	0.001103	-0.71105	ZNF746
0.058788	0.001107	-0.71419	LIMA1
-0.05879	0.001107	-0.71419	CRKL
-0.06697	0.001111	-0.71825	PRPF4
0.074242	0.001113	-0.7194	DNAJC1
0.107273	0.001114	-0.72048	METTL21D
-0.09	0.001126	-0.73083	FAM110B
0.065152	0.001127	-0.73174	PSENEN
0.094545	0.001129	-0.73389	ALG14
0.050606	0.001131	-0.73493	H3F3AP4///H3F3A
0.083636	0.001131	-0.7355	SIKE1
0.069394	0.001135	-0.7382	TMEM209
-0.08182	0.001137	-0.74008	SNX29
0.064545	0.001145	-0.74737	TYW3
0.111515	0.001149	-0.75065	TMEM159
0.069697	0.001151	-0.75211	PAIP2
0.07697	0.001152	-0.75329	ZNF124
0.088485	0.001154	-0.75479	C12orf45
-0.08	0.001157	-0.75721	E4F1
-0.0803	0.001159	-0.75905	DMRTA2
-0.06182	0.00116	-0.75993	AP2A2
0.103333	0.001162	-0.76102	LAMTOR3
-0.08818	0.001162	-0.76111	ZBTB34
0.094545	0.001165	-0.76385	CXorf65
0.111212	0.001167	-0.76572	HIBCH
-0.06212	0.00117	-0.76743	NCOR1
-0.10242	0.00117	-0.76749	ZFP36L2
0.076667	0.00117	-0.76777	SAMD8
0.08	0.001173	-0.77059	KIAA1704
0.074848	0.001175	-0.7722	CBR1
-0.06758	0.001183	-0.77841	TPP2
0.069091	0.001187	-0.78167	MANBAL
0.072727	0.001188	-0.78248	NSMCE2
0.097273	0.00119	-0.78418	ZNF643
0.121212	0.001191	-0.7849	PLGRKT
-0.07939	0.001192	-0.78583	TRAPPC9
0.095455	0.001198	-0.79041	TMEM242
0.143333	0.001199	-0.79101	BRMS1L

0.077273	0.001199	-0.79124	NUDT15///SUCLA2
-0.10485	0.0012	-0.79244	CACTIN-AS1///CACTIN
0.101818	0.001203	-0.79425	DSTNP2
-0.08879	0.001204	-0.79525	COL9A2
-0.09061	0.001206	-0.79668	TUBBP5
0.091818	0.00121	-0.80021	THOC7
0.071515	0.001216	-0.80483	EBNA1BP2
-0.09636	0.001219	-0.8072	NOL4
-0.06455	0.001221	-0.80868	KIFC1
0.056667	0.001223	-0.81046	EIF3F
0.097879	0.001224	-0.81098	WDR61
0.06697	0.001227	-0.8136	NUDT21
-0.09697	0.001229	-0.8152	TAF4B
0.063636	0.001229	-0.81537	POLR1D
-0.05606	0.001243	-0.82594	GTF3C1
-0.08485	0.001247	-0.82944	SOX2
0.072121	0.001248	-0.83	TMEM192
0.089394	0.001251	-0.83194	PCYOX1
-0.07758	0.001253	-0.83399	RBM14///RBM14-RBM4///RBM14
0.061818	0.001256	-0.8361	GLT8D1///GLT8D1
0.075152	0.001261	-0.84008	MRPS33
0.113939	0.001262	-0.8409	TPRKB
-0.06515	0.001265	-0.84278	AGRN
0.080606	0.001265	-0.84288	DDX28///DUS2L
0.090303	0.001268	-0.84524	TRMT10C
0.08697	0.001269	-0.84553	RSRC2
-0.07394	0.001269	-0.84602	IRX2
0.108788	0.001273	-0.84904	CHMP2B
0.123333	0.001275	-0.8505	SNORD61
-0.06939	0.001279	-0.85334	FLNB
0.063939	0.001279	-0.85352	USB1
-0.14909	0.001283	-0.85638	TAS2R31
0.112121	0.001285	-0.85824	NDUFAF5
0.072424	0.001286	-0.85843	ZFP3
0.103939	0.001292	-0.86311	METTL6
0.071515	0.001296	-0.86638	TMEM62
-0.11879	0.001301	-0.86966	SNORD59B
-0.0703	0.001302	-0.87023	PPP2R5E
-0.11485	0.001303	-0.87115	HIST1H4J///HIST1H4K
0.091515	0.001306	-0.87338	PSPH
-0.06909	0.001313	-0.87883	FAM190B
0.083636	0.001316	-0.88122	TAF1B
-0.07939	0.001321	-0.88425	FLJ39653
0.083333	0.001326	-0.88806	SEPSECS
-0.06182	0.001327	-0.88885	HSPA4
0.084242	0.001329	-0.89024	SQRDL
0.070303	0.001329	-0.89049	PMS2P6///LOC441259///PMS2P3///PMS2L2

-0.05212	0.001334	-0.89382	GLG1
-0.09727	0.001335	-0.89457	SLC5A4
0.093939	0.001336	-0.89507	UBD
0.085152	0.00134	-0.89794	NAT1
0.060606	0.001345	-0.90176	SRP9
0.089394	0.001345	-0.90177	DNAH6
0.061818	0.001347	-0.90306	ERP44
0.054848	0.001352	-0.90712	TMEM248
-0.05455	0.001355	-0.9088	USP28
0.060606	0.001359	-0.91158	CAT
-0.06061	0.001359	-0.91158	RNF19A
0.084242	0.001365	-0.91567	CTBS
0.091818	0.001372	-0.92094	ALG5
0.081515	0.001374	-0.92214	CDKL3
0.068788	0.001375	-0.9232	ZNF770
-0.07606	0.001375	-0.92322	KDM4B
-0.05636	0.001375	-0.92331	NONO
0.092727	0.001378	-0.92525	SNRNP25///POLR3K
-0.07152	0.00138	-0.92651	SHANK1
-0.05879	0.001381	-0.92694	HIPK1
0.072727	0.001383	-0.92838	MARVELD3///PHLPP2
0.119091	0.001383	-0.92856	USP8
0.109394	0.001386	-0.93084	ANKRA2
0.076364	0.001392	-0.93478	ANKRD36B
-0.08788	0.001393	-0.93529	CACHD1
0.089394	0.001393	-0.93544	PFN2
0.105455	0.001393	-0.93574	DNAJC15
-0.08727	0.001395	-0.93714	C5orf22
-0.07303	0.001398	-0.939	SNX19
-0.06879	0.001403	-0.94206	PPP1R16B
0.097879	0.001406	-0.94411	CNEP1R1
-0.06576	0.001406	-0.94461	MCM2
0.099394	0.001408	-0.94615	ZNF506
0.076364	0.00141	-0.94698	ZNF37BP
0.068182	0.00141	-0.947	USP27X
0.067879	0.00141	-0.94712	HADHB
0.103333	0.001412	-0.9486	GLRX
0.121515	0.001413	-0.94891	MND1
-0.09758	0.001414	-0.95012	RPS15
-0.07394	0.001414	-0.95019	CYS1
0.085758	0.00142	-0.95412	ZNF383
0.096061	0.001421	-0.95483	CCL28
-0.05182	0.001425	-0.95715	PTBP1
-0.08364	0.001426	-0.95812	GDF6
0.067576	0.001427	-0.95856	ASH2L
0.064242	0.001428	-0.95943	CREBL2
0.093333	0.001428	-0.95946	MTA3

-0.05788	0.001431	-0.96113	CCDC134
-0.07333	0.001432	-0.96228	PFN3
0.062424	0.001439	-0.96657	UPF3A
0.08	0.00144	-0.96762	PARP2
0.068485	0.001443	-0.96916	ZNF394
0.083333	0.001444	-0.97039	NRBF2
0.087576	0.001445	-0.97092	ZNF330
-0.05697	0.001446	-0.97142	EARS2///UBFD1
0.077576	0.001447	-0.97191	DYNC1LI1
0.084545	0.001448	-0.97292	HNRNPA3///HNRNPA3P1
-0.08909	0.001448	-0.97302	HSDL2
-0.07061	0.00145	-0.97435	NARG2
0.096667	0.001457	-0.97883	ZCCHC17
0.11	0.001463	-0.98278	HRSP12
-0.08879	0.001475	-0.99053	PLK1
0.098788	0.001475	-0.99071	MOCS2
-0.07879	0.001478	-0.99275	DOHH
-0.06091	0.001479	-0.99295	STAT1
0.089091	0.00148	-0.99396	ALG13
-0.05273	0.001481	-0.99439	TUFM
-0.06576	0.001486	-0.99746	FAF1
0.107273	0.001491	-1.0008	TRIM6-TRIM34///TRIM6///TRIM34
0.108788	0.001491	-1.00097	LOC401397
-0.06818	0.001492	-1.00176	PMVK
-0.06485	0.001493	-1.00234	U2AF2
0.108485	0.001499	-1.00606	PSMA3
-0.05545	0.001503	-1.00878	XPO6
0.133333	0.001504	-1.00941	COQ3
-0.07515	0.001507	-1.0111	KIAA0664
0.113939	0.001509	-1.01236	ZNF826P///ZNF826P
0.072121	0.001509	-1.01255	SLC25A36
0.106667	0.001513	-1.01522	INPP4B
0.068485	0.001514	-1.01563	ZBTB33
0.08	0.001516	-1.01666	TXNDC15
0.079091	0.001519	-1.01862	RBM34
-0.06424	0.001519	-1.01865	C19orf24
0.072121	0.00152	-1.01919	ZNF141
0.075152	0.001524	-1.0218	NIPAL2
-0.08091	0.001528	-1.02464	TOB2
0.073636	0.001531	-1.02629	TOPORS
-0.07061	0.001534	-1.02837	FAM109B
-0.10091	0.001537	-1.0302	PTMA
0.085455	0.001537	-1.0303	PPP3CC
0.060303	0.001559	-1.04353	NUMB
0.089091	0.001561	-1.04503	TAF11
0.068485	0.001564	-1.04675	H2AFZ
0.067879	0.001565	-1.04721	PDHX

-0.10303	0.001565	-1.04732	ZBTB46///SLC2A4RG
0.075152	0.001565	-1.04761	DGUOK
-0.10667	0.001567	-1.04842	HIST1H2BM
0.065152	0.001572	-1.0517	CNN3
0.072121	0.001575	-1.05379	RAD51C
0.146667	0.001576	-1.05409	NMI
-0.07061	0.001576	-1.05411	MIR26A2
-0.05606	0.001578	-1.05507	ADRBK1
0.092727	0.001578	-1.05511	MCEE
0.092121	0.001578	-1.05558	PRNP
-0.05667	0.001581	-1.05746	NUP205
-0.05	0.001592	-1.06406	CYFIP1
0.070606	0.001596	-1.06603	ANKRD10
0.079394	0.001599	-1.06791	AP1S2
-0.10242	0.001602	-1.0699	DEFB136
0.127879	0.001604	-1.07129	LOC728323///FBXO25
0.063939	0.001604	-1.07131	TMEM5
-0.06364	0.001605	-1.07189	ERO1L
0.079091	0.001608	-1.07321	RWDD2B
0.086364	0.001612	-1.07561	SLC25A33
-0.09667	0.001613	-1.07636	ARID1B
-0.05303	0.001619	-1.08006	EIF3B
-0.05636	0.001622	-1.0818	AP3D1
0.06	0.001626	-1.08384	HEXB
0.08697	0.001634	-1.08866	VEGFA
0.128485	0.001634	-1.08875	ZNF92
-0.1303	0.001646	-1.09594	SNORA64///RPS2
0.066364	0.001652	-1.09929	CALCOCO2
0.080909	0.001653	-1.09979	ZNF641
-0.07727	0.001653	-1.10018	PAK7
0.11303	0.001653	-1.10023	CEACAM1
0.090909	0.001654	-1.10037	POLR3GL
0.106667	0.00166	-1.10402	STAP1
-0.08121	0.001661	-1.10469	LRRC20
-0.07424	0.001665	-1.10698	BEND3
0.078182	0.001666	-1.10732	DUSP14
0.139394	0.001666	-1.10745	ZNF486
-0.07848	0.001667	-1.10781	H1FX
0.068182	0.00167	-1.10968	HMGCS1
0.081515	0.00168	-1.11555	ABHD4
0.066061	0.001681	-1.11603	NDUFS3
0.083636	0.001687	-1.11963	ALG8
0.074545	0.001688	-1.11982	LMAN1
-0.07909	0.001688	-1.12003	FLJ43860
-0.06697	0.001697	-1.12515	POLR1B
0.101515	0.001698	-1.12594	ZNF780B
0.07697	0.001702	-1.12812	C19orf42

0.080606	0.001704	-1.12916	CHRNA5
-0.07424	0.001705	-1.12956	IFNK
0.07	0.001707	-1.13056	ARL11
0.090303	0.001709	-1.13195	HSPA13
-0.06697	0.00171	-1.13252	AMN
0.083939	0.00171	-1.13279	SMNDC1
-0.06364	0.001712	-1.13373	DHCR7
0.072424	0.001715	-1.1353	KLHDC10
-0.05242	0.00172	-1.13828	PFKM
0.11	0.001723	-1.13983	VBP1
-0.06636	0.001725	-1.1407	HELLS
0.073333	0.001731	-1.14426	GTF2E2
0.087576	0.001732	-1.14481	TDO2
0.073636	0.001733	-1.14561	C7orf60
0.053333	0.001735	-1.14623	SRP9
0.086364	0.001736	-1.14699	SLC2A12///TBPL1
0.067879	0.001736	-1.14707	DUT
0.083939	0.001748	-1.15378	NUDT5
0.080909	0.001749	-1.1541	GLO1
0.069091	0.001751	-1.15537	SCIMP
0.068788	0.001753	-1.15639	MTHFS
-0.06364	0.001756	-1.15804	ADAMTS10
-0.08606	0.001759	-1.15951	PXN
-0.09727	0.001762	-1.16152	SOX17
-0.06242	0.001764	-1.16225	EMILIN2
0.049091	0.001772	-1.16673	KCMF1
-0.12242	0.001773	-1.1673	ODF3B
-0.06061	0.001774	-1.16772	KLF13
-0.06515	0.001777	-1.16932	PIEZO2
0.128182	0.001777	-1.16951	PHGDH
-0.05636	0.001779	-1.17032	CUL1
-0.05242	0.00178	-1.17101	SNRNP200
0.077576	0.00179	-1.17665	ADHFE1
0.063636	0.001793	-1.17813	TOMM22
0.065455	0.001803	-1.18333	CD58
-0.05485	0.001811	-1.1877	TYK2
-0.07152	0.001813	-1.18887	DNAJC30
-0.05848	0.001815	-1.18956	42248
0.09	0.001817	-1.19081	SNX4
0.094242	0.001819	-1.192	CHAC2
0.092121	0.00182	-1.19213	RAB2A
0.081515	0.001821	-1.19309	IL6ST
0.087273	0.001823	-1.19391	ESCO2
-0.04697	0.001823	-1.19396	EIF2AK3
-0.06394	0.001828	-1.19634	MYBPC3
0.112121	0.001828	-1.19649	LXN
0.045758	0.001828	-1.19661	H3F3AP4///H3F3A

0.152424	0.001832	-1.19869	LOC100134868
-0.07788	0.001834	-1.19967	FOXA3
0.128485	0.001836	-1.20063	GCA
-0.09697	0.001836	-1.20077	VCX3B///VCY1B///VCX3A///VCY
-0.09697	0.001836	-1.20077	VCX3B///VCY1B///VCX3A///VCY
0.136061	0.001838	-1.20162	NIPSNAP3A
0.081818	0.00184	-1.20265	IFI44
-0.08424	0.001842	-1.20379	NR2F1
-0.06303	0.001843	-1.20457	HGS
0.066667	0.00185	-1.20798	ACTR8
-0.09091	0.001852	-1.20919	IER2
-0.06212	0.001854	-1.21004	PIEZO1
0.058788	0.001855	-1.21071	BRK1
-0.06061	0.001857	-1.21162	REEP4
0.087879	0.001857	-1.21176	DNAJC7
0.168182	0.001858	-1.21243	PSAT1
-0.05818	0.001859	-1.21262	MAST2
0.069697	0.001862	-1.21409	PLIN2
-0.06485	0.001862	-1.2142	ST3GAL2
-0.08303	0.001864	-1.21529	FAM122A
0.060909	0.001864	-1.21548	RNF13
0.083333	0.001868	-1.21754	RPL9
-0.07485	0.001868	-1.21756	GRWD1
0.068485	0.001875	-1.22076	ST7L
-0.08394	0.001876	-1.22135	AXL
-0.10242	0.001878	-1.22239	PCDHA3
-0.09485	0.001885	-1.22603	POT1
0.057879	0.001885	-1.22606	CNPY2///PAN2
-0.08939	0.001887	-1.22705	LOC440337
0.073333	0.001889	-1.22833	DENR
-0.0703	0.001896	-1.2318	TPO
0.054848	0.001898	-1.23288	LOC100132832///DTX2P1-UPK3BP1-PMS2P11
0.121818	0.001901	-1.23421	ZNF681
0.059091	0.001903	-1.2354	MRPL42P5///C15orf57
0.065758	0.001907	-1.23726	PTAR1
0.051818	0.001907	-1.23737	ERH
-0.06788	0.001908	-1.23796	HPS3
0.08	0.00191	-1.2387	MPC1///MPC1
0.073939	0.001914	-1.24091	DIS3///PIBF1
-0.08242	0.00192	-1.24397	LHFPL4
0.061212	0.001921	-1.24428	LOC100289511///SRSF5
0.069091	0.001922	-1.24452	TMEM179B
-0.08515	0.001922	-1.24492	CDC42EP2
0.097576	0.001926	-1.24674	GIMAP2
-0.05788	0.001927	-1.24732	LOC100128573///ARHGEF18
0.08303	0.001934	-1.25091	IFT88
-0.06515	0.001941	-1.25404	SEC16A

-0.05606	0.001948	-1.2578	SLC22A1
-0.05636	0.001949	-1.25827	HELZ
0.109697	0.00195	-1.25841	TPMT
0.067273	0.001956	-1.26139	ZNF33B
0.092727	0.001959	-1.26294	PIGU
-0.13667	0.001964	-1.26542	LOC440518
0.065152	0.001971	-1.26881	C1orf74
0.062121	0.00198	-1.27346	ATP5J
0.081212	0.001983	-1.27458	EIF3F
0.067273	0.001984	-1.2752	CASP8AP2
-0.06818	0.001986	-1.27594	ADAM8
-0.08636	0.001991	-1.27871	C19orf77
0.051818	0.001996	-1.28116	APBB1IP
-0.05364	0.002	-1.28276	MYC
0.094242	0.002002	-1.2837	SPPL2A
0.073939	0.002002	-1.28387	EMC3
-0.06333	0.002002	-1.28409	SMEK2
0.058182	0.002004	-1.28472	TOR1A
0.090606	0.002007	-1.28625	HSBP1
-0.08485	0.002007	-1.28649	GABRD
0.077273	0.00201	-1.28751	COA6///SEC61A1
0.086061	0.002016	-1.29043	GSTM2
0.070303	0.002016	-1.29044	CCDC90A
0.08303	0.002016	-1.29053	RPL9
0.098788	0.002019	-1.29203	C1orf212
-0.0703	0.002026	-1.29513	OR5L2
0.086364	0.002026	-1.29534	LMBRD1
0.073939	0.002035	-1.29944	FAM98B
0.119697	0.002038	-1.30118	ZNF823
-0.06697	0.002039	-1.3014	FBXL5///FBXL5
0.125455	0.002041	-1.30218	C8orf44
-0.09939	0.002043	-1.3034	SESN3
0.098485	0.002045	-1.30432	RNPC3///AMY2B
-0.10242	0.002049	-1.30591	HIST1H3H///HIST1H2AI
-0.09758	0.00205	-1.30642	PCDHA13
0.163636	0.002052	-1.30733	C4orf27
0.181212	0.002056	-1.30928	CHAC1
0.059091	0.00206	-1.31129	CMTM1///CKLF///CKLF-CMTM1///CMTM1///CKLF
-0.05	0.002069	-1.31548	ADAR
-0.07303	0.002075	-1.31806	APLP1
-0.05788	0.002077	-1.31915	ASXL1
0.057879	0.002077	-1.31915	ZNF266
0.084242	0.002083	-1.32181	IGHV5-78
-0.06636	0.002084	-1.32246	ACACA
0.089091	0.002087	-1.32377	TRAF3IP3
0.085152	0.002088	-1.32411	NEK7
-0.10091	0.00209	-1.32516	HIST1H2BK///H2BFS///HIST1H2BE

0.096364	0.002097	-1.3282	LRRCC1
-0.06	0.002097	-1.32845	ABCB8
0.065152	0.002098	-1.32877	ARL2BP
-0.07636	0.002098	-1.32901	ATL3
0.073939	0.00211	-1.33445	TRMT12
-0.06788	0.002113	-1.33553	GGT6
0.072727	0.002115	-1.33641	RNF113A///NDUFA1
-0.07061	0.002118	-1.33784	EHMT1
0.074242	0.002124	-1.34061	GPX7
0.052424	0.002128	-1.34229	UFC1
0.096364	0.002133	-1.34455	STXBP3
0.085758	0.002133	-1.34465	GPR65
0.080303	0.002139	-1.34726	CCDC125
-0.07848	0.002139	-1.34745	SAMD9
0.091515	0.002147	-1.35101	P2RX4
0.074242	0.002155	-1.35433	EXOSC9
0.074545	0.002158	-1.35592	INO80C
0.094242	0.002162	-1.35772	RNF7
0.07	0.002165	-1.35897	MTF1
-0.05061	0.002165	-1.35912	RHOT2
-0.05606	0.002168	-1.36044	PRTN3
0.055152	0.002178	-1.36472	GNL3
0.100606	0.002178	-1.36475	RAP1B
0.080909	0.002191	-1.37049	RPF1
0.071515	0.002193	-1.3712	METTL9
0.080303	0.002194	-1.37176	UTP3
0.100606	0.0022	-1.37437	METTL7A
0.062727	0.0022	-1.37452	ZFAND6
0.067576	0.002201	-1.37488	C3orf38///CGGBP1
0.078485	0.002205	-1.37673	E2F6
0.062424	0.002209	-1.37844	CASP8
-0.13303	0.002215	-1.38106	GFOD1
-0.07091	0.002217	-1.3818	KIAA1967
-0.05606	0.002217	-1.38188	ACSL4
0.066364	0.002219	-1.38242	TEC
0.09	0.002222	-1.38401	RIPK2
0.08303	0.002228	-1.38629	RPL9
0.063333	0.002236	-1.39009	CXCR4
-0.13303	0.002238	-1.39074	HIST2H2BF///FCGR1B
-0.08727	0.002252	-1.39668	ARHGAP11A
0.069394	0.002259	-1.39961	HMBS
0.078182	0.002266	-1.40257	ORC4
0.077576	0.002267	-1.40296	HDAC8
0.103939	0.00227	-1.40429	PPIG
-0.09212	0.002271	-1.40476	WBSCR16
0.07303	0.002276	-1.40681	FBXO5
0.122424	0.002281	-1.40908	TOM1L1///COX11

0.112121	0.002282	-1.40956	KLHDC1
-0.05758	0.002282	-1.40964	PIK3R4
0.054545	0.002282	-1.40965	DTX2P1-UPK3BP1-PMS2P11///PMS2P5///PMS2P4
-0.07636	0.002283	-1.40994	GPR161
0.080909	0.002284	-1.4102	SH3BGRL
0.095758	0.002286	-1.4111	PSMA4
0.067879	0.002291	-1.41342	LOC100132832///PMS2P6///DTX2P1
0.069091	0.002292	-1.41364	PSMG2
0.072424	0.002297	-1.41569	SNX10
-0.08848	0.002308	-1.42029	XRCC3
0.090909	0.002315	-1.42317	TRIP11
-0.08848	0.002324	-1.42684	HIST1H2AL
-0.06667	0.002327	-1.42828	MBTPS1
0.050606	0.002328	-1.42862	FAM27E2///FAM27E3
0.072424	0.002331	-1.42966	FAM175B
-0.08576	0.002333	-1.43059	NKX6-2
0.068182	0.002335	-1.43163	FRA10AC1
-0.09061	0.002336	-1.43195	WNT7B
-0.05727	0.002337	-1.43245	RPS6KA3
0.064242	0.002341	-1.43378	HACL1
0.073939	0.002341	-1.43387	OSGEPL1
0.081212	0.002343	-1.43456	IQCK
0.081212	0.002343	-1.43456	RPL9
-0.09394	0.002346	-1.43605	GLTPD1
-0.06515	0.00235	-1.43757	AP1M2
-0.09455	0.002352	-1.43847	UBE2S
-0.08152	0.002352	-1.43849	STK11
-0.05939	0.002353	-1.43883	STIM1
-0.04606	0.002363	-1.44276	POLR1A///POLR1A
0.088788	0.002363	-1.44289	ZNF721///ABCA11P
-0.05273	0.002371	-1.44612	DICER1
0.061212	0.002376	-1.44811	PSMB9
0.061212	0.002376	-1.44811	PSMB9
0.061212	0.002376	-1.44811	PSMB9
0.056364	0.002387	-1.4527	KIAA1279
0.056667	0.002388	-1.453	PEA15
0.060303	0.002391	-1.45418	MMGT1
0.091515	0.002395	-1.45595	UGP2
-0.0703	0.002398	-1.45685	ZNF335
0.075758	0.002402	-1.45858	ITGB3BP
-0.08394	0.002406	-1.4601	EIF4E
0.07	0.002423	-1.46706	PPIL3
-0.05455	0.002427	-1.46835	MED12
0.066667	0.002432	-1.47066	FAM45A///FAM45B
-0.08818	0.002443	-1.47476	IL21R
0.075758	0.002446	-1.47612	DERL1
0.061818	0.002449	-1.47715	PPP2R2A

-0.11667	0.00245	-1.47759	NACC2
-0.07939	0.002451	-1.47784	PHLDB3
0.060303	0.002451	-1.47793	FECH
0.076364	0.002455	-1.47965	NUBP1
0.06	0.002457	-1.4802	EZH1
0.067576	0.002458	-1.48055	B4GALT6
-0.06636	0.002461	-1.48184	ABCC6P2///ABCC6P1///ABCC6
0.098788	0.002463	-1.48246	MIER3
-0.06727	0.002464	-1.48287	DLGAP1
0.084545	0.002476	-1.4876	BOD1L1
0.068485	0.002479	-1.48894	BBS2
0.108485	0.002481	-1.48946	CHURC1-FNTB///CHURC1
0.055152	0.002481	-1.48973	RPS3A
-0.06485	0.002487	-1.492	C22orf42
-0.05788	0.002496	-1.49522	TRRAP
0.054242	0.002499	-1.49674	LARP4
0.080303	0.002504	-1.49834	HCCS
0.067879	0.002515	-1.50272	LOC442075
0.112121	0.002522	-1.50548	OCLM
-0.05061	0.002527	-1.50721	LIMCH1
-0.04879	0.00253	-1.50851	PITRM1
-0.06515	0.002535	-1.51021	LEMD2
-0.08424	0.002537	-1.51117	TERT
-0.07576	0.00254	-1.51204	CIB3
-0.04848	0.002542	-1.51286	NPEPPS
0.071818	0.00255	-1.51574	MRPS22
-0.05091	0.002551	-1.51619	MCM5
0.105455	0.002552	-1.5168	RPAP3
0.106667	0.002564	-1.52125	GAPT
-0.07424	0.002566	-1.52201	NXNL2
0.056364	0.002567	-1.52227	SEC14L1P1///ALKBH3
0.070303	0.002569	-1.52313	RRN3P1///RRN3P2///RRN3
0.065758	0.00257	-1.52335	TMEM69
-0.07606	0.00257	-1.52335	CDX2
0.073333	0.002572	-1.52398	C4orf34
-0.07394	0.002572	-1.52417	IGFBP4
0.110606	0.002574	-1.52492	RAB13
-0.05	0.002578	-1.52655	SPRR3
-0.05667	0.002583	-1.52838	TNFRSF8
0.099394	0.002594	-1.53219	HNRNPA1L2///HNRNPA1
-0.06697	0.002594	-1.5322	ZGPAT
0.066364	0.002601	-1.53479	ZNF146
-0.07697	0.002604	-1.53587	SLC39A10
-0.06242	0.002614	-1.53951	POLR3G
-0.05697	0.002622	-1.54255	EEF1A2
0.141212	0.002622	-1.54272	SUMO1P3
0.080303	0.002626	-1.54412	ATPIF1

0.080303	0.002629	-1.5452	FCGR2C///FCGR2B
0.072121	0.002632	-1.54618	ACADM
0.08	0.002636	-1.54787	OCLN///LOC647859
-0.10697	0.002638	-1.54861	LOC654342
-0.06788	0.002642	-1.5499	LOC150197
0.070303	0.002642	-1.54997	ADD3
0.072121	0.002643	-1.55019	ST3GAL6
-0.04788	0.002644	-1.55067	MPRIP
-0.05545	0.002644	-1.55071	PREX1
0.055455	0.002644	-1.55071	WIPI1
-0.07848	0.002654	-1.55425	ARHGEF2
-0.04758	0.002662	-1.55704	ARFGAP2
-0.11455	0.002666	-1.55858	DNM1P46
-0.11455	0.002666	-1.55858	DNM1P46
-0.06576	0.002676	-1.562	PPP1R3F
-0.07788	0.002682	-1.56415	KCNJ18///KCNJ12
0.110606	0.002683	-1.56473	ZNF25
-0.05424	0.002686	-1.56571	ABCA1
0.06	0.002687	-1.56602	LDHB
0.090909	0.002689	-1.56675	LINC00665
0.107576	0.002689	-1.56688	IFT81
-0.06788	0.002692	-1.56791	HIST1H1E
0.073939	0.002694	-1.56868	CEP95
-0.07848	0.002698	-1.56994	HIST1H2AC
0.072121	0.002702	-1.57144	NDUFA8
0.069394	0.002702	-1.57145	FERMT2
0.087576	0.002706	-1.57279	ATL3
-0.05333	0.002709	-1.57379	ASCC3
-0.08	0.002715	-1.57592	SLC2A4
0.055455	0.00272	-1.57763	SF3A3
-0.06606	0.00272	-1.57771	STARD7
-0.09697	0.002723	-1.57865	KIAA1211
0.104545	0.002735	-1.58298	RPS14P3///RPS14
0.071515	0.002737	-1.58388	CWC27
0.094242	0.002742	-1.5855	CCDC125
0.095455	0.002746	-1.58673	REEP3
0.075152	0.002746	-1.58686	C7orf23///DMTF1
-0.04939	0.002746	-1.58701	NPAT
0.082727	0.002753	-1.5893	TMEM242
-0.0603	0.002753	-1.58941	KIFC1
0.065455	0.002757	-1.59059	UBE2W
-0.07485	0.002762	-1.59253	RBM47
0.053333	0.002764	-1.59304	RBBP4
0.061515	0.002765	-1.5935	TPST1///TPST1
0.069091	0.002776	-1.59737	TAX1BP1
-0.06667	0.002778	-1.598	PRKAR1B
0.09	0.002779	-1.59834	ZNF695

-0.09636	0.002782	-1.59942	BEND7
0.099091	0.00279	-1.6022	IFT57
0.076667	0.002793	-1.60309	RBM3
-0.07939	0.002795	-1.60385	OR1A1
0.08303	0.002797	-1.60468	ZNF26
-0.06818	0.002805	-1.60708	RNF213
0.066061	0.002805	-1.60739	HPRT1
-0.07182	0.002819	-1.61206	TRIO
-0.08697	0.002822	-1.6132	PLXNA1
0.072727	0.002825	-1.61396	HLTF
0.156364	0.002827	-1.61458	IL1A
0.06697	0.002837	-1.61794	SMAD1
0.089091	0.002838	-1.61837	LOC100130927///BPHL
0.084545	0.002838	-1.61851	H2BFXP///TMSB15B///H2BFM
0.078182	0.002845	-1.62066	ZNF256
0.081818	0.002848	-1.62184	ZNF852

Supplemental Table 2

The differentially expressed miRNAs (BH adjusted p < 0.05) between PEM treated and untreated LCLs. The top eight miRNAs were statistically significant after Bonferroni adjustment, and are highlighted in bold.

logFC	p-value	B-statistic	miRNA
**0.984431**	**3.53E-08**	**9.067583**	**hsa-miR-1244_st**
**0.742335**	**2.34E-07**	**7.340233**	**hsa-miR-494_st**
**0.44557**	**6.83E-07**	**6.337604**	**hsa-miR-1979_st**
**0.310382**	**3.97E-06**	**4.660077**	**hsa-miR-1826_st**
**0.769596**	**9.14E-06**	**3.853949**	**hsa-miR-1281_st**
**-0.31149**	**1.13E-05**	**3.651492**	**hsa-miR-202_st**
**-0.37295**	**1.14E-05**	**3.638406**	**hsa-miR-4284_st**
**-0.49043**	**1.70E-05**	**3.251481**	**hsa-miR-221-star_st**
-0.8822	6.51E-05	1.938847	hsa-miR-3172_st
-0.6379	9.48E-05	1.569842	hsa-miR-548a-3p_st
-0.43284	1.02E-04	1.493375	hsa-miR-1272_st
-0.33665	1.29E-04	1.266988	hsa-miR-15a_st
-0.40471	1.59E-04	1.062962	hsa-miR-3152_st
-0.21764	2.56E-04	0.592862	hsa-miR-142-5p_st
0.239945	3.03E-04	0.427568	hsa-miR-4270_st
-0.28655	3.27E-04	0.35261	hsa-miR-1910_st
-0.42143	3.29E-04	0.345954	hsa-miR-34a-star_st
-0.18062	3.78E-04	0.211917	hsa-miR-381_st
-0.30182	4.10E-04	0.131258	hsa-miR-450b-5p_st
0.297974	4.34E-04	0.075882	hsa-miR-1469_st
-0.40542	4.41E-04	0.058561	hsa-miR-32_st
0.649173	4.92E-04	-0.04704	hsa-miR-1825_st
-0.2357	5.13E-04	-0.08978	hsa-miR-1285_st
0.189846	5.43E-04	-0.14505	hsa-miR-4298_st
-0.4316	5.78E-04	-0.20634	hsa-miR-514b-5p_st
0.411552	7.24E-04	-0.42735	hsa-miR-4304_st
-0.23697	8.39E-04	-0.57208	hsa-miR-340_st
-0.19061	9.33E-04	-0.67548	hsa-miR-454_st
0.356262	1.11E-03	-0.84301	hsa-miR-103-as_st
-0.1806	1.17E-03	-0.89486	hsa-miR-548u_st
-0.14474	1.21E-03	-0.92817	hsa-miR-362-3p_st
0.131932	1.25E-03	-0.96127	hsa-miR-625_st
-0.22117	1.32E-03	-1.01387	hsa-let-7c_st
-0.45162	1.39E-03	-1.06657	hsa-miR-29c_st
0.490227	1.60E-03	-1.20241	hsa-miR-940_st
-0.27817	1.60E-03	-1.20558	hsa-miR-509-3-5p_st
-0.34216	1.63E-03	-1.22435	hsa-miR-509-5p_st
-0.28735	1.64E-03	-1.22946	hsa-miR-29b_st
-0.42906	1.68E-03	-1.25114	hsa-miR-3163_st

Supplemental Table 3

Correlated microRNAs and mRNAs after pemetrexed exposure.

miRNA	miRNA p-value	mRNA targets	ExpTarget Score*	mRNA p-value
miR-202	0.016	C4orf33	1.6	9.10E-06
		H3F3A	1.5	8.90E-06
		HSD17B11	1.1	3.50E-05
		HVCN1	1.7	6.10E-05
		KLHDC3	1.7	2.20E-05
		MTHFD2	1.5	1.80E-05
		PRIM1	1.0	1.30E-05
miR-494	3.47E-06	PSMD5	1.0	9.10E-05
		SUFU	1.3	4.10E-05

* All correlations between miRNA and mRNA have spearman r < -0.20

Supplemental Table 4

cis-acting eQTLs in human lung identified for the most highly differentially expressed genes (NA, missing rs identifier within GTEx annotation system).

rsid	chrom_pos	ENSEMBL_id	p-value	gene
rs9783081	1_247016787_G_A_b37	ENSG00000153207	1.97E-05	AHCTF1
rs7530860	1_247018184_A_G_b37	ENSG00000153207	2.39E-05	AHCTF1
rs1630829	1_247023866_C_T_b37	ENSG00000153207	2.64E-05	AHCTF1
rs1691251	1_247027779_T_C_b37	ENSG00000153207	1.65E-05	AHCTF1
rs34945773	1_247029063_T_TAA_b37	ENSG00000153207	1.13E-05	AHCTF1
NA	1_247029800_TAA_T_b37	ENSG00000153207	2.28E-05	AHCTF1
rs2800222	1_247031237_C_G_b37	ENSG00000153207	6.90E-06	AHCTF1
NA	1_247033377_CT_C_b37	ENSG00000153207	7.46E-07	AHCTF1
rs1613209	1_247035179_C_T_b37	ENSG00000153207	2.50E-06	AHCTF1
rs11585741	1_247045119_A_G_b37	ENSG00000153207	1.32E-06	AHCTF1
rs9729306	1_247045363_C_G_b37	ENSG00000153207	1.57E-06	AHCTF1
rs9729316	1_247045534_C_T_b37	ENSG00000153207	6.97E-06	AHCTF1
rs2642990	1_247048834_T_C_b37	ENSG00000153207	6.19E-06	AHCTF1
rs2642991	1_247049739_G_A_b37	ENSG00000153207	5.45E-07	AHCTF1
rs1472275	1_247054530_T_C_b37	ENSG00000153207	1.77E-06	AHCTF1
rs2800219	1_247055427_C_A_b37	ENSG00000153207	6.85E-07	AHCTF1
rs11436581	1_247056112_A_AT_b37	ENSG00000153207	6.45E-07	AHCTF1
rs2800220	1_247057814_T_C_b37	ENSG00000153207	1.17E-07	AHCTF1
rs2800221	1_247058705_C_T_b37	ENSG00000153207	6.85E-07	AHCTF1
rs1779964	1_247063209_G_A_b37	ENSG00000153207	3.50E-06	AHCTF1
rs1691252	1_247064149_T_C_b37	ENSG00000153207	3.73E-06	AHCTF1
rs1691254	1_247065075_A_G_b37	ENSG00000153207	6.85E-07	AHCTF1
rs1691255	1_247065472_A_G_b37	ENSG00000153207	6.61E-07	AHCTF1
NA	1_247066963_CCT_C_b37	ENSG00000153207	2.45E-06	AHCTF1
NA	1_247066964_CT_C_b37	ENSG00000153207	9.60E-08	AHCTF1
rs1691240	1_247067881_C_A_b37	ENSG00000153207	6.84E-07	AHCTF1
rs1691241	1_247068131_A_G_b37	ENSG00000153207	6.84E-07	AHCTF1
rs1612610	1_247068526_T_G_b37	ENSG00000153207	7.95E-07	AHCTF1
rs1691242	1_247068667_A_G_b37	ENSG00000153207	6.33E-07	AHCTF1
rs1779968	1_247069697_A_T_b37	ENSG00000153207	9.09E-07	AHCTF1
rs1779969	1_247069949_T_C_b37	ENSG00000153207	6.54E-07	AHCTF1
rs200680433	1_247074085_A_AAG_b37	ENSG00000153207	5.15E-07	AHCTF1
rs200148859	1_247074088_A_AGG_b37	ENSG00000153207	1.36E-06	AHCTF1
rs2800214	1_247077698_C_T_b37	ENSG00000153207	8.48E-07	AHCTF1
rs1039009	1_247080458_C_A_b37	ENSG00000153207	6.87E-07	AHCTF1
rs1039010	1_247081383_A_G_b37	ENSG00000153207	3.74E-06	AHCTF1
rs7542895	1_247083929_G_C_b37	ENSG00000153207	2.33E-06	AHCTF1
rs768197	1_247087232_A_C_b37	ENSG00000153207	6.70E-07	AHCTF1
rs2172796	1_247087618_A_G_b37	ENSG00000153207	6.70E-07	AHCTF1
rs2818888	1_247088863_A_G_b37	ENSG00000153207	6.48E-07	AHCTF1
rs2800215	1_247091897_A_G_b37	ENSG00000153207	3.44E-07	AHCTF1
rs2642975	1_247092186_A_T_b37	ENSG00000153207	2.61E-07	AHCTF1

rs2642977	1_247092378_T_C_b37	ENSG00000153207	2.47E-07	AHCTF1
rs1619941	1_247092780_T_A_b37	ENSG00000153207	3.81E-07	AHCTF1
rs6426188	1_247093410_C_A_b37	ENSG00000153207	2.70E-07	AHCTF1
rs55822102	1_247100806_C_G_b37	ENSG00000153207	4.32E-07	AHCTF1
rs2642980	1_247101213_T_G_b37	ENSG00000153207	8.45E-06	AHCTF1
rs10924873	1_247102892_G_A_b37	ENSG00000153207	8.06E-06	AHCTF1
rs12075371	1_247103048_T_G_b37	ENSG00000153207	7.07E-07	AHCTF1
rs56217639	1_247103215_A_T_b37	ENSG00000153207	1.09E-05	AHCTF1
rs12030390	1_247103478_G_A_b37	ENSG00000153207	2.91E-06	AHCTF1
NA	1_247103612_AT_A_b37	ENSG00000153207	8.46E-06	AHCTF1
rs28501970	1_247103945_A_G_b37	ENSG00000153207	4.73E-06	AHCTF1
rs7410910	1_247104023_G_A_b37	ENSG00000153207	4.76E-07	AHCTF1
rs7417381	1_247104047_A_G_b37	ENSG00000153207	2.05E-08	AHCTF1
rs6426189	1_247104295_C_G_b37	ENSG00000153207	2.95E-06	AHCTF1
rs7538195	1_247104606_C_T_b37	ENSG00000153207	7.10E-06	AHCTF1
rs1691234	1_247106126_A_G_b37	ENSG00000153207	2.56E-06	AHCTF1
rs1691233	1_247106406_T_G_b37	ENSG00000153207	2.55E-06	AHCTF1
rs1691230	1_247107000_A_G_b37	ENSG00000153207	2.56E-06	AHCTF1
rs1691229	1_247107152_G_A_b37	ENSG00000153207	2.56E-06	AHCTF1
rs1779970	1_247107350_C_T_b37	ENSG00000153207	1.07E-05	AHCTF1
NA	1_247108141_CG_C_b37	ENSG00000153207	3.35E-06	AHCTF1
rs12750906	1_247108504_T_C_b37	ENSG00000153207	5.67E-07	AHCTF1
rs2642982	1_247109014_G_C_b37	ENSG00000153207	2.46E-06	AHCTF1
rs2642983	1_247109267_A_C_b37	ENSG00000153207	2.45E-06	AHCTF1
rs2818886	1_247109408_G_A_b37	ENSG00000153207	2.58E-06	AHCTF1
rs2800217	1_247109468_C_T_b37	ENSG00000153207	1.85E-07	AHCTF1
rs2800216	1_247109609_C_A_b37	ENSG00000153207	4.84E-08	AHCTF1
rs2818887	1_247109656_A_G_b37	ENSG00000153207	4.84E-08	AHCTF1
rs12722828	1_247110085_T_C_b37	ENSG00000153207	2.37E-06	AHCTF1
rs12740976	1_247110240_A_G_b37	ENSG00000153207	1.59E-07	AHCTF1
rs6666435	1_247110260_A_G_b37	ENSG00000153207	2.35E-07	AHCTF1
rs1779962	1_247111062_T_C_b37	ENSG00000153207	4.77E-08	AHCTF1
rs1779961	1_247111521_A_G_b37	ENSG00000153207	2.12E-06	AHCTF1
rs1691244	1_247111855_C_A_b37	ENSG00000153207	1.74E-06	AHCTF1
rs1779960	1_247111860_A_G_b37	ENSG00000153207	1.85E-06	AHCTF1
rs1691245	1_247112207_C_T_b37	ENSG00000153207	3.85E-06	AHCTF1
rs1779959	1_247112269_G_C_b37	ENSG00000153207	8.87E-07	AHCTF1
rs1779958	1_247112298_G_C_b37	ENSG00000153207	8.85E-07	AHCTF1
rs1691246	1_247112320_A_G_b37	ENSG00000153207	2.06E-06	AHCTF1
rs1779985	1_247129945_A_G_b37	ENSG00000153207	3.38E-05	AHCTF1
rs2818890	1_247135846_T_C_b37	ENSG00000153207	3.58E-05	AHCTF1
NA	1_247137454_AC_A_b37	ENSG00000153207	2.68E-05	AHCTF1
rs9991981	4_129713511_G_A_b37	ENSG0000015147C	2.55E-05	C4orf33
rs6834108	4_129725894_T_C_b37	ENSG0000015147C	2.16E-05	C4orf33
rs1841260	4_129726784_G_A_b37	ENSG0000015147C	2.83E-05	C4orf33
rs1841259	4_129726915_A_G_b37	ENSG0000015147C	2.49E-05	C4orf33
rs6817004	4_129727677_C_T_b37	ENSG0000015147C	2.28E-05	C4orf33

rs10008927	4_129729239_G_A_b37	ENSG0000015147C	5.89E-08	C4orf33
rs13143771	4_129729412_C_T_b37	ENSG0000015147C	2.10E-05	C4orf33
rs2217023	4_129732099_C_G_b37	ENSG0000015147C	4.11E-07	C4orf33
rs200881480	4_129732799_G_GT_b37	ENSG0000015147C	3.51E-07	C4orf33
rs62317870	4_129733125_C_T_b37	ENSG0000015147C	5.72E-06	C4orf33
rs4975264	4_129738176_T_G_b37	ENSG0000015147C	3.07E-05	C4orf33
rs4975265	4_129738210_T_C_b37	ENSG0000015147C	2.07E-08	C4orf33
rs4975266	4_129738328_T_C_b37	ENSG0000015147C	2.36E-05	C4orf33
rs10529563	4_129738454_C_CTGAG_b37	ENSG0000015147C	7.83E-08	C4orf33
rs7687562	4_129739899_T_C_b37	ENSG0000015147C	3.14E-08	C4orf33
rs4975267	4_129744992_G_A_b37	ENSG0000015147C	3.35E-08	C4orf33
rs4975269	4_129752134_G_T_b37	ENSG0000015147C	3.15E-08	C4orf33
rs13120702	4_129755980_A_G_b37	ENSG0000015147C	7.45E-08	C4orf33
rs6857659	4_129763370_G_T_b37	ENSG0000015147C	3.81E-07	C4orf33
rs11724119	4_129768342_A_G_b37	ENSG0000015147C	7.07E-05	C4orf33
rs12512715	4_129772286_G_A_b37	ENSG0000015147C	7.50E-08	C4orf33
rs4975274	4_129778076_T_C_b37	ENSG0000015147C	9.39E-09	C4orf33
rs2162126	4_129779076_T_C_b37	ENSG0000015147C	1.16E-07	C4orf33
NA	4_129796194_CAAATT_C_b37	ENSG0000015147C	2.02E-08	C4orf33
rs318555	4_129798627_T_G_b37	ENSG0000015147C	2.34E-05	C4orf33
rs318556	4_129799974_C_G_b37	ENSG0000015147C	1.30E-05	C4orf33
rs318502	4_129801495_A_C_b37	ENSG0000015147C	1.60E-08	C4orf33
rs1648062	4_129801732_A_C_b37	ENSG0000015147C	5.24E-06	C4orf33
rs318501	4_129802948_G_A_b37	ENSG0000015147C	1.59E-08	C4orf33
rs318500	4_129804727_G_A_b37	ENSG0000015147C	1.59E-08	C4orf33
rs167618	4_129805716_G_A_b37	ENSG0000015147C	7.10E-06	C4orf33
rs318503	4_129807236_G_T_b37	ENSG0000015147C	3.53E-08	C4orf33
rs318504	4_129808206_G_A_b37	ENSG0000015147C	3.93E-05	C4orf33
rs318505	4_129808936_G_T_b37	ENSG0000015147C	2.59E-06	C4orf33
rs170325	4_129809378_T_G_b37	ENSG0000015147C	8.30E-06	C4orf33
rs318513	4_129816821_C_T_b37	ENSG0000015147C	1.16E-09	C4orf33
rs318517	4_129817440_C_T_b37	ENSG0000015147C	1.03E-09	C4orf33
rs318521	4_129818192_T_C_b37	ENSG0000015147C	3.13E-09	C4orf33
rs1668289	4_129827039_T_C_b37	ENSG0000015147C	3.13E-09	C4orf33
rs3113407	4_129827145_C_A_b37	ENSG0000015147C	4.66E-08	C4orf33
NA	4_129827850_TAGAA_T_b37	ENSG0000015147C	5.23E-09	C4orf33
rs318536	4_129831257_C_T_b37	ENSG0000015147C	3.13E-09	C4orf33
rs318543	4_129836255_T_C_b37	ENSG0000015147C	1.03E-09	C4orf33
rs1674974	4_129838452_T_A_b37	ENSG0000015147C	4.24E-09	C4orf33
rs318552	4_129841122_G_A_b37	ENSG0000015147C	1.03E-09	C4orf33
rs4052	4_129845927_G_A_b37	ENSG0000015147C	3.85E-07	C4orf33
rs11731189	4_129853155_T_C_b37	ENSG0000015147C	2.19E-07	C4orf33
rs10010659	4_129856847_T_A_b37	ENSG0000015147C	7.28E-10	C4orf33
rs10032000	4_129863157_T_C_b37	ENSG0000015147C	2.25E-09	C4orf33
rs66921209	4_129868556_T_C_b37	ENSG0000015147C	8.16E-10	C4orf33
rs10028974	4_129871442_C_A_b37	ENSG0000015147C	2.83E-07	C4orf33
rs28711799	4_129874496_T_A_b37	ENSG0000015147C	1.87E-08	C4orf33

rs10002332	4_129875301_A_G_b37	ENSG0000015147C	1.05E-10	C4orf33
rs6824219	4_129876836_G_A_b37	ENSG0000015147C	1.86E-08	C4orf33
rs35006492	4_129878342_G_A_b37	ENSG0000015147C	1.26E-10	C4orf33
rs12171333	4_129879711_G_A_b37	ENSG0000015147C	8.81E-10	C4orf33
rs11934740	4_129882748_C_T_b37	ENSG0000015147C	1.84E-08	C4orf33
rs2198042	4_129885499_C_A_b37	ENSG0000015147C	2.13E-09	C4orf33
rs11098994	4_129888651_C_G_b37	ENSG0000015147C	1.44E-07	C4orf33
rs57501568	4_129888787_G_T_b37	ENSG0000015147C	5.30E-09	C4orf33
rs7655841	4_129890790_T_C_b37	ENSG0000015147C	8.00E-10	C4orf33
rs2034498	4_129892905_C_T_b37	ENSG0000015147C	5.47E-10	C4orf33
rs4394010	4_129894649_G_A_b37	ENSG0000015147C	1.43E-10	C4orf33
rs6812361	4_129899758_G_A_b37	ENSG0000015147C	1.28E-08	C4orf33
rs2597837	4_129904050_T_C_b37	ENSG0000015147C	2.98E-08	C4orf33
rs2597836	4_129904130_A_G_b37	ENSG0000015147C	4.68E-11	C4orf33
rs1376202	4_129907917_T_C_b37	ENSG0000015147C	1.44E-07	C4orf33
NA	4_129911111_TA_T_b37	ENSG0000015147C	1.90E-10	C4orf33
rs1596963	4_129912966_T_A_b37	ENSG0000015147C	1.63E-08	C4orf33
rs280601	4_129914268_C_T_b37	ENSG0000015147C	2.98E-08	C4orf33
rs280603	4_129915063_C_A_b37	ENSG0000015147C	1.01E-10	C4orf33
rs3099899	4_129917368_A_G_b37	ENSG0000015147C	1.05E-10	C4orf33
rs280604	4_129921170_G_T_b37	ENSG0000015147C	2.74E-11	C4orf33
rs4466058	4_129923879_T_C_b37	ENSG0000015147C	1.47E-10	C4orf33
rs3113487	4_129924977_C_A_b37	ENSG0000015147C	2.35E-07	C4orf33
rs142612043	4_129926746_T_C_b37	ENSG0000015147C	1.61E-05	C4orf33
rs55865936	4_129927010_T_C_b37	ENSG0000015147C	4.59E-06	C4orf33
rs3113488	4_129929138_C_T_b37	ENSG0000015147C	1.42E-08	C4orf33
rs150320461	4_129937502_G_GTA_b37	ENSG0000015147C	1.54E-06	C4orf33
rs280594	4_129942287_T_C_b37	ENSG0000015147C	1.74E-08	C4orf33
rs280596	4_129942678_C_T_b37	ENSG0000015147C	1.32E-08	C4orf33
rs280597	4_129944209_C_T_b37	ENSG0000015147C	1.09E-07	C4orf33
rs144068810	4_129945525_G_A_b37	ENSG0000015147C	2.49E-10	C4orf33
rs139907008	4_129948304_C_T_b37	ENSG0000015147C	3.17E-08	C4orf33
rs143397855	4_129954412_T_C_b37	ENSG0000015147C	5.23E-06	C4orf33
rs3113489	4_129961179_A_G_b37	ENSG0000015147C	6.23E-10	C4orf33
rs3099903	4_129962072_T_C_b37	ENSG0000015147C	1.13E-07	C4orf33
rs3099902	4_129963504_A_T_b37	ENSG0000015147C	1.34E-08	C4orf33
rs3113490	4_129965671_T_C_b37	ENSG0000015147C	8.70E-08	C4orf33
rs1374491	4_129968621_C_T_b37	ENSG0000015147C	3.33E-10	C4orf33
rs6534707	4_129970673_C_T_b37	ENSG0000015147C	1.59E-09	C4orf33
rs115222555	4_129971733_G_A_b37	ENSG0000015147C	1.96E-05	C4orf33
rs143409046	4_129972741_A_C_b37	ENSG0000015147C	1.97E-05	C4orf33
rs13109003	4_129975969_T_C_b37	ENSG0000015147C	2.33E-10	C4orf33
rs62316971	4_129976373_C_G_b37	ENSG0000015147C	1.33E-08	C4orf33
rs2083634	4_129976478_T_C_b37	ENSG0000015147C	2.30E-10	C4orf33
rs7671508	4_129977667_G_T_b37	ENSG0000015147C	6.34E-08	C4orf33
rs7654405	4_129977906_T_A_b37	ENSG0000015147C	1.33E-08	C4orf33
rs2118045	4_129979793_C_A_b37	ENSG0000015147C	1.10E-07	C4orf33

rs4975193	4_129980630_C_T_b37	ENSG0000015147(	3.82E-11	C4orf33
rs4975194	4_129982377_A_G_b37	ENSG0000015147(	3.80E-11	C4orf33
rs2655311	4_129986299_C_A_b37	ENSG0000015147(	1.33E-08	C4orf33
rs1699384	4_129987071_A_C_b37	ENSG0000015147(	8.39E-10	C4orf33
rs1757938	4_129987759_C_T_b37	ENSG0000015147(	1.58E-08	C4orf33
rs1854774	4_129988405_A_C_b37	ENSG0000015147(	7.75E-08	C4orf33
rs1699385	4_129990164_T_A_b37	ENSG0000015147(	3.76E-11	C4orf33
rs2777823	4_129990743_T_C_b37	ENSG0000015147(	1.12E-08	C4orf33
rs58689608	4_129991464_G_T_b37	ENSG0000015147(	3.76E-11	C4orf33
rs1838911	4_129991636_T_G_b37	ENSG0000015147(	1.09E-07	C4orf33
rs62316978	4_129992058_T_G_b37	ENSG0000015147(	1.08E-07	C4orf33
rs1699379	4_129992337_A_G_b37	ENSG0000015147(	3.49E-11	C4orf33
rs2592948	4_129994690_G_A_b37	ENSG0000015147(	1.33E-08	C4orf33
rs1757937	4_129995171_G_A_b37	ENSG0000015147(	1.09E-07	C4orf33
rs796886	4_129996889_C_T_b37	ENSG0000015147(	1.33E-08	C4orf33
rs789982	4_129999071_T_C_b37	ENSG0000015147(	4.82E-07	C4orf33
rs1757942	4_130001593_C_T_b37	ENSG0000015147(	1.33E-08	C4orf33
rs1699388	4_130003237_T_C_b37	ENSG0000015147(	3.76E-11	C4orf33
rs1699389	4_130004079_A_G_b37	ENSG0000015147(	3.76E-11	C4orf33
rs1699391	4_130005096_G_A_b37	ENSG0000015147(	3.80E-11	C4orf33
rs1854773	4_130008092_T_C_b37	ENSG0000015147(	7.52E-11	C4orf33
rs2777822	4_130009131_T_A_b37	ENSG0000015147(	3.65E-07	C4orf33
rs1757936	4_130011891_G_A_b37	ENSG0000015147(	6.34E-08	C4orf33
rs67225319	4_130015456_A_AT_b37	ENSG0000015147(	7.81E-06	C4orf33
rs2655310	4_130017919_G_A_b37	ENSG0000015147(	1.66E-11	C4orf33
rs1756012	4_130018971_T_C_b37	ENSG0000015147(	1.76E-10	C4orf33
rs1699394	4_130019312_C_T_b37	ENSG0000015147(	2.24E-10	C4orf33
rs1699393	4_130019352_T_G_b37	ENSG0000015147(	9.01E-10	C4orf33
rs1757924	4_130019599_G_T_b37	ENSG0000015147(	3.77E-10	C4orf33
rs1756011	4_130019663_A_G_b37	ENSG0000015147(	8.86E-08	C4orf33
rs34638497	4_130020042_A_AG_b37	ENSG0000015147(	1.77E-11	C4orf33
rs1757925	4_130020791_T_C_b37	ENSG0000015147(	1.97E-11	C4orf33
rs1757926	4_130021892_A_G_b37	ENSG0000015147(	8.88E-08	C4orf33
rs1757927	4_130022069_C_T_b37	ENSG0000015147(	1.99E-11	C4orf33
rs1757928	4_130022161_C_A_b37	ENSG0000015147(	1.99E-11	C4orf33
rs1757929	4_130022200_A_G_b37	ENSG0000015147(	8.88E-08	C4orf33
rs1699392	4_130022347_C_T_b37	ENSG0000015147(	1.99E-11	C4orf33
rs1757930	4_130022356_G_A_b37	ENSG0000015147(	1.67E-10	C4orf33
rs3105369	4_130022448_G_A_b37	ENSG0000015147(	1.99E-11	C4orf33
rs13130762	4_130022875_C_A_b37	ENSG0000015147(	1.36E-11	C4orf33
rs1757935	4_130023759_A_T_b37	ENSG0000015147(	1.99E-11	C4orf33
rs201427408	4_130024725_T_TTA_b37	ENSG0000015147(	2.21E-11	C4orf33
rs111792441	4_130024726_T_TAC_b37	ENSG0000015147(	4.81E-11	C4orf33
4:130024727	4_130024727_A_ACC_b37	ENSG0000015147(	3.07E-11	C4orf33
rs1030831	4_130025037_T_G_b37	ENSG0000015147(	1.99E-11	C4orf33
rs1037147	4_130025873_T_A_b37	ENSG0000015147(	1.99E-11	C4orf33
rs56141111	4_130025913_G_GT_b37	ENSG0000015147(	1.24E-11	C4orf33

rs189236	4_130028412_C_T_b37	ENSG0000015147C	4.49E-08	C4orf33
rs337279	4_130028426_G_T_b37	ENSG0000015147C	4.48E-08	C4orf33
rs337278	4_130029879_A_C_b37	ENSG0000015147C	2.32E-11	C4orf33
rs337277	4_130030652_A_G_b37	ENSG0000015147C	3.23E-10	C4orf33
rs337276	4_130030944_C_A_b37	ENSG0000015147C	1.38E-05	C4orf33
rs1699387	4_130031498_T_G_b37	ENSG0000015147C	3.41E-10	C4orf33
rs337263	4_130033665_C_T_b37	ENSG0000015147C	6.33E-11	C4orf33
NA	4_130034197_AAAC_A_b37	ENSG0000015147C	6.14E-06	C4orf33
rs1699380	4_130034581_T_C_b37	ENSG0000015147C	2.67E-10	C4orf33
NA	4_130034753_AT_A_b37	ENSG0000015147C	1.69E-10	C4orf33
4:130034935	4_130034935_A_AAC_b37	ENSG0000015147C	7.53E-06	C4orf33
rs189235	4_130035370_T_A_b37	ENSG0000015147C	2.67E-10	C4orf33
rs426521	4_130035755_G_A_b37	ENSG0000015147C	2.67E-10	C4orf33
rs445294	4_130035889_C_T_b37	ENSG0000015147C	2.67E-10	C4orf33
rs487233	4_130036142_A_G_b37	ENSG0000015147C	2.53E-10	C4orf33
rs389376	4_130036179_A_C_b37	ENSG0000015147C	2.54E-10	C4orf33
rs487501	4_130036191_T_C_b37	ENSG0000015147C	2.54E-10	C4orf33
rs390260	4_130036591_C_T_b37	ENSG0000015147C	2.75E-10	C4orf33
rs391396	4_130037653_G_A_b37	ENSG0000015147C	2.87E-10	C4orf33
rs474794	4_130038674_T_C_b37	ENSG0000015147C	3.25E-10	C4orf33
rs474667	4_130038714_T_C_b37	ENSG0000015147C	3.44E-10	C4orf33
rs1709419	4_130038913_T_C_b37	ENSG0000015147C	4.65E-10	C4orf33
rs530686	4_130039884_A_G_b37	ENSG0000015147C	1.81E-05	C4orf33
rs504968	4_130040437_T_C_b37	ENSG0000015147C	4.71E-09	C4orf33
rs337270	4_130042232_A_C_b37	ENSG0000015147C	7.04E-10	C4orf33
rs139037552	4_130043436_C_T_b37	ENSG0000015147C	3.94E-09	C4orf33
rs140828529	4_130044425_A_G_b37	ENSG0000015147C	1.92E-09	C4orf33
rs517659	4_130045321_G_C_b37	ENSG0000015147C	1.11E-09	C4orf33
rs576492	4_130045592_T_G_b37	ENSG0000015147C	8.52E-10	C4orf33
rs546956	4_130046216_A_T_b37	ENSG0000015147C	3.31E-08	C4orf33
rs202215815	4_130046217_T_G_b37	ENSG0000015147C	3.31E-08	C4orf33
rs202215815	4_130046217_T_TG_b37	ENSG0000015147C	8.30E-08	C4orf33
rs395864	4_130050091_A_G_b37	ENSG0000015147C	1.77E-09	C4orf33
rs337275	4_130054469_T_C_b37	ENSG0000015147C	3.96E-09	C4orf33
rs337274	4_130056680_T_G_b37	ENSG0000015147C	5.77E-09	C4orf33
rs17014243	4_130057766_C_T_b37	ENSG0000015147C	6.18E-05	C4orf33
rs1757923	4_130058670_T_C_b37	ENSG0000015147C	1.05E-08	C4orf33
rs1709421	4_130058979_G_A_b37	ENSG0000015147C	4.72E-08	C4orf33
rs1709422	4_130064878_C_A_b37	ENSG0000015147C	8.31E-06	C4orf33
rs771	4_130070886_C_T_b37	ENSG0000015147C	3.69E-05	C4orf33
rs17049	4_130070947_C_T_b37	ENSG0000015147C	5.61E-05	C4orf33
rs4527495	4_130071613_A_C_b37	ENSG0000015147C	3.86E-05	C4orf33
rs4513585	4_130072580_G_A_b37	ENSG0000015147C	1.33E-05	C4orf33
rs12511871	4_130072732_G_A_b37	ENSG0000015147C	6.30E-05	C4orf33
rs13123966	4_130074947_G_A_b37	ENSG0000015147C	7.28E-05	C4orf33
rs11933725	4_130075104_A_G_b37	ENSG0000015147C	7.28E-05	C4orf33
rs4975196	4_130101685_G_A_b37	ENSG0000015147C	1.73E-05	C4orf33

rs1112957	4_130114428_A_G_b37	ENSG0000015147(	2.08E-05	C4orf33
rs144883505	4_130120897_G_A_b37	ENSG0000015147(	3.28E-05	C4orf33
rs6857889	4_130122117_G_A_b37	ENSG0000015147(	3.22E-05	C4orf33
rs9366005	6_166730466_A_G_b37	ENSG0000019881{	1.39E-08	SFT2D1
rs9356476	6_166730499_A_G_b37	ENSG0000019881{	1.46E-08	SFT2D1
rs9366006	6_166730737_A_G_b37	ENSG0000019881{	9.12E-09	SFT2D1
rs9366007	6_166732338_T_C_b37	ENSG0000019881{	3.55E-09	SFT2D1
rs4598048	6_166732624_C_G_b37	ENSG0000019881{	7.81E-09	SFT2D1
rs9355572	6_166732650_A_G_b37	ENSG0000019881{	6.20E-09	SFT2D1
rs6918901	6_166734193_T_C_b37	ENSG0000019881{	1.07E-08	SFT2D1
rs4710041	6_166737980_G_A_b37	ENSG0000019881{	5.27E-07	SFT2D1
rs10806847	6_166739684_G_A_b37	ENSG0000019881{	7.06E-10	SFT2D1
rs4470829	6_166741291_T_A_b37	ENSG0000019881{	5.62E-09	SFT2D1
rs7757549	6_166744262_C_G_b37	ENSG0000019881{	2.16E-09	SFT2D1
rs9459654	6_166744444_T_C_b37	ENSG0000019881{	5.01E-10	SFT2D1
rs6927168	6_166745394_C_G_b37	ENSG0000019881{	5.60E-10	SFT2D1
rs9356478	6_166751416_C_T_b37	ENSG0000019881{	6.75E-10	SFT2D1
rs4709112	6_166751839_A_G_b37	ENSG0000019881{	5.26E-09	SFT2D1
rs9356479	6_166755480_A_G_b37	ENSG0000019881{	6.40E-08	SFT2D1
rs10862	6_166755975_T_C_b37	ENSG0000019881{	2.60E-10	SFT2D1
rs11168	6_166755979_A_C_b37	ENSG0000019881{	2.60E-10	SFT2D1
rs6909951	6_166758198_A_G_b37	ENSG0000019881{	6.65E-10	SFT2D1
rs2343590	6_166759622_C_G_b37	ENSG0000019881{	4.34E-10	SFT2D1
rs10808113	7_77197303_T_C_b37	ENSG00000135211	3.00E-05	TMEM60
rs4729554	7_77236976_G_C_b37	ENSG00000135211	2.88E-05	TMEM60
rs4727453	7_77292821_A_G_b37	ENSG00000135211	2.41E-05	TMEM60
rs6955558	7_77306266_A_G_b37	ENSG00000135211	7.53E-07	TMEM60
rs6955747	7_77306394_A_C_b37	ENSG00000135211	7.84E-07	TMEM60
rs7801062	7_77312474_C_T_b37	ENSG00000135211	6.10E-06	TMEM60
7:77314619	7_77314619_T_TCA_b37	ENSG00000135211	1.23E-05	TMEM60
rs4727463	7_77323798_A_G_b37	ENSG00000135211	1.78E-06	TMEM60
rs13240016	7_77324475_A_G_b37	ENSG00000135211	2.23E-06	TMEM60
rs13225229	7_77324495_G_A_b37	ENSG00000135211	2.03E-06	TMEM60
rs13243012	7_77324582_T_C_b37	ENSG00000135211	1.44E-06	TMEM60
rs3750128	7_77326410_T_C_b37	ENSG00000135211	4.54E-06	TMEM60
rs10235679	7_77331485_G_A_b37	ENSG00000135211	2.51E-06	TMEM60
rs10239156	7_77332058_G_C_b37	ENSG00000135211	2.67E-06	TMEM60
rs11760252	7_77335673_C_G_b37	ENSG00000135211	3.26E-06	TMEM60
rs12539065	7_77337784_C_T_b37	ENSG00000135211	3.66E-06	TMEM60
rs6966632	7_77342199_G_T_b37	ENSG00000135211	2.95E-06	TMEM60
rs61364263	7_77346098_A_G_b37	ENSG00000135211	4.24E-06	TMEM60
rs6946914	7_77346469_T_G_b37	ENSG00000135211	2.78E-06	TMEM60
rs6465779	7_77349237_G_A_b37	ENSG00000135211	2.80E-06	TMEM60
rs4729643	7_77349390_C_T_b37	ENSG00000135211	2.79E-06	TMEM60
rs6967914	7_77350198_T_C_b37	ENSG00000135211	4.06E-06	TMEM60
rs7782728	7_77350833_C_T_b37	ENSG00000135211	1.15E-05	TMEM60
rs7783032	7_77351705_G_T_b37	ENSG00000135211	2.81E-06	TMEM60

rs9691803	7_77352700_A_G_b37	ENSG00000135211	2.81E-06	TMEM60
rs55980640	7_77354028_C_T_b37	ENSG00000135211	3.64E-05	TMEM60
rs7798245	7_77354714_G_A_b37	ENSG00000135211	6.04E-06	TMEM60
rs7804910	7_77356759_T_A_b37	ENSG00000135211	3.44E-06	TMEM60
rs10264590	7_77357748_A_G_b37	ENSG00000135211	3.46E-06	TMEM60
rs7789173	7_77360623_T_C_b37	ENSG00000135211	3.45E-06	TMEM60
rs6465789	7_77364536_A_C_b37	ENSG00000135211	2.07E-06	TMEM60
rs3764824	7_77365770_A_G_b37	ENSG00000135211	2.27E-06	TMEM60
rs10258826	7_77366177_A_G_b37	ENSG00000135211	3.08E-06	TMEM60
rs4729668	7_77368080_G_C_b37	ENSG00000135211	2.35E-05	TMEM60
rs2040953	7_77370478_G_A_b37	ENSG00000135211	5.12E-06	TMEM60
rs10441228	7_77370662_A_G_b37	ENSG00000135211	2.95E-06	TMEM60
NA	7_77372778_TTTG_T_b37	ENSG00000135211	3.95E-06	TMEM60
NA	7_77372779_TTG_T_b37	ENSG00000135211	3.67E-06	TMEM60
rs6945958	7_77372906_C_T_b37	ENSG00000135211	1.91E-06	TMEM60
rs6976144	7_77374804_A_G_b37	ENSG00000135211	6.57E-07	TMEM60
rs6956726	7_77374846_C_T_b37	ENSG00000135211	1.30E-06	TMEM60
rs6976946	7_77375311_A_G_b37	ENSG00000135211	1.88E-06	TMEM60
rs13233727	7_77375396_A_G_b37	ENSG00000135211	1.88E-06	TMEM60
rs6465792	7_77375468_C_T_b37	ENSG00000135211	1.88E-06	TMEM60
rs6465793	7_77375525_T_G_b37	ENSG00000135211	1.88E-06	TMEM60
rs6465794	7_77376005_T_C_b37	ENSG00000135211	1.88E-06	TMEM60
rs10233055	7_77376322_G_T_b37	ENSG00000135211	1.53E-06	TMEM60
rs28470505	7_77377236_A_G_b37	ENSG00000135211	1.87E-06	TMEM60
rs10225586	7_77381372_C_G_b37	ENSG00000135211	1.85E-06	TMEM60
7:77381734	7_77381734_T_TCA_b37	ENSG00000135211	4.53E-06	TMEM60
rs9986707	7_77382263_C_T_b37	ENSG00000135211	1.19E-06	TMEM60
rs9986891	7_77382288_G_A_b37	ENSG00000135211	1.84E-06	TMEM60
rs9986830	7_77382350_T_C_b37	ENSG00000135211	1.84E-06	TMEM60
rs4727492	7_77391469_A_G_b37	ENSG00000135211	2.46E-06	TMEM60
rs6952760	7_77392267_G_C_b37	ENSG00000135211	3.51E-06	TMEM60
rs10272202	7_77396235_G_A_b37	ENSG00000135211	3.28E-06	TMEM60
rs6952502	7_77397379_C_T_b37	ENSG00000135211	1.31E-06	TMEM60
NA	7_77397906_ATAT_A_b37	ENSG00000135211	1.26E-05	TMEM60
rs11972122	7_77399800_G_C_b37	ENSG00000135211	3.71E-06	TMEM60
rs11979172	7_77399876_C_T_b37	ENSG00000135211	3.25E-06	TMEM60
rs4729725	7_77400524_C_A_b37	ENSG00000135211	4.00E-06	TMEM60
rs4729726	7_77400587_G_C_b37	ENSG00000135211	2.28E-06	TMEM60
rs4729727	7_77400589_T_G_b37	ENSG00000135211	2.82E-06	TMEM60
rs6465816	7_77401307_T_C_b37	ENSG00000135211	6.22E-06	TMEM60
rs201951010	7_77405568_T_TC_b37	ENSG00000135211	2.87E-06	TMEM60
rs2868813	7_77405741_T_C_b37	ENSG00000135211	7.02E-06	TMEM60
rs28716648	7_77409382_T_C_b37	ENSG00000135211	4.49E-06	TMEM60
rs6954671	7_77410314_G_C_b37	ENSG00000135211	2.71E-06	TMEM60
rs6958015	7_77413785_A_G_b37	ENSG00000135211	9.63E-08	TMEM60
NA	7_77414652_TG_T_b37	ENSG00000135211	2.81E-05	TMEM60
rs10242184	7_77415540_C_G_b37	ENSG00000135211	5.16E-07	TMEM60

rs6949795	7_77416947_T_C_b37	ENSG00000135211	3.65E-07	TMEM60
rs1544459	7_77417584_T_C_b37	ENSG00000135211	2.19E-05	TMEM60
rs1544458	7_77417701_A_G_b37	ENSG00000135211	5.06E-07	TMEM60
rs1544457	7_77419290_G_T_b37	ENSG00000135211	6.69E-07	TMEM60
NA	7_77420171_AC_A_b37	ENSG00000135211	5.25E-07	TMEM60
rs2057932	7_77421594_C_A_b37	ENSG00000135211	6.65E-07	TMEM60
rs6465828	7_77421817_G_T_b37	ENSG00000135211	6.64E-07	TMEM60
rs7953	7_77423574_G_A_b37	ENSG00000135211	1.38E-07	TMEM60
rs6970158	7_77426247_T_G_b37	ENSG00000135211	2.03E-05	TMEM60
rs9769871	7_77449861_T_G_b37	ENSG00000135211	2.95E-07	TMEM60
rs10248334	7_77451812_C_T_b37	ENSG00000135211	1.07E-07	TMEM60
rs6465848	7_77454104_T_C_b37	ENSG00000135211	8.06E-07	TMEM60
rs1526748	7_77467208_G_A_b37	ENSG00000135211	9.48E-08	TMEM60
rs2091402	7_77477002_A_G_b37	ENSG00000135211	2.99E-07	TMEM60
rs36055510	7_77479318_T_TTC_b37	ENSG00000135211	1.19E-06	TMEM60
rs10248871	7_77480571_G_A_b37	ENSG00000135211	1.40E-07	TMEM60
rs1405420	7_77486234_T_G_b37	ENSG00000135211	8.09E-07	TMEM60
rs7802691	7_77491234_T_C_b37	ENSG00000135211	4.22E-07	TMEM60
NA	7_77491388_AG_A_b37	ENSG00000135211	1.40E-06	TMEM60
rs10272350	7_77491389_G_A_b37	ENSG00000135211	6.53E-07	TMEM60
rs6465867	7_77497472_T_C_b37	ENSG00000135211	1.26E-07	TMEM60
rs7803160	7_77497720_A_G_b37	ENSG00000135211	2.92E-07	TMEM60
rs2471584	7_77504095_G_C_b37	ENSG00000135211	1.82E-06	TMEM60
NA	7_77504847_ATATT_A_b37	ENSG00000135211	6.96E-06	TMEM60
rs2463008	7_77509874_T_C_b37	ENSG00000135211	2.00E-06	TMEM60
rs2463010	7_77513885_T_C_b37	ENSG00000135211	1.93E-06	TMEM60
rs2463011	7_77514389_A_C_b37	ENSG00000135211	1.95E-06	TMEM60
rs10267180	7_77523521_A_G_b37	ENSG00000135211	3.17E-07	TMEM60
rs10252383	7_77523705_C_T_b37	ENSG00000135211	8.60E-07	TMEM60
rs2471603	7_77525199_T_C_b37	ENSG00000135211	5.91E-07	TMEM60
rs848449	7_77527456_A_G_b37	ENSG00000135211	9.10E-08	TMEM60
rs848493	7_77532521_A_C_b37	ENSG00000135211	1.03E-07	TMEM60
rs711309	7_77539744_G_A_b37	ENSG00000135211	1.04E-07	TMEM60
rs848478	7_77544019_T_G_b37	ENSG00000135211	9.43E-08	TMEM60
rs848481	7_77545104_T_A_b37	ENSG00000135211	3.50E-07	TMEM60
rs848482	7_77545526_A_C_b37	ENSG00000135211	3.18E-07	TMEM60
rs848484	7_77549321_C_A_b37	ENSG00000135211	8.49E-07	TMEM60
rs848485	7_77551482_G_T_b37	ENSG00000135211	8.49E-07	TMEM60
rs848489	7_77554622_G_A_b37	ENSG00000135211	8.49E-07	TMEM60
rs848491	7_77555541_C_G_b37	ENSG00000135211	8.75E-07	TMEM60
rs1725749	7_77561374_C_T_b37	ENSG00000135211	3.32E-07	TMEM60
NA	7_77561902_GAAGAGAGAGAGAG	ENSG00000135211	4.72E-07	TMEM60
rs74223569	7_77572427_C_CT_b37	ENSG00000135211	1.97E-06	TMEM60
NA	7_77572824_CTTAG_C_b37	ENSG00000135211	8.24E-07	TMEM60
rs34160426	7_77577818_A_C_b37	ENSG00000135211	1.80E-06	TMEM60
rs848461	7_77582265_T_C_b37	ENSG00000135211	5.52E-07	TMEM60
rs848462	7_77582663_A_G_b37	ENSG00000135211	5.02E-07	TMEM60

rs861049	7_77591922_G_C_b37	ENSG00000135211	2.48E-06	TMEM60
rs848465	7_77591968_G_A_b37	ENSG00000135211	2.24E-05	TMEM60
rs848466	7_77592607_T_C_b37	ENSG00000135211	1.76E-07	TMEM60
rs36119388	7_77593448_G_GA_b37	ENSG00000135211	4.15E-07	TMEM60
rs848468	7_77593814_C_T_b37	ENSG00000135211	1.15E-07	TMEM60
rs848469	7_77593841_C_T_b37	ENSG00000135211	3.26E-06	TMEM60
rs848470	7_77594007_C_G_b37	ENSG00000135211	3.50E-06	TMEM60
rs848471	7_77594550_C_A_b37	ENSG00000135211	3.42E-07	TMEM60
rs848474	7_77594902_G_A_b37	ENSG00000135211	3.54E-06	TMEM60
rs1205284	7_77595308_C_T_b37	ENSG00000135211	3.35E-06	TMEM60
rs848451	7_77596047_C_T_b37	ENSG00000135211	3.82E-06	TMEM60
rs848453	7_77597488_T_G_b37	ENSG00000135211	3.85E-06	TMEM60
rs1636676	7_77598053_C_T_b37	ENSG00000135211	3.00E-07	TMEM60
rs1636677	7_77598537_C_T_b37	ENSG00000135211	1.39E-06	TMEM60
rs1725745	7_77598538_A_C_b37	ENSG00000135211	1.39E-06	TMEM60
rs200190900	7_77602198_G_GGT_b37	ENSG00000135211	5.84E-08	TMEM60
rs201333966	7_77602202_T_TG_b37	ENSG00000135211	1.02E-07	TMEM60
rs848455	7_77602377_C_T_b37	ENSG00000135211	7.21E-06	TMEM60
rs848457	7_77602503_G_T_b37	ENSG00000135211	1.28E-06	TMEM60
rs848458	7_77602832_A_G_b37	ENSG00000135211	5.50E-08	TMEM60
rs1100236	7_77603385_G_A_b37	ENSG00000135211	2.13E-06	TMEM60
rs34022094	7_77605202_G_GA_b37	ENSG00000135211	8.62E-08	TMEM60
rs1100237	7_77606013_T_C_b37	ENSG00000135211	1.03E-05	TMEM60
rs2957025	8_82522763_T_C_b37	ENSG00000104231	8.55E-06	ZFAND1
rs2957023	8_82524624_G_A_b37	ENSG00000104231	2.51E-05	ZFAND1
rs2955029	8_82524678_C_T_b37	ENSG00000104231	2.49E-05	ZFAND1
rs2957022	8_82525121_G_A_b37	ENSG00000104231	2.49E-05	ZFAND1
rs2955028	8_82525240_C_T_b37	ENSG00000104231	2.49E-05	ZFAND1
NA	8_82525656_CACACAT_C_b37	ENSG00000104231	1.36E-05	ZFAND1
rs2919300	8_82525819_C_A_b37	ENSG00000104231	2.30E-05	ZFAND1
rs2919298	8_82527678_C_T_b37	ENSG00000104231	2.49E-05	ZFAND1
rs2955027	8_82529812_C_T_b37	ENSG00000104231	2.49E-05	ZFAND1
rs2912822	8_82529873_G_A_b37	ENSG00000104231	2.49E-05	ZFAND1
rs2955026	8_82530641_C_T_b37	ENSG00000104231	7.23E-06	ZFAND1
rs2955025	8_82530908_T_C_b37	ENSG00000104231	2.49E-05	ZFAND1
rs2955024	8_82530925_G_C_b37	ENSG00000104231	3.09E-05	ZFAND1
rs919186	8_82531805_T_C_b37	ENSG00000104231	8.55E-06	ZFAND1
rs2955023	8_82534053_G_A_b37	ENSG00000104231	8.55E-06	ZFAND1
rs2919299	8_82534868_G_A_b37	ENSG00000104231	2.49E-05	ZFAND1
rs2860289	8_82536323_G_A_b37	ENSG00000104231	2.39E-05	ZFAND1
rs737225	8_82536460_A_C_b37	ENSG00000104231	2.39E-05	ZFAND1
rs2957021	8_82537056_C_G_b37	ENSG00000104231	1.81E-05	ZFAND1
rs202126998	8_82537250_T_TGC_b37	ENSG00000104231	2.50E-05	ZFAND1
rs2400593	8_82537257_A_G_b37	ENSG00000104231	2.40E-05	ZFAND1
rs2294005	8_82539212_C_A_b37	ENSG00000104231	2.49E-05	ZFAND1
rs2294007	8_82539402_A_G_b37	ENSG00000104231	2.49E-05	ZFAND1
rs2991006	8_82544274_G_C_b37	ENSG00000104231	2.49E-05	ZFAND1

rs2991007	8_82544382_G_A_b37	ENSG00000104231	2.49E-05	ZFAND1
rs2955022	8_82545238_A_G_b37	ENSG00000104231	2.49E-05	ZFAND1
rs890601	8_82545437_C_T_b37	ENSG00000104231	2.49E-05	ZFAND1
rs2912820	8_82546240_C_T_b37	ENSG00000104231	2.49E-05	ZFAND1
rs2957020	8_82546266_G_T_b37	ENSG00000104231	2.49E-05	ZFAND1
rs2912819	8_82547153_G_A_b37	ENSG00000104231	2.49E-05	ZFAND1
rs2912818	8_82547211_G_A_b37	ENSG00000104231	2.49E-05	ZFAND1
rs2975941	8_82547364_A_T_b37	ENSG00000104231	2.49E-05	ZFAND1
rs2975940	8_82547478_G_A_b37	ENSG00000104231	2.49E-05	ZFAND1
rs146500857	8_82547909_A_AAC_b37	ENSG00000104231	2.49E-05	ZFAND1
rs2957019	8_82547932_A_G_b37	ENSG00000104231	2.49E-05	ZFAND1
rs2912816	8_82548344_C_G_b37	ENSG00000104231	2.49E-05	ZFAND1
rs2957018	8_82549124_G_A_b37	ENSG00000104231	2.49E-05	ZFAND1
rs2975939	8_82549521_G_A_b37	ENSG00000104231	2.49E-05	ZFAND1
rs2912815	8_82549829_C_A_b37	ENSG00000104231	2.49E-05	ZFAND1
NA	8_82550444_TG_T_b37	ENSG00000104231	2.54E-05	ZFAND1
rs201542649	8_82550445_GT_G_b37	ENSG00000104231	2.49E-05	ZFAND1
rs2975938	8_82550680_G_C_b37	ENSG00000104231	2.49E-05	ZFAND1
rs2912814	8_82551444_C_A_b37	ENSG00000104231	2.49E-05	ZFAND1
rs56210765	8_82551754_A_T_b37	ENSG00000104231	2.49E-05	ZFAND1
rs55640701	8_82551928_G_C_b37	ENSG00000104231	2.49E-05	ZFAND1
rs2957016	8_82552219_A_G_b37	ENSG00000104231	2.49E-05	ZFAND1
rs2957015	8_82552460_T_C_b37	ENSG00000104231	2.31E-05	ZFAND1
rs2957014	8_82552461_A_G_b37	ENSG00000104231	2.31E-05	ZFAND1
rs2912813	8_82552558_T_G_b37	ENSG00000104231	2.46E-05	ZFAND1
rs726605	8_82553023_C_T_b37	ENSG00000104231	2.49E-05	ZFAND1
rs726604	8_82553076_C_T_b37	ENSG00000104231	2.49E-05	ZFAND1
rs2912812	8_82553311_C_G_b37	ENSG00000104231	2.94E-05	ZFAND1
rs2912811	8_82553457_T_G_b37	ENSG00000104231	2.85E-05	ZFAND1
NA	8_82553624_CAAAT_C_b37	ENSG00000104231	2.83E-05	ZFAND1
rs2975936	8_82553921_A_C_b37	ENSG00000104231	2.49E-05	ZFAND1
rs2975934	8_82554012_A_T_b37	ENSG00000104231	2.49E-05	ZFAND1
rs2912810	8_82554221_T_C_b37	ENSG00000104231	2.49E-05	ZFAND1
rs2912809	8_82554568_A_G_b37	ENSG00000104231	2.49E-05	ZFAND1
rs200203173	8_82554989_G_GAAC_b37	ENSG00000104231	5.53E-05	ZFAND1
rs149127221	8_82554992_G_GA_b37	ENSG00000104231	6.99E-05	ZFAND1
NA	8_82554995_AG_A_b37	ENSG00000104231	6.28E-05	ZFAND1
rs2975932	8_82555129_G_A_b37	ENSG00000104231	2.49E-05	ZFAND1
rs201074960	8_82555506_G_GC_b37	ENSG00000104231	2.91E-05	ZFAND1
rs35000792	8_82555508_C_CA_b37	ENSG00000104231	2.50E-05	ZFAND1
rs2957012	8_82555662_G_C_b37	ENSG00000104231	2.45E-05	ZFAND1
rs2912808	8_82556120_G_A_b37	ENSG00000104231	2.49E-05	ZFAND1
rs2912807	8_82556146_G_A_b37	ENSG00000104231	2.49E-05	ZFAND1
rs2912806	8_82556653_A_G_b37	ENSG00000104231	2.49E-05	ZFAND1
rs878326	8_82557608_C_G_b37	ENSG00000104231	2.49E-05	ZFAND1
rs878327	8_82557691_G_A_b37	ENSG00000104231	2.49E-05	ZFAND1
rs2081758	8_82557894_C_T_b37	ENSG00000104231	2.49E-05	ZFAND1

rs2955019	8_82558423_C_T_b37	ENSG00000104231	2.46E-05	ZFAND1
rs2955018	8_82558681_C_T_b37	ENSG00000104231	2.79E-05	ZFAND1
rs2955017	8_82558735_T_C_b37	ENSG00000104231	2.46E-05	ZFAND1
rs2955016	8_82562290_A_G_b37	ENSG00000104231	2.46E-05	ZFAND1
rs2955014	8_82571434_C_A_b37	ENSG00000104231	2.46E-05	ZFAND1
rs2162546	8_82573580_C_T_b37	ENSG00000104231	2.46E-05	ZFAND1
NA	8_82573609_CTACCAT_C_b37	ENSG00000104231	2.93E-05	ZFAND1
rs2300493	8_82578925_A_G_b37	ENSG00000104231	1.95E-05	ZFAND1
rs58550159	8_82579968_C_CTGAG_b37	ENSG00000104231	2.91E-05	ZFAND1
rs2957010	8_82581392_G_C_b37	ENSG00000104231	2.46E-05	ZFAND1
rs2268432	8_82586777_G_T_b37	ENSG00000104231	2.46E-05	ZFAND1
rs2268431	8_82587368_C_T_b37	ENSG00000104231	1.07E-05	ZFAND1
rs2217173	8_82588888_T_A_b37	ENSG00000104231	2.46E-05	ZFAND1
rs2912803	8_82595445_G_A_b37	ENSG00000104231	2.44E-05	ZFAND1
rs188805868	8_82597250_G_A_b37	ENSG00000104231	3.59E-05	ZFAND1
rs2912817	8_82600027_A_T_b37	ENSG00000104231	5.35E-05	ZFAND1
rs2955009	8_82600151_G_A_b37	ENSG00000104231	2.49E-05	ZFAND1
rs2957013	8_82600853_G_A_b37	ENSG00000104231	2.49E-05	ZFAND1
rs2912805	8_82601240_A_G_b37	ENSG00000104231	2.21E-05	ZFAND1
rs2955007	8_82602348_C_A_b37	ENSG00000104231	2.48E-05	ZFAND1
rs35277559	8_82603480_G_A_b37	ENSG00000104231	1.75E-05	ZFAND1
rs2955002	8_82608878_A_G_b37	ENSG00000104231	2.48E-05	ZFAND1
rs2991001	8_82611657_T_A_b37	ENSG00000104231	2.49E-05	ZFAND1
rs2955015	8_82614003_A_C_b37	ENSG00000104231	2.49E-05	ZFAND1
rs2955011	8_82616537_T_C_b37	ENSG00000104231	5.56E-05	ZFAND1
rs201739557	8_82617598_A_AAAT_b37	ENSG00000104231	1.43E-05	ZFAND1
rs201188948	8_82617599_A_AATT_b37	ENSG00000104231	1.43E-05	ZFAND1
rs2955005	8_82618254_A_C_b37	ENSG00000104231	4.19E-05	ZFAND1
rs5892769	8_82618680_T_TA_b37	ENSG00000104231	2.49E-05	ZFAND1
rs2046786	8_82621781_T_G_b37	ENSG00000104231	2.49E-05	ZFAND1
rs62512326	8_82623890_T_C_b37	ENSG00000104231	2.49E-05	ZFAND1
rs60319049	8_82625478_G_A_b37	ENSG00000104231	3.48E-05	ZFAND1
rs58900840	8_82627395_A_C_b37	ENSG00000104231	4.19E-05	ZFAND1
rs16909510	8_82629906_G_A_b37	ENSG00000104231	2.49E-05	ZFAND1
rs2130263	8_82633452_C_G_b37	ENSG00000104231	2.50E-05	ZFAND1
rs3735701	8_82644442_G_A_b37	ENSG00000104231	2.09E-05	ZFAND1
rs9643764	8_83386881_T_C_b37	ENSG00000104231	5.85E-05	ZFAND1
rs10087936	8_83388326_A_G_b37	ENSG00000104231	4.90E-05	ZFAND1
rs77015628	13_39504538_C_T_b37	ENSG00000183722	1.79E-05	LHFP
rs76016631	13_39526371_G_C_b37	ENSG00000183722	6.41E-06	LHFP
rs7998659	13_39528811_C_T_b37	ENSG00000183722	3.52E-05	LHFP
rs143670014	13_39530618_T_C_b37	ENSG00000183722	2.77E-05	LHFP
rs7985312	13_39533661_C_G_b37	ENSG00000183722	3.57E-05	LHFP
rs6563654	13_39535572_T_C_b37	ENSG00000183722	3.67E-05	LHFP
rs7326584	13_39539323_T_C_b37	ENSG00000183722	3.68E-05	LHFP
rs4941906	13_39686263_C_G_b37	ENSG00000183722	4.71E-05	LHFP
rs4383024	13_39720798_G_T_b37	ENSG00000183722	7.54E-06	LHFP

rs4485232	13_39721129_A_T_b37	ENSG00000183722	5.78E-06	LHFP
rs75203989	13_39729428_C_T_b37	ENSG00000183722	8.48E-06	LHFP
rs17444010	13_39745892_C_T_b37	ENSG00000183722	2.22E-06	LHFP
rs3927576	13_39749624_C_G_b37	ENSG00000183722	5.85E-06	LHFP
rs3927577	13_39749754_C_G_b37	ENSG00000183722	1.48E-05	LHFP
rs7992745	13_39750239_G_T_b37	ENSG00000183722	1.37E-05	LHFP
rs7999969	13_39750665_A_G_b37	ENSG00000183722	3.42E-05	LHFP
rs4471550	13_39750811_T_C_b37	ENSG00000183722	3.34E-05	LHFP
rs1333440	13_39751029_T_C_b37	ENSG00000183722	1.64E-05	LHFP
rs12870208	13_39751649_T_G_b37	ENSG00000183722	2.50E-05	LHFP
rs7983684	13_39751711_C_T_b37	ENSG00000183722	2.48E-05	LHFP
rs1333445	13_39751789_A_G_b37	ENSG00000183722	3.38E-06	LHFP
rs7989167	13_39751818_T_C_b37	ENSG00000183722	2.47E-05	LHFP
rs7982609	13_39751820_G_A_b37	ENSG00000183722	2.48E-05	LHFP
rs1981060	13_39752044_A_G_b37	ENSG00000183722	2.46E-05	LHFP
rs1930818	13_39752675_G_T_b37	ENSG00000183722	2.37E-05	LHFP
rs4474575	13_39755977_C_T_b37	ENSG00000183722	4.50E-05	LHFP
rs9548655	13_39756211_A_G_b37	ENSG00000183722	2.07E-09	LHFP
rs117880346	13_39759067_G_C_b37	ENSG00000183722	1.32E-05	LHFP
rs9532340	13_39763310_G_A_b37	ENSG00000183722	9.34E-10	LHFP
rs9532341	13_39763314_C_A_b37	ENSG00000183722	3.33E-10	LHFP
rs55795822	13_39764046_T_A_b37	ENSG00000183722	8.88E-08	LHFP
rs4991626	13_39764128_G_A_b37	ENSG00000183722	1.28E-06	LHFP
rs9548659	13_39766459_C_A_b37	ENSG00000183722	4.25E-08	LHFP
rs9548660	13_39766740_G_A_b37	ENSG00000183722	3.58E-08	LHFP
rs12430730	13_39766794_T_C_b37	ENSG00000183722	3.62E-08	LHFP
rs9576752	13_39767037_G_T_b37	ENSG00000183722	8.69E-09	LHFP
rs9548661	13_39767508_T_C_b37	ENSG00000183722	4.10E-08	LHFP
rs4943645	13_39767750_C_A_b37	ENSG00000183722	4.37E-08	LHFP
rs79433147	13_39767948_T_G_b37	ENSG00000183722	3.44E-08	LHFP
rs9532343	13_39769379_C_T_b37	ENSG00000183722	8.83E-09	LHFP
rs9566407	13_39769690_T_C_b37	ENSG00000183722	1.73E-06	LHFP
rs9566408	13_39769871_T_C_b37	ENSG00000183722	2.00E-08	LHFP
rs6563685	13_39770104_G_A_b37	ENSG00000183722	5.49E-07	LHFP
rs9548662	13_39770273_C_A_b37	ENSG00000183722	1.04E-06	LHFP
NA	13_39771445_AC_A_b37	ENSG00000183722	9.48E-07	LHFP
NA	13_39772177_AT_A_b37	ENSG00000183722	1.42E-08	LHFP
rs9576753	13_39772615_G_T_b37	ENSG00000183722	7.95E-09	LHFP
rs9566409	13_39772687_T_A_b37	ENSG00000183722	2.95E-07	LHFP
rs4374042	13_39773999_G_A_b37	ENSG00000183722	3.33E-07	LHFP
rs7989670	13_39774309_C_T_b37	ENSG00000183722	3.38E-07	LHFP
rs7990362	13_39774360_A_G_b37	ENSG00000183722	3.39E-07	LHFP
rs7990373	13_39774380_A_G_b37	ENSG00000183722	3.96E-07	LHFP
rs7332946	13_39776677_C_T_b37	ENSG00000183722	2.54E-07	LHFP
rs7333515	13_39776923_C_T_b37	ENSG00000183722	2.54E-07	LHFP
rs9576754	13_39777253_C_T_b37	ENSG00000183722	2.94E-06	LHFP
rs9566410	13_39777255_G_A_b37	ENSG00000183722	2.53E-07	LHFP

rs9548665	13_39777741_T_C_b37	ENSG00000183722	2.74E-07	LHFP
rs9548666	13_39777891_A_T_b37	ENSG00000183722	2.54E-07	LHFP
rs9548667	13_39777950_C_A_b37	ENSG00000183722	2.54E-07	LHFP
rs17628199	13_39778125_C_A_b37	ENSG00000183722	2.55E-07	LHFP
rs113788332	13_39778256_T_C_b37	ENSG00000183722	4.28E-07	LHFP
rs80290358	13_39778335_T_G_b37	ENSG00000183722	1.30E-05	LHFP
rs77648080	13_39778339_C_T_b37	ENSG00000183722	1.29E-05	LHFP
rs9532345	13_39778812_C_G_b37	ENSG00000183722	8.81E-09	LHFP
rs2026524	13_39779135_G_A_b37	ENSG00000183722	5.30E-08	LHFP
NA	13_39779321_GA_G_b37	ENSG00000183722	2.81E-10	LHFP
rs9576756	13_39779744_C_T_b37	ENSG00000183722	7.26E-09	LHFP
rs7330627	13_39779945_A_G_b37	ENSG00000183722	3.44E-06	LHFP
rs60345209	13_39780417_A_T_b37	ENSG00000183722	1.96E-06	LHFP
rs9532346	13_39780686_T_C_b37	ENSG00000183722	2.05E-06	LHFP
rs9548668	13_39780757_C_A_b37	ENSG00000183722	5.79E-06	LHFP
rs9532347	13_39780825_G_T_b37	ENSG00000183722	1.57E-09	LHFP
rs9548669	13_39781080_C_G_b37	ENSG00000183722	2.06E-06	LHFP
rs9532349	13_39781343_G_T_b37	ENSG00000183722	2.07E-06	LHFP
rs9548670	13_39781373_G_T_b37	ENSG00000183722	2.07E-06	LHFP
rs9576758	13_39782091_G_A_b37	ENSG00000183722	1.24E-07	LHFP
rs544151	13_39782440_T_C_b37	ENSG00000183722	1.86E-06	LHFP
rs9548673	13_39782598_T_C_b37	ENSG00000183722	1.26E-07	LHFP
rs9548674	13_39782894_T_C_b37	ENSG00000183722	1.88E-06	LHFP
rs9548675	13_39783095_C_T_b37	ENSG00000183722	1.50E-09	LHFP
rs9315663	13_39783130_C_T_b37	ENSG00000183722	1.37E-06	LHFP
rs9548676	13_39783490_C_T_b37	ENSG00000183722	1.88E-06	LHFP
rs9548677	13_39783549_G_A_b37	ENSG00000183722	1.88E-06	LHFP
rs9548678	13_39783704_C_G_b37	ENSG00000183722	1.88E-06	LHFP
rs9548679	13_39783727_A_T_b37	ENSG00000183722	1.88E-06	LHFP
rs9548680	13_39783752_G_A_b37	ENSG00000183722	1.88E-06	LHFP
rs112678289	13_39783828_T_TA_b37	ENSG00000183722	1.37E-09	LHFP
rs9532350	13_39783952_A_G_b37	ENSG00000183722	1.88E-06	LHFP
rs9532351	13_39783959_C_T_b37	ENSG00000183722	1.88E-06	LHFP
rs9532352	13_39784092_T_G_b37	ENSG00000183722	1.88E-06	LHFP
rs9315664	13_39784382_C_A_b37	ENSG00000183722	1.50E-09	LHFP
rs9548681	13_39784720_G_A_b37	ENSG00000183722	1.69E-06	LHFP
rs9548682	13_39784724_T_G_b37	ENSG00000183722	1.85E-06	LHFP
rs9532353	13_39784730_A_C_b37	ENSG00000183722	1.85E-06	LHFP
rs9548683	13_39785078_A_G_b37	ENSG00000183722	6.95E-06	LHFP
rs4362261	13_39785523_A_G_b37	ENSG00000183722	1.88E-06	LHFP
rs9532354	13_39786449_G_A_b37	ENSG00000183722	1.27E-07	LHFP
rs12430343	13_39786736_A_G_b37	ENSG00000183722	2.23E-09	LHFP
rs12428316	13_39786838_G_A_b37	ENSG00000183722	1.91E-06	LHFP
rs9532355	13_39787679_T_C_b37	ENSG00000183722	5.28E-06	LHFP
rs9532356	13_39787689_A_C_b37	ENSG00000183722	5.27E-06	LHFP
rs60790869	13_39787739_C_CT_b37	ENSG00000183722	9.75E-06	LHFP
rs9548684	13_39788087_C_T_b37	ENSG00000183722	2.69E-06	LHFP

rs66781018	13_39788293_T_C_b37	ENSG00000183722	2.81E-06	LHFP
rs35009897	13_39788437_G_GAT_b37	ENSG00000183722	8.54E-06	LHFP
rs56233557	13_39789579_A_G_b37	ENSG00000183722	3.91E-06	LHFP
rs9532357	13_39793634_C_A_b37	ENSG00000183722	2.62E-05	LHFP
rs9548687	13_39794354_A_T_b37	ENSG00000183722	2.63E-05	LHFP
rs9548690	13_39797447_A_T_b37	ENSG00000183722	2.60E-05	LHFP
rs17528800	13_39799745_A_T_b37	ENSG00000183722	3.29E-05	LHFP
rs12429285	13_39800885_C_T_b37	ENSG00000183722	3.29E-05	LHFP
rs1577030	13_39802502_G_T_b37	ENSG00000183722	2.98E-05	LHFP
rs944863	13_39803262_A_G_b37	ENSG00000183722	1.17E-05	LHFP
rs944864	13_39803379_A_T_b37	ENSG00000183722	2.86E-05	LHFP
rs11838834	13_39805533_T_C_b37	ENSG00000183722	2.86E-05	LHFP
rs9548696	13_39806196_G_A_b37	ENSG00000183722	2.86E-05	LHFP
rs9548697	13_39806665_G_A_b37	ENSG00000183722	5.57E-06	LHFP
rs9548698	13_39807932_T_C_b37	ENSG00000183722	1.75E-05	LHFP
rs9548701	13_39809786_A_G_b37	ENSG00000183722	1.09E-05	LHFP
rs9548702	13_39811186_T_C_b37	ENSG00000183722	3.54E-05	LHFP
rs12427917	13_40906683_G_A_b37	ENSG00000183722	1.40E-05	LHFP
rs149201670	13_40909122_G_A_b37	ENSG00000183722	2.25E-05	LHFP
rs12428360	13_40913362_G_A_b37	ENSG00000183722	3.99E-05	LHFP
rs2701858	13_41138389_C_T_b37	ENSG00000120688	3.92E-05	WBP4
rs2701865	13_41163373_A_G_b37	ENSG00000120688	2.36E-05	WBP4
rs7333119	13_41257371_A_G_b37	ENSG00000120688	1.14E-05	WBP4
rs199565134	13_41259647_T_TG_b37	ENSG00000120688	3.96E-05	WBP4
rs9532585	13_41268086_A_G_b37	ENSG00000120688	8.77E-06	WBP4
NA	13_41269413_AG_A_b37	ENSG00000120688	3.38E-05	WBP4
rs4943805	13_41270080_A_G_b37	ENSG00000120688	4.95E-05	WBP4
rs113001496	13_41270293_T_C_b37	ENSG00000120688	2.75E-05	WBP4
rs7323670	13_41276932_C_G_b37	ENSG00000120688	1.78E-05	WBP4
rs9566566	13_41277470_A_T_b37	ENSG00000120688	2.69E-05	WBP4
rs13378732	13_41278802_G_A_b37	ENSG00000120688	2.69E-05	WBP4
rs10161872	13_41279626_A_G_b37	ENSG00000120688	2.70E-05	WBP4
rs7990957	13_41280979_A_G_b37	ENSG00000120688	2.11E-05	WBP4
NA	13_41282159_GT_G_b37	ENSG00000120688	9.24E-06	WBP4
rs9549269	13_41283423_T_G_b37	ENSG00000120688	5.84E-05	WBP4
rs9549270	13_41284193_G_T_b37	ENSG00000120688	4.44E-05	WBP4
rs9315782	13_41289110_G_A_b37	ENSG00000120688	4.44E-05	WBP4
rs9315783	13_41289164_T_C_b37	ENSG00000120688	4.28E-05	WBP4
rs9315784	13_41289166_G_A_b37	ENSG00000120688	4.28E-05	WBP4
rs9566569	13_41291240_A_T_b37	ENSG00000120688	1.46E-05	WBP4
rs9577123	13_41293052_A_G_b37	ENSG00000120688	1.59E-05	WBP4
rs9549271	13_41295477_T_C_b37	ENSG00000120688	5.29E-05	WBP4
rs6563844	13_41298190_T_C_b37	ENSG00000120688	4.00E-05	WBP4
rs61963345	13_41299129_T_C_b37	ENSG00000120688	4.43E-05	WBP4
rs9577126	13_41301558_C_A_b37	ENSG00000120688	1.59E-05	WBP4
rs13378489	13_41301852_T_C_b37	ENSG00000120688	1.59E-05	WBP4
rs9532588	13_41302913_G_A_b37	ENSG00000120688	5.06E-05	WBP4

rs9577129	13_41306884_A_G_b37	ENSG0000012068€	1.59E-05	WBP4
rs9646110	13_41308301_T_A_b37	ENSG0000012068€	4.45E-05	WBP4
rs74505554	13_41311443_A_T_b37	ENSG0000012068€	4.38E-05	WBP4
rs9577134	13_41315995_C_T_b37	ENSG0000012068€	1.58E-05	WBP4
rs9549280	13_41322660_T_C_b37	ENSG0000012068€	3.98E-05	WBP4
rs8000482	13_41324623_A_T_b37	ENSG0000012068€	1.35E-05	WBP4
rs7992974	13_41326559_A_G_b37	ENSG0000012068€	1.35E-05	WBP4
rs9549282	13_41331895_T_A_b37	ENSG0000012068€	4.09E-05	WBP4
rs4941991	13_41335719_T_C_b37	ENSG0000012068€	5.22E-05	WBP4
rs117823156	13_41411314_G_A_b37	ENSG0000012068€	1.69E-05	WBP4
rs73174839	13_41411575_T_C_b37	ENSG0000012068€	2.05E-05	WBP4
rs181859107	13_41412918_C_T_b37	ENSG0000012068€	6.15E-05	WBP4
rs148593766	13_41414235_T_C_b37	ENSG0000012068€	5.37E-05	WBP4
rs55646133	13_41415251_G_A_b37	ENSG0000012068€	2.56E-05	WBP4
rs7992208	13_41416221_T_C_b37	ENSG0000012068€	5.88E-05	WBP4
rs58961104	13_41416263_A_G_b37	ENSG0000012068€	3.48E-05	WBP4
rs56120794	13_41446719_A_G_b37	ENSG0000012068€	3.27E-06	WBP4
rs17447487	13_41495245_C_T_b37	ENSG0000012068€	7.04E-06	WBP4
rs55780081	13_41533495_G_A_b37	ENSG0000012068€	3.53E-05	WBP4
rs9532701	13_41588462_T_C_b37	ENSG0000012068€	3.04E-05	WBP4
rs73176937	13_41598028_T_A_b37	ENSG0000012068€	1.21E-09	WBP4
rs17532301	13_41609047_G_A_b37	ENSG0000012068€	1.58E-09	WBP4
rs73176948	13_41611271_A_G_b37	ENSG0000012068€	1.48E-09	WBP4
rs8002341	13_41615518_T_A_b37	ENSG0000012068€	2.86E-05	WBP4
rs55704414	13_41616345_G_C_b37	ENSG0000012068€	2.86E-05	WBP4
rs7329068	13_41628282_T_C_b37	ENSG0000012068€	2.85E-05	WBP4
rs17532371	13_41635301_C_G_b37	ENSG0000012068€	1.15E-09	WBP4
rs1329520	13_41638611_A_G_b37	ENSG0000012068€	4.78E-05	WBP4
rs17532413	13_41644956_A_C_b37	ENSG0000012068€	1.62E-06	WBP4
NA	13_41646085_CAG_C_b37	ENSG0000012068€	9.17E-10	WBP4
13:41652040	13_41652040_C_CA_b37	ENSG0000012068€	5.27E-10	WBP4
rs60223575	13_41653179_T_C_b37	ENSG0000012068€	5.00E-05	WBP4
rs61964864	13_41658975_A_G_b37	ENSG0000012068€	5.93E-05	WBP4
rs9532720	13_41659417_C_T_b37	ENSG0000012068€	5.93E-05	WBP4
rs78424108	13_41673457_C_G_b37	ENSG0000012068€	1.86E-09	WBP4
rs17532490	13_41683061_G_A_b37	ENSG0000012068€	9.71E-07	WBP4
rs7336347	13_41684306_G_A_b37	ENSG0000012068€	1.49E-07	WBP4
rs150641790	13_41687844_T_TG_b37	ENSG0000012068€	1.14E-08	WBP4
rs117230571	13_41689067_A_G_b37	ENSG0000012068€	2.58E-07	WBP4
rs9593087	13_76084537_C_A_b37	ENSG0000011893€	8.82E-06	UCHL3
rs7994189	13_76090298_A_G_b37	ENSG0000011893€	2.21E-06	UCHL3
rs6562900	13_76092621_T_C_b37	ENSG0000011893€	2.21E-06	UCHL3
rs6562901	13_76092829_C_T_b37	ENSG0000011893€	1.37E-06	UCHL3
rs7327341	13_76093078_G_A_b37	ENSG0000011893€	2.21E-06	UCHL3
rs7327055	13_76093098_C_T_b37	ENSG0000011893€	2.19E-06	UCHL3
rs7332853	13_76093772_A_G_b37	ENSG0000011893€	2.21E-06	UCHL3
rs7333191	13_76093845_C_T_b37	ENSG0000011893€	2.21E-06	UCHL3

rs8002157	13_76094545_C_G_b37	ENSG0000011893[illegible]	2.25E-06	UCHL3
rs35867883	18_55277951_T_C_b37	ENSG0000013444[illegible]	7.65E-06	NARS
rs9319918	18_55283236_T_C_b37	ENSG0000013444[illegible]	3.19E-06	NARS
rs9960462	18_55284332_G_A_b37	ENSG0000013444[illegible]	3.18E-06	NARS
NA	18_55284978_CT_C_b37	ENSG0000013444[illegible]	1.19E-05	NARS
rs7234963	18_55285123_G_A_b37	ENSG0000013444[illegible]	3.19E-06	NARS
rs2032938	18_55289076_C_T_b37	ENSG0000013444[illegible]	3.16E-06	NARS
rs2032937	18_55289180_A_G_b37	ENSG0000013444[illegible]	3.14E-06	NARS
rs9967179	18_55291682_C_T_b37	ENSG0000013444[illegible]	2.48E-06	NARS

Supplemental Table 5

For each gene, the probability that by chance the gene would be as connected to other differentially expressed genes as observed is provided as a p-value.

gene	p-value
YWHAZ	0.001997
HIST2H3C	0.001997
RPS25	0.001997
FLNA	0.001997
HIST2H3A	0.001997
GADD45A	0.001997
MPHOSPH	0.001997
NOL6	0.001997
HIST2H3D	0.001997
HIST1H3J	0.001997
HIST2H2A/	0.001997
IAH1	0.001997
NOP14	0.001997
PDHB	0.001997
HIST2H2A(	0.001997
HIST2H2A/	0.003992
RRP12	0.003992
KRR1	0.003992
FASN	0.003992
TMEM170/	0.005985
PSMD9	0.009965
PSMD5	0.011952
HSPA1B	0.01592
RSL24D1	0.017901
TSN	0.025805
PPP1R15B	0.031713
HSPA1A	0.033678
PSMA1	0.035641
BOP1	0.039561
WDR74	0.041518
ZNF354A	0.043473
UCHL1	0.043473
STRADB	0.045426
ACAT2	0.047377
GLUD1	0.049326
TANK	0.049326
PSMB4	0.055162
FKBP3	0.055162
PSMC6	0.060979
EIF3M	0.060979

TRIM28	0.068708
TELO2	0.07256
PLAC8	0.076404
TXN	0.08024
DHRS1	0.08598
GBP1	0.087889
ARID2	0.091702
NBEAL1	0.103091
FAM120A	0.12004
YEATS4	0.133111
SLC30A7	0.140536
LPCAT1	0.15529
ETFA	0.157126
SLC35B4	0.168096
ZC3HAV1	0.177182
PCDHA4	0.178994
NARS	0.188021
FMO4	0.196998
ACP1	0.198787
COMMD8	0.213031
NUPL2	0.22363
KIF11	0.225389
ORAI2	0.230655
LIAS	0.249812
MKI67	0.253269
DIABLO	0.261877
QTRT1	0.277245
CCDC109B	0.287401
SNX24	0.30083
MTHFD2	0.30083
DDX51	0.310818
ACOT9	0.328943
ACTR10	0.346826
UCHL3	0.35968
COX17	0.36606
BRD7	0.388135
COMMD1	0.405206
C16orf80	0.406746
HIC1	0.428095
DLD	0.432619
ARHGAP4	0.432619
FH	0.437125
SCCPDH	0.447569
POLD3	0.452016
FNTA	0.495489
GLUD2	0.501149
CCNF	0.506778

UBLCP1	0.50958
MLF1IP	0.55733
WBP4	0.56527
DSCC1	0.567901
DNM2	0.571832
AHCTF1	0.603893
ARMC6	0.607656
PNN	0.617604
PLRG1	0.631074
LBR	0.632287
PRKRA	0.633497
NUP54	0.63832
HSD17B11	0.693697
PRIM1	0.703568
RCN2	0.70574
NDUFS2	0.713278
AKAP5	0.714347
RFXAP	0.747496
POLR3F	0.755464
CASP6	0.7594
TRIP4	0.771017
MTFMT	0.782347
CRLS1	0.78974
TRIB1	0.825625
ZNF277	0.830595
NDUFB5	0.833053
PMPCB	0.861149
ZNF426	0.869218
NAE1	0.874227
TRMT1	0.876343
ZDHHC6	0.885308
SRGAP2	0.891975
SPOP	0.895879
RAB33B	0.897804
ZNF440	0.908374
NF1	0.914322
NQO2	0.917222
MAP2K6	0.917222
LCMT1	0.919505
UPF1	0.92287
SUFU	0.926706
PMAIP1	0.929915
EHD4	0.933569
SNRPA1	0.937625
DEGS1	0.939605
C9orf163	0.944885
CAMLG	0.951257

SGTB	0.951697
RPAIN	0.967665
BNIP2	0.971158
AAMP	0.972167
FYTTD1	0.972167
TNFSF10	0.973484
FAM103A1	0.9754
CTSS	0.977545
TCEA1	0.977545
ATP6V1D	0.986338
EAPP	0.986338
FPGT	0.986801
LIMD1	0.987924
SLC35E2	0.988143
LETM1	0.991916
ELMO1	0.993125
ZFAND1	0.993289
SDCBP	0.993928
MTHFD1L	0.994386
STX2	0.995783
COG5	0.996526
MED16	0.997701
DUSP11	0.998634
AGA	0.999272
ZNF326	0.999517
KLHDC2	0.999879
GRK6	0.999879
PLA1A	0.999936
AP3S1	1
IL3RA	1
LACTB2	NA
SFT2D1	NA
CD86	NA
S1PR4	NA
TMEM199	NA
MCCC1	NA
ZMYM5	NA
LY86	NA
SERINC1	NA
RNASE6	NA
ZBTB1	NA
PDCD1LG2	NA
GBP3	NA

Supplemental Table 6

Top 20 differentially expressed genes and survival analysis (logrank test p-value).

Gene	P-value	Note
ZFAND1	0.547	
LBR	0.488	
UCHL3	0.297	
FAM171B	0.223	
SFT2D1	0.271	
TMEM60	0.185	
C4orf33	0.525	
NDUFB5	0.677	
LHFP	Not Available	
NARS	0.0308	eQTL target gene in human lung
ZNF426	0.0211	
WBP4	0.751	
UCHL1	0.139	
CA5B	0.248	
AHCTF1	0.137	
BOP1	0.19	
MTFMT	0.207	
PSMC6	0.532	
C16orf80	Not Available	
TCEA1	0.023	

Supplemental Table 7

Imputation model for PEM response. The genes and their weights (in an additive model) are listed here.

**Gene**	**beta_from_imputation_model**
ANGEL1	-0.003178216
CAMSAP2	-0.102862488
DIS3L	0.357088844
FAM219B	-0.021069905
GOLGA8DP	-0.335002667
HIST1H2AC	0.042977472
HLA-DPA1	-0.126321185
NASP	0.074817427
NCAPD2	0.063126869
PIK3IP1	-0.217577032
RN7SL717P	0.085641033
SCML1	0.419162377
STX12	-0.003271198
SVIL	0.054157895
WBP1	-0.430982047
ZNF3	-0.09671555
ZNF446	-0.323953193
